# Genome Sequencing and Analysis of BCG Vaccine Strains

**DOI:** 10.1371/journal.pone.0071243

**Published:** 2013-08-19

**Authors:** Wen Zhang, Yuanyuan Zhang, Huajun Zheng, Yuanlong Pan, Haican Liu, Pengcheng Du, Li Wan, Jun Liu, Baoli Zhu, Guoping Zhao, Chen Chen, Kanglin Wan

**Affiliations:** 1 National Institute for Communicable Disease Control and Prevention, Chinese Center for Disease Control and Prevention/State Key Laboratory for Infectious Disease Prevention and Control, Beijing, China; 2 Collaborative Innovation Center for Diagnosis and Treatment of Infectious Diseases, Hangzhou, China; 3 Shanghai-MOST Key Laboratory of Health and Disease Genomics, Chinese National Human Genome Center at Shanghai, Shanghai, China; 4 CAS Key Lab of Pathogenic Microbiology and Immunology, Institute of Microbiology, Chinese Academy of Sciences, Beijing, China; 5 Key Laboratory of Medical Molecular Virology Affiliated to the Ministries of Education and Health, Shanghai Medical College; Department of Microbiology, School of Life Sciences, Fudan University, Shanghai, China; 6 Department of Molecular Genetics, University of Toronto, Toronto, Ontario, Canada; 7 Department of Microbiology and Li Ka Shing Institute of Health Sciences, The Chinese University of Hong Kong, Prince of Wales Hospital, Shatin, New Territories, Hong Kong SAR, China; 8 Key Laboratory of Synthetic Biology, Institute of Plant Physiology and Ecology, Shanghai Institutes for Biological Sciences, Chinese Academy of Sciences, Shanghai, China; Fundació Institut d'Investigació en Ciències de la Salut Germans Trias i Pujol. Universitat Autònoma de Barcelona. CIBERES, Spain

## Abstract

**Background:**

Although the Bacillus Calmette-Guérin (BCG) vaccine against tuberculosis (TB) has been available for more than 75 years, one third of the world's population is still infected with *Mycobacterium tuberculosis* and approximately 2 million people die of TB every year. To reduce this immense TB burden, a clearer understanding of the functional genes underlying the action of BCG and the development of new vaccines are urgently needed.

**Methods and Findings:**

Comparative genomic analysis of 19 *M. tuberculosis* complex strains showed that BCG strains underwent repeated human manipulation, had higher region of deletion rates than those of natural *M. tuberculosis* strains, and lost several essential components such as T-cell epitopes. A total of 188 BCG strain T-cell epitopes were lost to various degrees. The non-virulent BCG Tokyo strain, which has the largest number of T-cell epitopes (359), lost 124. Here we propose that BCG strain protection variability results from different epitopes. This study is the first to present BCG as a model organism for genetics research. BCG strains have a very well-documented history and now detailed genome information. Genome comparison revealed the selection process of BCG strains under human manipulation (1908–1966).

**Conclusions:**

Our results revealed the cause of BCG vaccine strain protection variability at the genome level and supported the hypothesis that the restoration of lost BCG Tokyo epitopes is a useful future vaccine development strategy. Furthermore, these detailed BCG vaccine genome investigation results will be useful in microbial genetics, microbial engineering and other research fields.

## Introduction

*Mycobacterium tuberculosis* is the world's leading cause of the infectious disease tuberculosis (TB) and have an enormous global impact [1]. The World Health Organization (WHO) claimed that an estimated 11.1 million people were newly infected with *M. tuberculosis* in 2008. In China alone, there were 200,614 deaths from TB in 2007.

Bacillus Calmette-Guérin (BCG), the world's most widely used vaccine against TB, is derived from *Mycobacterium bovis* and was attenuated after 230 passages over a period from 1908 to 1921 [2]. Since its attenuation, the original BCG strain has produced many descendant strains that have been distributed and used in many countries and regions around the world. These strains are named based on the country or corresponding site, e.g., BCG Tokyo, Pasteur, Russia. Although these BCG descendant strains share a common ancestor, each has markedly different characteristics since these strains have been propagated for >1,000 passages in different countries. In 1966, the WHO recommended that vaccines should not be prepared from cultures that had undergone >12 passages after culturing from a defined freeze-dried seed lot [3], [4].

Studies have reported that the estimates of the protection against TB imparted by BCG strains varied widely (0–80%) [5], [6], [7]. The greatest protection reported in the UK (∼80%) by the Medical Research Council is strikingly different from trials by the US Public Health Service in Georgia, Alabama and Puerto Rico, all of which recorded protection of <30% [7]. Several factors, such as genetic differences in the BCG strains used for immunization [2], environmental influences [7] and host genetic factors [8], [9], contribute to this protection variability. A key factor among the possible scenarios attributable to the vaccine protection variability is the genetic differences among BCG strains [2]. The immune system represented by T cells is essential for host recognition and control of *M. tuberculosis*, which depends on binding of the specific antigen epitopes [10], so mutations in the T cell antigen epitopes of BCG vaccine affect their degrees of protection. To obtain a more comprehensive understanding of the diversity of BCG strains and identify more candidate sites for vaccine development, we determined the genome sequence of six BCG strains in this study and used the genomic sequences of 19 *M. tuberculosis* complex (MTBC) strains to analyze their mutation sites (regions of deletion [RDs] and single nucleotide proteins [SNPs]), with special emphasis placed on 483 experimentally verified human T-cell epitopes [10].

## Methods

### BCG genome sequencing and assembly

Whole genome sequences of *M. bovis* (AF2122/97), seven strains of *M. bovis* BCG (BCG Mexico, BCG China, BCG Russia, BCG Tice, BCG Danish, Tokyo 172 and Pasteur) and five strains of *M. tuberculosis* (H37Rv, H37Ra, F11, KZN1435 and CDC1551) were downloaded from the NCBI database [3], [10], [11], [12], [13], [14], [15], [16], [17], [18]. Detailed information about these strains is listed in Table S2.

All strains of *M. bovis* BCG used in this study were provided by American Type Culture Collection (USA). We sequenced the genome of six BCG strains (BCG-Frappier, BCG-Glaxo, BCG-Moreau, BCG-Phipps, BCG-Pragure and BCG-Sweden) using an illumine genome analyzer. The genome coverage was >100-fold. Genomic DNA was extracted from BCG colonies on L-J medium using CTAB, and 2 µg of DNA from each strain was used for the sequencing. Sequencing reads from the six BCG strains were assembled into draft genomes using SOAPdenovo (BGI) (Table S2) [19].

### RD, Absence genes, lost epitopes and SNP identification

The 3945 coding DNA sequences from *M. bovis* AF2122/97 were compared individually with the other 18 MTBC strains (Table S2) using BLAST [20] for identifying presence/absence (PA) genes. Absence genes were defined as sequence alignment <60%. All identified absence genes were also checked by alignment with the original sequencing reads using SOAP [17], and some negative absence genes caused by assembly errors were filtered out.

To further filter out false-negative absence genes in the draft genomes, only those absence genes that were not located at the ends of a contig were considered absence genes. RDs covering one or more absence genes were identified based on the gene locations. The epitope was classified as a lost epitope if it was located in a absence gene or in a deleted region of a non-absence gene. Only those epitopes without BLAST matches in the genome were left as the lost epitopes in this strain.

To identify the SNPs, we first obtained 3,945 gene sequences for each of the 18 strains using the BLAST results and aligned them using ClustalW [21]. Only SNP sites with coverage >20 and without ambiguous sites (“N”) in their flanking 10-bp regions were kept.

### RD rate calculation

For RD rate calculation, we first obtained the RD number for each strain. The RD rate is the average RD number divided by the evolution time. The evolution time was presumed based on previous public records about BCG and *M. tuberculosis*. Based on the records, attenuation of BCG strains from *M. bovis* began in 1908 and was completed after 13 years (1921). In 1921–1966, the BCG strains diverged due to separate culturing in different countries/regions. Since 1966, BCG vaccines have not been prepared from cultures that have undergone more than 12 passages. In other words, since 1966, no new mutations occurring in the BCG strains would have been perpetuated. Thus, in theory, all BCG mutations, including both SNPs and RDs, occurred during 1908–1921 and 1921–1966. In addition, *M. tuberculosis* was estimated to have occurred roughly 15,000–20,000 years ago [22].

### Phylogenetic analysis

Phylogenetic analysis was first performed using SNPs from the concatenated sequences of 17 housekeeping genes (Table S1). A neighbor-joining (NJ) no-root tree was obtained using MEGA [23]. A further topological structure tree was based on all of the absence genes and was obtained using Cluster and TreeView [24].

## Results

### Hyperconserved BCG strains

**Figure 1:**
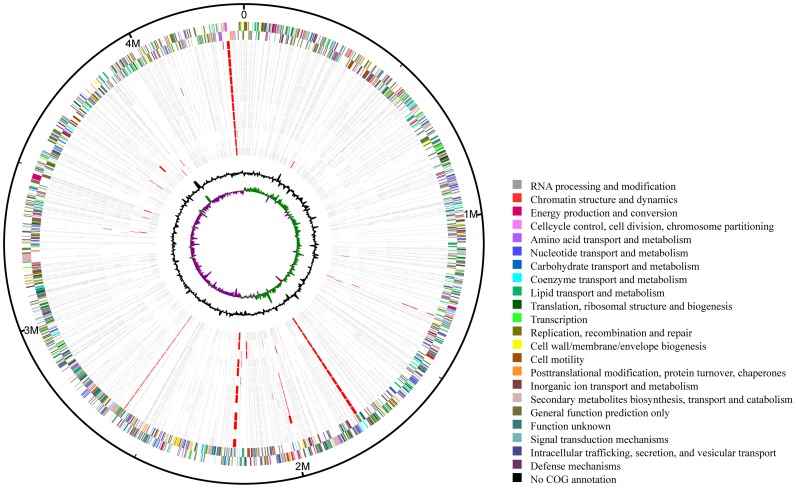
Distribution of single nucleotide proteins (SNPs) and regions of deletion (RDs) in *Mycobacterium bovis* AF2122/97 and 13 Bacillus Calmette-Guérin (BCG) strains. Outer circle: coding DNA sequences from the AF2122/97 genome are shown in a pair of concentric rings representing both coding strands; two inner circles: G+C% content and GC deviation (>0% green, <0% purple); other circles, from outer to inner: SNPs (grey) and RDs (red) between AF2122/97 and 13 BCG strains (Mexico, Frappier, Glaxo, Moreau, Phipps, Prague, Sweden, China, Danish, Russia, Tice, Pasteur and Tokyo).

**Figure 2:**
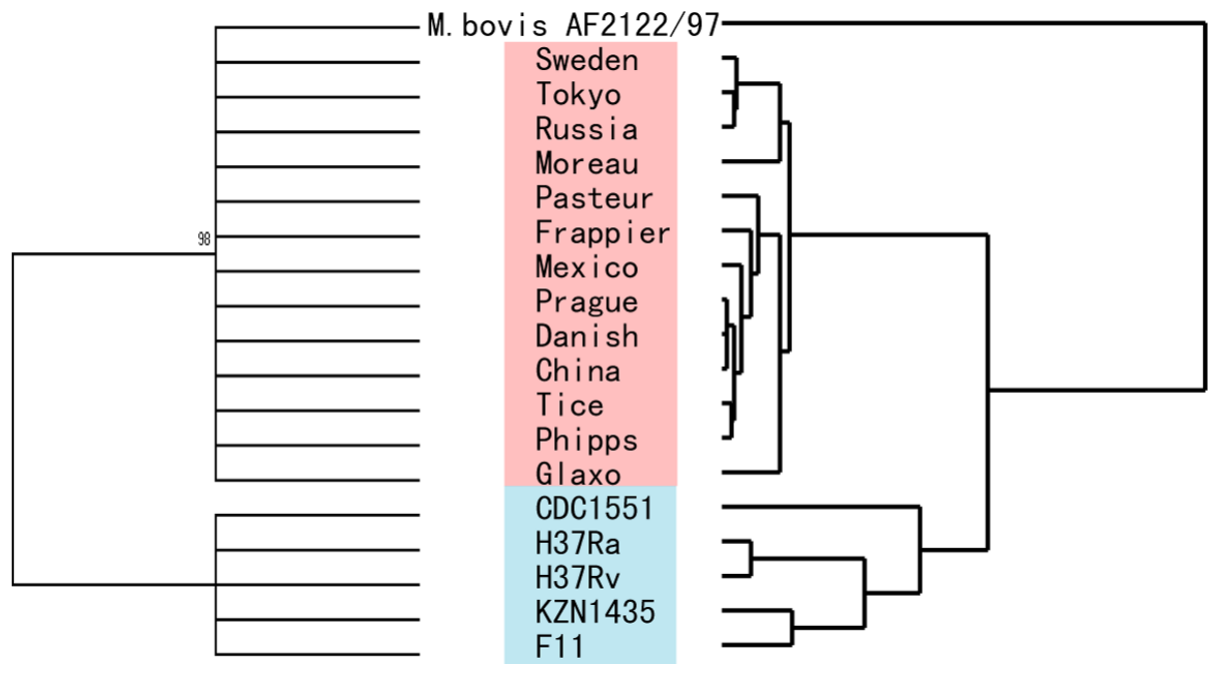
Phylogenetic trees of *Mycobacterium bovis* Bacullus Calmette-Guérin (BCG), *M. bovis* and *Mycobacterium tuberculosis* strains. (a) Neighbor-joining tree based on 17 housekeeping genes. The tree was rooted using *M. bovis* AF2122/97. (b) Topological structure tree based on absence genes. Strains of *M. bovis* BCG are shown in red while strains of *M. tuberculosis* are shown in blue.

We compared the genomes of 13 BCG strains (BCG-Frappier, BCG-Glaxo, BCG-Moreau, BCG-Phipps, BCG-Pragure, BCG-Sweden, BCG-China, BCG-Tice, BCG-Russia, BCG-Danish, BCG-Mexico, BCG-Tokyo and BCG-Pasteur) and five *M. tuberculosis* strains (F11, H37Ra, H37Rv, KZN1435 and CDC1511) (Figure 1). We first determined the evolutionary relationship among these MTBC strains by constructing an NJ phylogenetic tree based on 17 housekeeping genes (Figure 2a and Table S1). Like their common ancestor *M. bovis* AF2122/97 and the 5 *M. tuberculosis* stains, all of the BCG strains had similar genome sizes (∼4.2 M) and GC contents (∼0.65) (Table S2). The repetitive BCG regions are listed in Table S9.

We identified a total of 2,235 SNPs in the 13 BCG strains and 1444 SNPs in the 5 *M. tuberculosis* strains. The average nucleotide diversity for pairs of any two BCG strains was only 0.018 SNP/kb, significantly lower (*P* = 4.43e–6<0.01, two-tailed *t-*test) than those of the *M. tuberculosis* strains (0.25 SNP/kb). These identified SNPs could be used as new molecular marks for BCG strain identification in the future (Table S3).

### Conserved but high RD rate of BCG genomes

**Figure 3:**
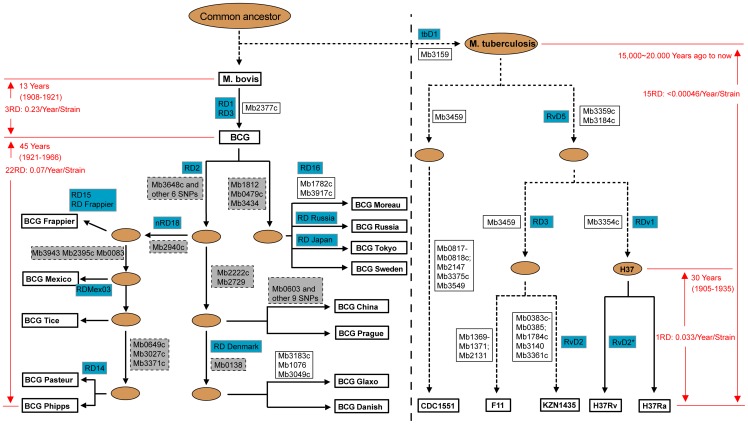
Genealogy of Bacillus Calmete-Guérin (BCG) vaccine strains and *Mycobacterium tuberculosis* strains. The genealogy of BCG strains based on Keller et al. ^15^, displays a series of genomic alterations including regions of deletion (RDs; squares bordered with a solid line) and single nucleotide proteins (grey squares bordered with a dotted line). The blue squares in the figure represent published RDs, while the white squares represent newly identified RDs. The brown ovals are assumed to be ancestor strains without genome sequences. The solid arrows represent the process of strains living in the lab under human manipulation conditions, while the dotted arrows represent the process of strains living in their natural environments. The BCG and *M. tuberculosis* strains are divided by dotted lines in the figure.

In this study, we also identified 25 RDs (11 previously published [3], [18], [22], [25], [26], [27], [28] and 14 new; Table S4) that cover one or more absence genes in the genome sequences of these BCG strains. The topological structure tree based on RDs (Figure 2b) more clearly shows the relationship among the *M. bovis* BCG and *M. tuberculosis* strains than does the NJ phylogenetic tree (Figure 2a). With the well-documented history of BCG vaccines, this enables us to accurately predict the time that most of these RDs occurred (Figure 3). RD1, RD3 and Del_Mb2377c most likely occurred during the first attenuation period (1908–1921) [2], while the remaining 22 RDs, which contain a total of 52 absence genes, probably occurred during the following period of divergence (1921–1966).

RDs also occurred in *M. tuberculosis* strains. A total of 17 RDs covering 44 genes were found in the five strains of *M. tuberculosis* examined here compared to the *M. bovis* genome (Figure 3), and all are potential molecular markers for distinguishing *Mycobacterium* sp.

We compared the RD occurrence rate in BCG and *M. tuberculosis* and found that BCG has a markedly higher RD rate (Figure 3). In theory, all mutations in BCG, including both SNPs and RDs, occurred during 1908–1921 or 1921–1966. Thus, the average RD rate of each BCG strain during these two periods is 0.23/year/strain and 0.07/year/strain, respectively (Figure 3). For *M. tuberculosis*, the average RD rate for each strain would be 0.00035–0.00046/year/strain, less than that of BCG (0.07/year/strain). Unlike other clinical *M. tuberculosis* strains, H37Ra and H37Rv are both derived from their virulent parent strain H37 through a process of aging and dissociation from in vitro culture between 1905 and 1935 [12]. The genome comparison in this study represented that the RD rate during this period for these two strains under human manipulation (0.033 RD/year/strain) is clearly higher than those of other *M. tuberculosis* strains surviving in natural environments (<0.00046 RD/year/strain; Figure 3) but is still lower than that of BCG strains (0.07/year/strain). Thus, it is reasonable to speculate that BCG has a significantly higher RD rate than *M. tuberculosis*.

### Loss of T-cell epitopes of the BCG genome

**Figure 4:**
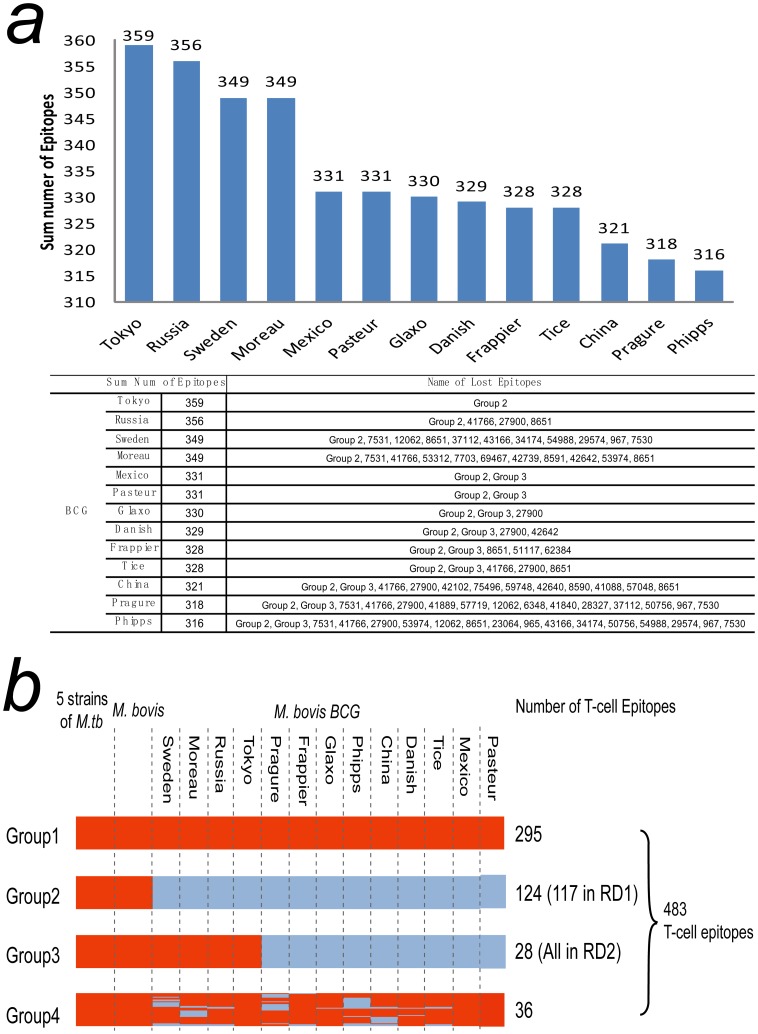
Distribution of the 483 T-cell epitopes in the 19 *Mycobacterium tuberculosis* c complex (MTBC) strains. (a) Total number of the epitopes in each of the 13 Bacillus Calamette-Guérin strains. The table lists the names of the epitopes that are absent from each strain. (b) Four groups of T-cell epitopes in the 19 MTBC strains. Epitopes shown in red are present, while those shown in sky blue are absent.

Our research of 483 experimentally verified human T-cell epitopes (Table S5) [10] indicated that several T-cell epitopes have been lost in BCG strains, although all of them exist in *M. bovis* and five strains of *M. tuberculosis* (Figure 4). Only 295 T-cell epitopes (Tables S5& S6) are presented in all 13 BCG strains and classified as Group 1 Epitopes (Figure 4). Our results showed that epitope sequences in *M. tuberculosis* and BCG are both highly conserved. Of the 483 experimentally-verified human T-cell epitopes (Table S5) [10], only eight SNPs were identified in the genomes of the five *M. tuberculosis* strains, consistent with the previously reported observation of the highly conserved epitopes in *M. tuberculosis* genomes [10]. The BCG T-cell epitopes were also conserved, for no SNP were ever identified in these 295 T-cell epitopes of the 13 examined BCG strains.

In addition to the 295 T-cell epitopes in Group 1, the other 188 T-cell epitopes in the BCG strains were lost to varying degrees. The first BCG epitope loss occurred during the attenuation period (1908–1921). As shown in Figure 4b, 124 T-cell epitopes classified as Group 2 were lost in all BCG strains. Most (117; 94.4%) of Group 2 T-cell epitopes are located within RD1, which encodes several essential antigens (Table S7) such as immunogenic co-regulated secreted proteins (ESAT-6 and CFP-10). Between 1926 (the dissemination time of BCG-Sweden) and 1934 (the dissemination time of BCG-Tice) [2], the other 28 T-cell epitopes, all of which are located in RD2 and classified as Group 3, were lost during the ongoing propagation of eight BCG strains, while the others (BCG-Moreau, BCG-Russia, BCG-Tokyo and BCG-Sweden) retained these Group 3 epitopes (Figure 4b). Thus, compared to other BCG strains, these four strains contain more antigens such as MPT64 (Table S7) that are recognized by the immune system [29]. Each BCG has unique lost epitopes (Figures 4a and 4b) that are classified into Group 4. Of the 13 BCG strains examined here, the BCG Tokyo strain had the highest number of epitopes (359) (Figure 4a).

## Discussion

### Effect of human manipulation on the evolution of BCG

While *M. bovis* BCG, which is derived from *M. bovis*, and *M. tuberculosis* originated from a common ancestor [22], they have existed in different environments and experienced different selection pressures since their segregation. BCG strains have been grown under artificial culture in labs around the world and have always been subject to human manipulation, while *M. tuberculosis* strains, except for the laboratory strains H37Ra and H37Rv, must survive in their natural environments and are subject to the selection pressure of the human immune system. Different mutation models have arisen in BCG and *M. tuberculosis* as a result of these different environments and selection pressures since their segregation.

Although RDs have occurred over the evolutionary course of the *M. tuberculosis* and BCG vaccines, they have been especially frequent in BCG. Comparison of the RD rate in BCG and *M. tuberculosis* shows that RDs occur and are more frequently maintained in BCG than that in *M. tuberculosis* (Figure 3). Because they existed in different environments and experienced different selection pressures, we hypothesize that human-manipulated BCG vaccine strains are under greater selection pressure to tolerate more RDs than natural *M. tuberculosis*. In other words, the manipulation of strains under laboratory conditions compared to natural hosts tends to more readily result in sequence loss. The relative higher RD rate between laboratory strains H37Rv and H37Ra under human manipulation (0.033 RD/year/strain) than those in other *M. tuberculosis* strains surviving in natural environments (<0.00046 RD/year/strain; Figure 3) further supports our hypothesis that human manipulation of bacterial strains results in strong positive selection of RDs.

These RDs identified in several BCG strains could also explain their immunological efficiency and why differences in virulence levels remain in BCG descendant strains. It may be possible to leverage these differences and manually delete or insert particular regions in BCG to develop new BCG strains with better immunological efficiency. For example, restoration of the RD1 region into BCG has been proven to improve its vaccine efficacy [30].

### BCG PA T-cell epitopes can be used to develop new vaccines

T-cell antigens consist of epitope regions of pathogens that interact with human T cells and are recognized by the immune system. Studies in pathogenic viruses, bacteria and protozoa have revealed that genes encoding antigens and their T-cell epitope regions tend to be highly variable as a consequence of diversifying selection to evade host immunity [10], [31], [32], [33], [34]. However, Comas et al. reported the 491 experimentally confirmed human T-cell epitopes are even more conserved than several essential genes [10]. Our results indicate that epitope sequences in BCG are conserved similar to those in *M. tuberculosis*. While we identified eight SNPs among 483 of the 491 T-cell epitopes examined by Comas et al [10] in five *M. tuberculosis* strains (sequence information was not provided for the remaining eight epitopes), no SNPs were identified in these T-cell epitopes in the 13 BCG strains examined here.

Although T-cell epitopes are essential for the immune system response and are highly conserved in BCG strains, our results indicate that several epitopes had been lost in BCG strains. The 483 T-cell epitopes (Figures 4a and 4b) can be divided into four groups based on their distribution among the 19 MTBC strains. While 295 T-cell epitopes were present in all 19 MTBC strains (Group 1), the 188 epitopes of Groups 2, 3 and 4 were lost in some or all of the BCG strains (Figure 4b). This finding may provide insight into differences in the protective capacity of BCG strains [5], [6], [7] and could be useful for the development of new DNA, epitope or recombination TB vaccines. Of the 188 epitopes lost in some or all BCG strains, 124 T-cell epitopes (Group 2) are absent from all BCG strains but have been maintained in the five strains of *M. tuberculosis*, and most (117; 94.4%) are located in RD1 (Figure 4b). RD1 encodes a pair of highly immunogenic co-regulated secreted proteins (ESAT-6 and CFP-10) that contain T- and B-cell epitopes. In 2003, Pym found that the restoration of ESAT-6 in BCG improves its vaccine efficacy [30]. Likewise, the 188 epitopes identified here could also be candidates for restoration into BCG to improve its vaccine efficacy.

BCG strains have strain-specific RDs. The strains most commonly in use, such as BCG Glaxo, Danish and Pasteur, have the largest number of RDs. It has been proposed that one of the reasons behind the partial BCG vaccine efficacy is that it has become too attenuated to successfully mimic natural MTB infection [35]. Some empirical evidence favoring this hypothesis is provided by the finding that the BCG Japan strain induced greater cytotoxicity and T helper 1 responses in infants than the BCG Danish strain [36]. The BCG Japan strain was also proven to induce higher frequencies of mycobacterial-specific polyfunctional and cytotoxic T cells and higher concentrations of Th1 cytokines than that of the BCG Russia strain [37]. Our results showed that the 13 BCG strains have different numbers of T-cell epitopes. The BCG Tokyo strain, which is non-virulent and lost only Group 2 epitopes (Figure 4a), has the largest number of T-cell epitopes that can be recognized by the immune system. It might be the only strain with the same number of epitopes as the first BCG vaccine strain in 1921. We propose that BCG Tokyo is the best candidate strain for use in the development of a new and better vaccine.

## Conclusion

In summary, our results showed that 188 T-cell epitopes essential to the human immune system response had been lost in BCG strains to varying degrees. The higher RD rates in the human-manipulated BCG strains suggests that the vaccine strains that had undergone human manipulation were under a dramatically different selection model from the natural strains. Our results also suggest that BCG Tokyo, the strain with the highest number of T-cell epitopes, may be the best candidate strain for the development of a better vaccine strain. Deletion or insertion of the epitopes identified here that are present in *M. tuberculosis* but absent in some or all BCG strains may be a useful strategy for vaccine development.

## Data Access

This Whole Genome Shotgun project has been deposited at DDBJ/EMBL/GenBank under the accession number AKYQ00000000-AKYV00000000. The genome sequences of the six BCG strains can be accessed here: http://www.mtbgenetyping.org/mtbDB/data/download/BCG.rar.

## Supporting Information

Table S1 **Housekeeping genes examined in this study.**
(DOC)

Table S2 **Strains used in this paper.**
(DOC)

Table S3 **List of single nucleotide proteins in Bacillus Calamette-Guérin (BCG) that could be used as new molecular marks for BCG strain identification.**
(DOC)

Table S4 **Distribution of regions of deletion covering the absence genes in the 19** ***Mycobacterium tuberculosis*** **complex strains.** “+”: gene present; “−”: gene absent.
(DOC)

Table S5 **The 483 T-cell epitopes in the 13 BCG strains.** “+”: presence of the epitope; “−”: absence of the epitope.
(DOC)

Table S6 **Genes with Group 1 epitopes in H37Rv.** “+”: epitope in this gene exists in this BCG strain; “×”: epitopes in this gene does not exist in this BCG strain. “+/×”: there are several epitopes in this gene, and some were lost in this strain. “NA” in column 4 denotes that there are no related references to support that this gene is the antigen.
(DOC)

Table S7 **Genes with Group 2–4 epitopes in H37Rv.** “+”: epitopes in this gene exist in this BCG strain; “−”: epitopes in this gene do not exist in this BCG strain. “+/−”: there are several epitopes in this gene, and some were lost in this strain. “NA” in column 4 denotes that there are no related references to support that this gene is the antigen.
(DOC)

Table S8 **Presence/absence genes in 16 strains of** ***Mycobacterium tuberculosis*** **(CDC1551, H37Rv, H37Ra, RGTB423, 7199-99, ATCC35801, NITR203, RGTB327, F11, UT205, CCDC5079, KZN1435, KZN4207, KZN605 and CTRI-2).** Whole genome sequences of these strains were downloaded from the NCBI database. “+”: this gene exists; “−”: this gene was lost.
(DOC)

Table S9 **The presense of repetitive sequences in the 13 Bacillus Calmette-Guérin strains.**
(DOC)
