# Supplementary material for: Genome Sequencing and Analysis of BCG Vaccine Strains
Source: PLoS One. 2013 Aug 19;8(8):e71243. doi: 10.1371/journal.pone.0071243 (PMC3747166; doi:10.1371/journal.pone.0071243)
Supplement: Table S1 — Housekeeping genes examined in this study. (DOC) [file pone.0071243.s001.doc]

**Table S1**: Housekeeping genes examined in this study[^1^](#_ENREF_1)

| **Name** | **Name** |
| --- | --- |
| ***rmlC*** | *rplM* |
| ***mpt53*** | *rplA* |
| ***panD*** | *rplB* |
| ***gcvH*** | *rplC* |
| ***recX*** | *rplD* |
| ***mprA*** | *rplE* |
| ***ideR*** | *rplF* |
|  | *rplJ* |
|  | *rplN* |
|  | *rplP* |
