# Supplementary material for: Genome Sequencing and Analysis of BCG Vaccine Strains
Source: PLoS One. 2013 Aug 19;8(8):e71243. doi: 10.1371/journal.pone.0071243 (PMC3747166; doi:10.1371/journal.pone.0071243)
Supplement: Table S2 — Strains used in this paper. (DOC) [file pone.0071243.s002.doc]

**Table S2**: Strains used in this paper

| **Strain** | | **Size (Mbp)** | **GC** | **Status** | **Accession** | **Reference** |
| --- | --- | --- | --- | --- | --- | --- |
| ***Mycobacterium bovis*** | AF2122/97 | 4.35 | 0.656 | complete | NC_002945 |  |
| ***Mycobacterium bovis* BCG** | Tokyo 172 | 4.37 | 0.656 | complete | NC_012207 |  |
|  | Pasteur 1173P2 | 4.37 | 0.656 | complete | NC_008769 |  |
|  | Mexico | 4.35 | 0.657 | complete | NC_016804 |  |
|  | China | 4.18 | 0.653 | Draft | AEZE00000000 |  |
|  | Danish | 4.17 | 0.653 | Draft | AEZF00000000 |  |
|  | Russia | 4.18 | 0.653 | Draft | AEZG00000000 |  |
|  | Tice | 4.17 | 0.653 | Draft | AEZH00000000 |  |
|  | Frappier | 4.24 | 0.655 | Draft | AKYQ00000000 |  |
|  | Glaxo | 4.21 | 0.654 | Draft | AKYR00000000 |  |
|  | Moreau | 4.16 | 0.654 | Draft | AKYS00000000 |  |
|  | Phipps | 4.14 | 0.654 | Draft | AKYT00000000 |  |
|  | Prague | 4.13 | 0.653 | Draft | AKYU00000000 |  |
|  | Sweden | 4.09 | 0.653 | Draft | AKYV00000000 |  |
| ***Mycobacterium tuberculosis*** | H37Rv | 4.41 | 0.656 | complete | NC_000962 |  |
|  | CDC1551 | 4.40 | 0.656 | complete | NC_002755 |  |
|  | F11 | 4.42 | 0.656 | complete | NC_009565 | Broad Institute |
|  | H37Ra | 4.42 | 0.656 | complete | NC_009525 |  |
|  | KZN1435 | 4.40 | 0.656 | complete | CP001658 | Broad Institute |

Reference

1. Garnier T, Eiglmeier K, Camus JC, Medina N, Mansoor H, et al. (2003) The complete genome sequence of Mycobacterium bovis. Proc Natl Acad Sci USA 100: 7877 - 7882.

2. Seki M, Honda I, Fujita I, Yano I, Yamamoto S, et al. (2009) Whole genome sequence analysis of Mycobacterium bovis bacillus Calmette-Guerin (BCG) Tokyo 172: A comparative study of BCG vaccine substrains. Vaccine 27: 1710-1716.

3. Comas I, Chakravartti J, Small PM, Galagan J, Niemann S, et al. (2010) Human T cell epitopes of Mycobacterium tuberculosis are evolutionarily hyperconserved. Nat Genet 42: 498-503.

4. Orduña P, Cevallos M, de León S, Arvizu A, Hernández-González I, et al. (2011) Genomic and proteomic analyses of Mycobacterium bovis BCG Mexico 1931 reveal a diverse immunogenic repertoire against tuberculosis infection. BMC Genomics 12: 493.

5. Pan Y, Yang X, Duan J, Lu N, Leung AS, et al. (2011) The whole genome sequence of four BCG vaccine strains. Journal of Bacteriology.

6. Cole ST, Brosch R, Parkhill J, Garnier T, Churcher C, et al. (1998) Deciphering the biology of Mycobacterium tuberculosis from the complete genome sequence. Nature 393: 537 - 544.

7. Camus J-C, Pryor MJ, Medigue C, Cole ST (2002) Re-annotation of the genome sequence of Mycobacterium tuberculosis H37Rv. Microbiology 148: 2967-2973.

8. Fleischmann RD, Alland D, Eisen JA, Carpenter L, White O, et al. (2002) Whole-Genome Comparison of Mycobacterium tuberculosis Clinical and Laboratory Strains. J Bacteriol 184: 5479-5490.

9. Zheng H, Lu L, Wang B, Pu S, Zhang X, et al. (2008) Genetic Basis of Virulence Attenuation Revealed by Comparative Genomic Analysis of Mycobacterium tuberculosis Strain H37Ra versus H37Rv. PLoS ONE 3: e2375.
