# Supplementary material for: Genome Sequencing and Analysis of BCG Vaccine Strains
Source: PLoS One. 2013 Aug 19;8(8):e71243. doi: 10.1371/journal.pone.0071243 (PMC3747166; doi:10.1371/journal.pone.0071243)
Supplement: Table S3 — List of single nucleotide proteins in Bacillus Calamette-Guérin (BCG) that could be used as new molecular marks for BCG strain identification. (DOC) [file pone.0071243.s003.doc]

**Table S3.** List of SNPs in BCG which could be used as the new molecular mark for the BCG strain identification.

| **Gene** | **Site in gene** | **Alleles(Ref/Mutation)** | **Strains with Mutations** |
| --- | --- | --- | --- |
| *Mb0083* | 102 | C/T | BCG_Tice; BCG_Phipps; BCG_Pasteur |
| *Mb2395c* | 796 | C/T | BCG_Tice; BCG_Phipps; BCG_Pasteur |
| *Mb3371c* | 637 | G/A | BCG_Tice; BCG_Phipps; BCG_Pasteur |
| *Mb3943* | 278 | C/T | BCG_Tice; BCG_Phipps; BCG_Pasteur |
| *Mb0037c* | 217 | G/A | BCG_Phipps; BCG_Pasteur |
| *Mb0649c* | 146 | C/T | BCG_Phipps; BCG_Pasteur |
| *Mb3027c* | 358 | G/A | BCG_Phipps; BCG_Pasteur |
| *Mb0479c* | 404 | T/G | BCG_Moreau; BCG_Russia; BCG_Tokyo; BCG_Sweden |
| *Mb1812* | 1940 | G/A | BCG_Moreau; BCG_Russia; BCG_Tokyo; BCG_Sweden |
| *Mb3434* | 2248 | T/G | BCG_Moreau; BCG_Russia; BCG_Tokyo; BCG_Sweden |
| *Mb0138* | 62 | C/T | BCG_Glaxo; BCG_Danish |
| *Mb2573* | 21 | C/T | BCG_Glaxo; BCG_Danish |
| *Mb2573* | 35 | A/G | BCG_Glaxo; BCG_Danish |
| *Mb2573* | 223 | G/A | BCG_Glaxo; BCG_Danish |
| *Mb0453c* | 561 | C/T | BCG_Frappier; BCG_Tice; BCG_Phipps; BCG_Pasteur; BCG_China; BCG_Prague; BCG_Danish; BCG_Glaxo |
| *Mb0567* | 430 | T/C | BCG_Frappier; BCG_Tice; BCG_Phipps; BCG_Pasteur; BCG_China; BCG_Prague; BCG_Danish; BCG_Glaxo |
| *Mb0662c* | 589 | C/T | BCG_Frappier; BCG_Tice; BCG_Phipps; BCG_Pasteur; BCG_China; BCG_Prague; BCG_Danish; BCG_Glaxo |
| *Mb1812* | 2074 | C/T | BCG_Frappier; BCG_Tice; BCG_Phipps; BCG_Pasteur; BCG_China; BCG_Prague; BCG_Danish; BCG_Glaxo |
| *Mb3159* | 636 | G/A | BCG_Frappier; BCG_Tice; BCG_Phipps; BCG_Pasteur; BCG_China; BCG_Prague; BCG_Danish; BCG_Glaxo |
| *Mb3648c* | 287 | C/T | BCG_Frappier; BCG_Tice; BCG_Phipps; BCG_Pasteur; BCG_China; BCG_Prague; BCG_Danish; BCG_Glaxo |
| *Mb3700* | 139 | T/C | BCG_Frappier; BCG_Tice; BCG_Phipps; BCG_Pasteur; BCG_China; BCG_Prague; BCG_Danish; BCG_Glaxo |
| *Mb2940c* | 1179 | C/T | BCG_Frappier; BCG_Tice; BCG_Phipps; BCG_Pasteur |
| *Mb2222c* | 351 | G/A | BCG_China; BCG_Prague; BCG_Danish; BCG_Glaxo |
| *Mb2729* | 136 | T/C | BCG_China; BCG_Prague; BCG_Danish; BCG_Glaxo |
| *Mb0514* | 579 | C/T | BCG_China |
| *Mb0603* | 872 | C/A | BCG_China |
| *Mb0780* | 363 | C/T | BCG_China |
| *Mb1190* | 232 | C/T | BCG_China |
| *Mb1411* | 1404 | C/G | BCG_China |
| *Mb1722* | 1305 | G/A | BCG_China |
| *Mb2421c* | 440 | C/T | BCG_China |
| *Mb3159* | 659 | C/A | BCG_China |
| *Mb3648c* | 421 | G/A | BCG_China |
| *Mb3750* | 537 | A/G | BCG_China |
