# Supplementary material for: Genome Sequencing and Analysis of BCG Vaccine Strains
Source: PLoS One. 2013 Aug 19;8(8):e71243. doi: 10.1371/journal.pone.0071243 (PMC3747166; doi:10.1371/journal.pone.0071243)
Supplement: Table S4 — Distribution of regions of deletion covering the absence genes in the 19 Mycobacterium tuberculosis complex strains. “+”: gene present; “−”: gene absent. (DOC) [file pone.0071243.s004.doc]

**Table S4**: Distribution of RDs which covering Absence genes in the 19 MTBC strains

“+”: gene is present in this strain; “-”: gene is absent in this strain.

| Gene Name |  | *M. bovis* AF2122 | *M. bovis BCG* | | | | | | | | | | | | | *M. tuberculosis* | | | | |
| --- | --- | --- | --- | --- | --- | --- | --- | --- | --- | --- | --- | --- | --- | --- | --- | --- | --- | --- | --- | --- |
|  | RD type |  | Mexico | Frappier | Glaxo | Moreau | Phipps | Prague | Sweden | China | Danish | Russia | Tice | Pasteur | Tokyo | H37Rv | H37Ra | F11 | KZN1435 | CDC1551 |
| Mb0317 | RD15 | + | + | - | + | + | + | + | + | + | + | + | + | + | + | + | + | + | + | + |
| Mb0318c | RD15 | + | + | - | + | + | + | + | + | + | + | + | + | + | + | + | + | + | + | + |
| Mb0319 | RD15 | + | + | - | + | + | + | + | + | + | + | + | + | + | + | + | + | + | + | + |
| Mb0320 | RD15 | + | + | - | + | + | + | + | + | + | + | + | + | + | + | + | + | + | + | + |
| Mb0383c | New1 | + | + | + | + | + | + | + | + | + | + | + | + | + | + | + | + | + | - | + |
| Mb0384 | New1 | + | + | + | + | + | + | + | + | + | + | + | + | + | + | + | + | + | - | + |
| Mb0385 | New1 | + | + | + | + | + | + | + | + | + | + | + | + | + | + | + | + | + | - | + |
| Mb0817 | New2 | + | + | + | + | + | + | + | + | + | + | + | + | + | + | + | + | + | + | - |
| Mb0818c | New2 | + | + | + | + | + | + | + | + | + | + | + | + | + | + | + | + | + | + | - |
| Mb1076 | New3 | + | + | + | - | + | + | + | + | + | + | + | + | + | + | + | + | + | + | + |
| Mb1222 | nRD18 | + | - | - | + | + | - | + | + | + | + | + | - | - | + | + | + | + | + | + |
| Mb1223 | nRD18 | + | - | - | + | + | - | + | + | + | + | + | - | - | + | + | + | + | + | + |
| Mb1231c | New4 | + | + | + | - | + | + | + | + | + | + | + | + | + | + | + | + | + | + | + |
| Mb1369 | New5 | + | + | + | + | + | + | + | + | + | + | + | + | + | + | + | + | - | + | + |
| Mb1370 | New5 | + | + | + | + | + | + | + | + | + | + | + | + | + | + | + | + | - | + | + |
| Mb1371 | New5 | + | + | + | + | + | + | + | + | + | + | + | + | + | + | + | + | - | + | + |
| Mb1582 | TbD1 | + | + | + | + | + | + | + | + | + | + | + | + | + | + | - | - | - | - | - |
| Mb1583 | TbD1 | + | + | + | + | + | + | + | + | + | + | + | + | + | + | - | - | - | - | - |
| Mb1598A | New6 | + | + | + | + | + | + | + | + | + | + | + | + | + | + | + | + | - | - | + |
| Mb1599 | RD3 | + | - | - | - | - | - | - | - | - | - | - | - | - | - | + | + | - | - | + |
| Mb1600 | RD3 | + | - | - | - | - | - | - | - | - | - | - | - | - | - | + | + | - | - | + |
| Mb1601 | RD3 | + | - | - | - | - | - | - | - | - | - | - | - | - | - | + | + | - | - | + |
| Mb1602c | RD3 | + | - | - | - | - | - | - | - | - | - | - | - | - | - | + | + | - | - | + |
| Mb1603c | RD3 | + | - | - | - | - | - | - | - | - | - | - | - | - | - | + | + | + | + | + |
| Mb1604c | RD3 | + | - | - | - | - | - | - | - | - | - | - | - | - | - | + | + | - | - | + |
| Mb1605c | RD3 | + | - | - | - | - | - | - | - | - | - | - | - | - | - | + | + | - | - | + |
| Mb1606c | RD3 | + | - | - | - | - | - | - | - | - | - | - | - | - | - | + | + | - | - | + |
| Mb1607c | RD3 | + | - | - | - | - | - | - | - | - | - | - | - | - | - | + | + | - | - | + |
| Mb1608c | RD3 | + | - | - | - | - | - | - | - | - | - | - | - | - | - | + | + | - | - | + |
| Mb1609c | RD3 | + | - | - | - | - | - | - | - | - | - | - | - | - | - | + | + | - | - | + |
| Mb1610c | RD3 | + | - | - | - | - | - | - | - | - | - | - | - | - | - | + | + | - | - | + |
| Mb1611c | RD3 | + | - | - | - | - | - | - | - | - | - | - | - | - | - | + | + | - | - | + |
| Mb1612c | RD3 | + | - | - | - | - | - | - | - | - | - | - | - | - | - | + | + | - | - | + |
| Mb1782c | New7 | + | + | + | + | - | + | + | + | + | + | + | + | + | + | + | + | + | + | + |
| Mb1784c | RvD2 | + | + | + | + | + | + | + | + | + | + | + | + | + | + | + | + | + | - | + |
| Mb1785c | RvD2 | + | + | + | + | + | + | + | + | + | + | + | + | + | + | - | + | + | - | + |
| Mb1786 | RvD2 | + | + | + | + | + | + | + | + | + | + | + | + | + | + | - | + | + | - | + |
| Mb1787 | RvD2 | + | + | + | + | + | + | + | + | + | + | + | + | + | + | - | + | + | - | + |
| Mb1795A | RD14 | + | + | + | + | + | + | + | + | + | + | + | + | - | + | + | + | + | + | + |
| Mb1795c | RD14 | + | + | + | + | + | + | + | + | + | + | + | + | - | + | + | + | + | + | + |
| Mb1796 | RD14 | + | + | + | + | + | + | + | + | + | + | + | + | - | + | + | + | + | + | + |
| Mb1797 | RD14 | + | - | + | + | + | + | + | - | - | - | - | - | - | + | + | + | + | + | + |
| Mb1798 | RD14 | + | + | + | + | + | + | + | + | + | + | + | + | - | + | + | + | + | + | + |
| Mb1799 | RD14 | + | + | + | + | + | + | + | + | + | + | + | + | - | + | + | + | + | + | + |
| Mb1800 | RD14 | + | + | + | + | + | + | + | + | + | + | + | + | - | + | + | + | + | + | + |
| Mb1801 | RD14 | + | + | + | + | + | + | + | + | + | + | + | + | - | + | + | + | + | + | + |
| Mb1840 | RDDenmark/Glaxo | + | + | + | - | + | + | + | + | + | - | + | + | + | + | + | + | + | + | + |
| Mb1951c | New8 | + | + | + | - | - | + | + | + | + | + | + | + | + | + | + | + | + | + | + |
| Mb1980 | New9 | + | + | + | + | - | - | + | - | + | + | + | + | + | + | + | + | + | + | + |
| Mb2000 | RD2 | + | - | - | - | + | - | - | + | - | - | + | - | - | + | + | + | + | + | + |
| Mb2001c | RD2 | + | - | - | - | + | - | - | + | - | - | + | - | - | + | + | + | + | + | + |
| Mb2002c | RD2 | + | - | - | - | + | - | - | + | - | - | + | - | - | + | + | + | + | + | + |
| Mb2003c | RD2 | + | - | - | - | + | - | - | + | - | - | + | - | - | + | + | + | + | + | + |
| Mb2004c | RD2 | + | - | - | - | + | - | - | + | - | - | + | - | - | + | + | + | + | + | + |
| Mb2005 | RD2 | + | - | - | - | + | - | - | + | - | - | + | - | - | + | + | + | + | + | + |
| Mb2006c | RD2 | + | - | - | - | + | - | - | + | - | - | + | - | - | + | + | + | + | + | + |
| Mb2007c | RD2 | + | - | - | - | + | - | - | + | - | - | + | - | - | + | + | + | + | + | + |
| Mb2008 | RD2 | + | - | - | - | + | - | - | + | - | - | + | - | - | + | + | + | + | + | + |
| Mb2009 | RD2 | + | - | - | - | + | - | - | + | - | - | + | - | - | + | + | + | + | + | + |
| Mb2048c | RvD1 | + | + | + | + | + | + | + | + | + | + | + | + | + | + | - | - | + | + | + |
| Mb2049c | RvD1 | + | + | + | + | + | + | + | + | + | + | + | + | + | + | - | - | + | + | + |
| Mb2131 | New10 | + | + | + | + | + | + | + | + | + | + | + | + | + | + | + | + | - | + | + |
| Mb2147 | New11 | + | + | + | + | + | + | + | + | + | + | + | + | + | + | + | + | + | + | - |
| Mb2377c | New12 | + | - | - | - | - | - | - | - | - | - | - | - | - | - | + | + | + | + | + |
| Mb3043c | New13 | + | + | + | + | - | + | + | - | + | + | + | + | + | + | + | + | + | + | + |
| Mb3047c | New14 | + | + | + | - | + | + | + | + | + | + | + | + | + | + | + | + | + | + | + |
| Mb3049c | New15 | + | + | + | - | + | + | + | + | + | + | + | + | + | + | + | + | + | + | + |
| Mb3140 | New16 | + | + | + | + | + | + | + | + | + | + | + | + | + | + | + | + | + | - | + |
| Mb3142 | New17 | + | + | + | - | + | + | + | + | + | + | + | + | + | + | + | + | + | + | + |
| Mb3159 | New18 | + | + | + | + | + | + | + | + | + | + | + | + | + | + | - | - | - | - | - |
| Mb3183c | New19 | + | + | + | - | + | + | + | + | + | + | + | + | + | + | + | + | + | + | + |
| Mb3184c | New20 | + | + | + | - | + | + | + | + | + | + | + | + | + | + | - | - | - | - | + |
| Mb3354c | RvD5 | + | + | + | + | + | + | + | + | + | + | + | + | + | + | - | - | + | + | + |
| Mb3355c | RvD5 | + | + | + | + | + | + | + | + | + | + | + | + | + | + | - | - | - | - | + |
| Mb3356 | RvD5 | + | + | + | + | + | + | + | + | + | + | + | + | + | + | - | - | - | - | + |
| Mb3357 | RvD5 | + | + | + | + | + | + | + | + | + | + | + | + | + | + | - | - | - | - | + |
| Mb3358 | RvD5 | + | + | + | + | + | + | + | + | + | + | + | + | + | + | - | - | - | - | + |
| Mb3359c | RvD5 | + | + | + | + | + | + | + | + | + | + | + | + | + | + | - | - | - | - | + |
| Mb3360 | New21 | + | + | + | - | - | + | + | + | + | + | + | + | + | + | + | + | + | + | + |
| Mb3361c | New22 | + | + | + | + | + | + | + | + | + | + | + | + | + | + | + | + | + | - | + |
| Mb3375c | New23 | + | + | + | + | + | + | + | + | + | + | + | + | + | + | + | + | + | + | - |
| Mb3433 | RD16 | + | + | + | + | - | + | + | + | + | + | + | + | + | + | + | + | + | + | + |
| Mb3434 | RD16 | + | + | + | + | - | + | + | + | + | + | + | + | + | + | + | + | + | + | + |
| Mb3436c | RD16 | + | + | + | + | - | + | + | + | + | + | + | + | + | + | + | + | + | + | + |
| Mb3437c | RD16 | + | + | + | + | - | + | + | + | + | + | + | + | + | + | + | + | + | + | + |
| Mb3438c | RD16 | + | + | + | + | - | + | + | + | + | + | + | + | + | + | + | + | + | + | + |
| Mb3439c | RD16 | + | + | + | + | - | + | + | + | + | + | + | + | + | + | + | + | + | + | + |
| Mb3459 | New24 | + | + | + | + | + | + | + | + | + | + | + | + | + | + | + | + | - | - | - |
| Mb3526c | RD Frappier | + | + | - | + | + | + | + | + | + | + | + | + | + | + | + | + | + | + | + |
| Mb3544c | New25 | + | + | + | - | + | + | + | + | + | + | + | + | + | + | + | + | + | + | + |
| Mb3549 | New26 | + | + | + | + | + | + | + | + | + | + | + | + | + | + | + | + | + | + | - |
| Mb3724 | RDRussia | + | + | + | + | + | + | + | + | + | + | - | + | + | + | + | + | + | + | + |
| Mb3890 | RDMex03 | + | - | + | + | + | + | + | + | + | + | + | + | + | + | + | + | + | + | + |
| Mb3891 | RDMex04 | + | - | + | + | + | + | + | + | + | + | + | + | + | + | + | + | + | + | + |
| Mb3892c | RDMex05 | + | - | + | + | + | + | + | + | + | + | + | + | + | + | + | + | + | + | + |
| Mb3902 | RD1 | + | - | - | - | - | - | - | - | - | - | - | - | - | - | + | + | + | + | + |
| Mb3903 | RD1 | + | - | - | - | - | - | - | - | - | - | - | - | - | - | + | + | + | + | + |
| Mb3904 | RD1 | + | - | - | - | - | - | - | - | - | - | - | - | - | - | + | + | + | + | + |
| Mb3905 | RD1 | + | - | - | - | - | - | - | - | - | - | - | - | - | - | + | + | + | + | + |
| Mb3906 | RD1 | + | - | - | - | - | - | - | - | - | - | - | - | - | - | + | + | + | + | + |
| Mb3907 | RD1 | + | - | - | - | - | - | - | - | - | - | - | - | - | - | + | + | + | + | + |
| Mb3908 | RD1 | + | - | - | - | - | - | - | - | - | - | - | - | - | - | + | + | + | + | + |
| Mb3909c | RD1 | + | - | - | - | - | - | - | - | - | - | - | - | - | - | + | + | + | + | + |
| Mb3917c | New27 | + | + | + | + | - | + | + | + | + | + | + | + | + | + | + | + | + | + | + |
