# Supplementary material for: Genome Sequencing and Analysis of BCG Vaccine Strains
Source: PLoS One. 2013 Aug 19;8(8):e71243. doi: 10.1371/journal.pone.0071243 (PMC3747166; doi:10.1371/journal.pone.0071243)
Supplement: Table S5 — The 483 T-cell epitopes in the 13 BCG strains. “+”: presence of the epitope; “−”: absence of the epitope. (DOC) [file pone.0071243.s005.doc]

**Table S5**. 483 T-cell epitopes in the 12 BCG strains. “+” represents that the epitope existing in this strain while “-” represents not.

| Epitope Name | Epitopes | | M. tb H37Rv | | | |  | BCG | | | | | | | | | | | |
| --- | --- | --- | --- | --- | --- | --- | --- | --- | --- | --- | --- | --- | --- | --- | --- | --- | --- | --- | --- |
|  | Epitopes Group | Protein sequence | Gene name | Start | End | Gene annotation | Prague | Sweden | Frappier | Glaxo | Moreau | Phipps | China | Danish | Russia | Tice | Mexico | Pasteur | Tokyo |
| 8300 | 1 | DFNRDSREVVHLATGMA | *Rv0200* | 237509 | 237559 | NA | + | + | + | + | + | + | + | + | + | + | + | + | + |
| 18914 | 1 | GCSSNKSTTGSGETTTAAGT | *Rv3763* | 4209107 | 4209166 | lpqH | + | + | + | + | + | + | + | + | + | + | + | + | + |
| 57256 | 1 | SDPALLAEIRQSLDATKGLTSVHVA | *Rv2945c* | 3291116 | 3291190 | lppx | + | + | + | + | + | + | + | + | + | + | + | + | + |
| 1674 | 1 | AGRVAQIRQEIENSD | *Rv0440* | 529628 | 529672 | groEL | + | + | + | + | + | + | + | + | + | + | + | + | + |
| 41161 | 1 | MATTLPVQRHPRSLFPEFSE | *Rv2031c* | 2278873 | 2278932 | hspx | + | + | + | + | + | + | + | + | + | + | + | + | + |
| 20707 | 1 | GKTYDAYFTDAGGITPG | *Rv3497c* | 3916777 | 3916827 | mce4C | + | + | + | + | + | + | + | + | + | + | + | + | + |
| 21960 | 1 | GQSSFYSDWY | *Rv1886c* | 2135499 | 2135528 | fbpB | + | + | + | + | + | + | + | + | + | + | + | + | + |
| 37930 | 1 | LLVSSKVSTVKDLLP | *Rv0440* | 529262 | 529306 | groEL | + | + | + | + | + | + | + | + | + | + | + | + | + |
| 5252 | 1 | AVAGAAILVAGLSGCSS | *Rv3763* | 4209068 | 4209118 | lpqH | + | + | + | + | + | + | + | + | + | + | + | + | + |
| 10838 | 1 | DWYQPACGKAGCQTYKWETF | *Rv3804c* | 4266230 | 4266289 | fbpA | + | + | + | + | + | + | + | + | + | + | + | + | + |
| 22252 | 1 | GRWDEDGEKRIPLDVA | *Rv3418c* | 3837097 | 3837144 | groES | + | + | + | + | + | + | + | + | + | + | + | + | + |
| 18276 | 1 | FVRSSNLKF | *Rv1886c* | 2135028 | 2135054 | fbpB | + | + | + | + | + | + | + | + | + | + | + | + | + |
| 14574 | 1 | ETTTASGLVIPDTAK | *Rv3418c* | 3837184 | 3837228 | groES | + | + | + | + | + | + | + | + | + | + | + | + | + |
| 70507 | 1 | VPWQPAFVF | *Rv2878c* | 3188972 | 3188998 | mpt53 | + | + | + | + | + | + | + | + | + | + | + | + | + |
| 5707 | 1 | AWYRLTKFFGKLFLINF | *Rv1623c* | 1825702 | 1825752 | cydA | + | + | + | + | + | + | + | + | + | + | + | + | + |
| 194 | 1 | AAHARFVAA | *Rv0287* | 351681 | 351707 | esxG | + | + | + | + | + | + | + | + | + | + | + | + | + |
| 5623 | 1 | AVYLLDGLR | *Rv1886c* | 2135619 | 2135645 | fbpB | + | + | + | + | + | + | + | + | + | + | + | + | + |
| 20662 | 1 | GKPTEKHIQIRSTN | *Rv2031c* | 2278501 | 2278542 | hspx | + | + | + | + | + | + | + | + | + | + | + | + | + |
| 8686 | 1 | DIKVQFQSGGANSPALYLLD | *Rv3804c* | 4266410 | 4266469 | fbpA | + | + | + | + | + | + | + | + | + | + | + | + | + |
| 34437 | 1 | KYGGTEIKYNGEEYLI | *Rv3418c* | 3837025 | 3837072 | groES | + | + | + | + | + | + | + | + | + | + | + | + | + |
| 25269 | 1 | IAFNSGLEPGVVAEK | *Rv0440* | 529958 | 530002 | groEL | + | + | + | + | + | + | + | + | + | + | + | + | + |
| 33707 | 1 | KTIAYDEEARR | *Rv0440* | 528614 | 528646 | groEL | + | + | + | + | + | + | + | + | + | + | + | + | + |
| 49280 | 1 | PRYISLIPVNVVAD | *Rv0589* | 687103 | 687144 | mce2A | + | + | + | + | + | + | + | + | + | + | + | + | + |
| 22882 | 1 | GTVVAVGPGRWDEDG | *Rv3418c* | 3837124 | 3837168 | groES | + | + | + | + | + | + | + | + | + | + | + | + | + |
| 35 | 1 | AAAGFASKTPANQAISMIDG | *Rv0934* | 1042964 | 1043023 | pstS1 | + | + | + | + | + | + | + | + | + | + | + | + | + |
| 28304 | 1 | IRGSVTPAVSQFNARTADGI | *Rv1926c* | 2179125 | 2179184 | mpt63 | + | + | + | + | + | + | + | + | + | + | + | + | + |
| 72294 | 1 | WDEDGEKRIPLDVAE | *Rv3418c* | 3837094 | 3837138 | groES | + | + | + | + | + | + | + | + | + | + | + | + | + |
| 73784 | 1 | YEVRAELPGVDPDKDVDIMV | *Rv2031c* | 2278723 | 2278782 | hspx | + | + | + | + | + | + | + | + | + | + | + | + | + |
| 54516 | 1 | RLEDEMKEGRYEVRAELPGV | *Rv2031c* | 2278753 | 2278812 | hspx | + | + | + | + | + | + | + | + | + | + | + | + | + |
| 16333 | 1 | FIYAGSLSAL | *Rv1886c* | 2135292 | 2135321 | fbpB | + | + | + | + | + | + | + | + | + | + | + | + | + |
| 63960 | 1 | TGSGAGIAQAAAGTVNI | *Rv0934* | 1042370 | 1042420 | pstS1 | + | + | + | + | + | + | + | + | + | + | + | + | + |
| 58475 | 1 | SIDLGSGSIQLTQSKWNEPVNVD | *Rv2945c* | 3290627 | 3290695 | lppx | + | + | + | + | + | + | + | + | + | + | + | + | + |
| 60095 | 1 | SPKETWLRL | *Rv3714c* | 4159219 | 4159245 | NA | + | + | + | + | + | + | + | + | + | + | + | + | + |
| 53355 | 1 | RDGQLTIKAERTEQKDFDGR | *Rv2031c* | 2278663 | 2278722 | hspx | + | + | + | + | + | + | + | + | + | + | + | + | + |
| 37835 | 1 | LLSVLAAVGL | *Rv0125* | 151187 | 151216 | pepA | + | + | + | + | + | + | + | + | + | + | + | + | + |
| 1546 | 1 | AGGYKASDMWGPKEDPAWQR | *Rv3804c* | 4265960 | 4266019 | fbpA | + | + | + | + | + | + | + | + | + | + | + | + | + |
| 62998 | 1 | TASPGAASGPKVVIDGKDQN | *Rv3763* | 4209167 | 4209226 | lpqH | + | + | + | + | + | + | + | + | + | + | + | + | + |
| 21482 | 1 | GNNLPAKFLEGFVRTSNIKF | *Rv3804c* | 4265810 | 4265869 | fbpA | + | + | + | + | + | + | + | + | + | + | + | + | + |
| 61914 | 1 | STSRVLDPAAGVTQLLSGVTNLQAQ | *Rv2945c* | 3290846 | 3290920 | lppx | + | + | + | + | + | + | + | + | + | + | + | + | + |
| 33972 | 1 | KVDSLLGITSADVDVRANPLAAKGV | *Rv2945c* | 3291026 | 3291100 | lppx | + | + | + | + | + | + | + | + | + | + | + | + | + |
| 23487 | 1 | GYTSGTGQGNASATKDGSHY | *Rv3763* | 4209377 | 4209436 | lpqH | + | + | + | + | + | + | + | + | + | + | + | + | + |
| 56043 | 1 | RTEQKDFDGRSEFAYGSFVR | *Rv2031c* | 2278633 | 2278692 | hspx | + | + | + | + | + | + | + | + | + | + | + | + | + |
| 20979 | 1 | GLLDPSQAMGPTLIGLAMGD | *Rv3804c* | 4266020 | 4266079 | fbpA | + | + | + | + | + | + | + | + | + | + | + | + | + |
| 79056 | 1 | QSIGDLIAEAMDKVGNEGV | *Rv0440* | 529067 | 529123 | groEL | + | + | + | + | + | + | + | + | + | + | + | + | + |
| 9414 | 1 | DMWEHAFYL | *Rv3846* | 4321181 | 4321207 | sodA | + | + | + | + | + | + | + | + | + | + | + | + | + |
| 49635 | 1 | PTGPASVQGMSQDPVAVAASNNPEL | *Rv2875* | 3187165 | 3187239 | mpt70 | + | + | + | + | + | + | + | + | + | + | + | + | + |
| 13836 | 1 | EQGVPFRVQGDNISVKLFDDWSNLG | *Rv2945c* | 3290936 | 3291010 | lppx | + | + | + | + | + | + | + | + | + | + | + | + | + |
| 56317 | 1 | RVILHSPYGLRVHGPLAL | *Rv3296* | 3678806 | 3678859 | lhr | + | + | + | + | + | + | + | + | + | + | + | + | + |
| 24876 | 1 | HSWEYWGAQLNAMKGDLQ | *Rv1886c* | 2134911 | 2134964 | fbpB | + | + | + | + | + | + | + | + | + | + | + | + | + |
| 16332 | 1 | FIYAGSLSA | *Rv1886c* | 2135295 | 2135321 | fbpB | + | + | + | + | + | + | + | + | + | + | + | + | + |
| 44802 | 1 | NLLVTGFDT | *Rv3812* | 4277348 | 4277374 | PE_PGRS62 | + | + | + | + | + | + | + | + | + | + | + | + | + |
| 27389 | 1 | ILTVSVAVSEGKPTEKHIQI | *Rv2031c* | 2278513 | 2278572 | hspx | + | + | + | + | + | + | + | + | + | + | + | + | + |
| 73964 | 1 | YFVTDPERQEAVLED | *Rv0440* | 529208 | 529252 | groEL | + | + | + | + | + | + | + | + | + | + | + | + | + |
| 13473 | 1 | ENFVRSSNL | *Rv1886c* | 2135034 | 2135060 | fbpB | + | + | + | + | + | + | + | + | + | + | + | + | + |
| 31342 | 1 | KILVQANEAETTTASG | *Rv3418c* | 3837208 | 3837255 | groES | + | + | + | + | + | + | + | + | + | + | + | + | + |
| 9415 | 1 | DMWEHAFYLQ | *Rv3846* | 4321181 | 4321210 | sodA | + | + | + | + | + | + | + | + | + | + | + | + | + |
| 32710 | 1 | KPGCDAPAY | *Rv3467* | 3884645 | 3884671 | NA | + | + | + | + | + | + | + | + | + | + | + | + | + |
| 31749 | 1 | KLAGGVAVI | *Rv0440* | 529712 | 529738 | groEL | + | + | + | + | + | + | + | + | + | + | + | + | + |
| 35316 | 1 | LDVAEGDTVIYSKYG | *Rv3418c* | 3837064 | 3837108 | groES | + | + | + | + | + | + | + | + | + | + | + | + | + |
| 54704 | 1 | RLPLVLPAV | *Rv2715* | 3028584 | 3028610 | NA | + | + | + | + | + | + | + | + | + | + | + | + | + |
| 52025 | 1 | QQFIYAGSLSALLDPSQGM | *Rv1886c* | 2135271 | 2135327 | fbpB | + | + | + | + | + | + | + | + | + | + | + | + | + |
| 5422 | 1 | AVLEDPYILLVSSKV | *Rv0440* | 529238 | 529282 | groEL | + | + | + | + | + | + | + | + | + | + | + | + | + |
| 44100 | 1 | NGVFDFPDSGTHSWEYWGAQ | *Rv3804c* | 4265720 | 4265779 | fbpA | + | + | + | + | + | + | + | + | + | + | + | + | + |
| 25561 | 1 | IDELKTNSSLLTSILTYHVV | *Rv2875* | 3187369 | 3187428 | mpt70 | + | + | + | + | + | + | + | + | + | + | + | + | + |
| 39169 | 1 | LRPTFDTRLMRLEDEMKEGR | *Rv2031c* | 2278783 | 2278842 | hspx | + | + | + | + | + | + | + | + | + | + | + | + | + |
| 16926 | 1 | FLTSELPQWLSANRAVKP | *Rv1886c* | 2135397 | 2135450 | fbpB | + | + | + | + | + | + | + | + | + | + | + | + | + |
| 2490 | 1 | ALEAFAIAVA | *Rv1694* | 1918156 | 1918185 | tlyA | + | + | + | + | + | + | + | + | + | + | + | + | + |
| 35787 | 1 | LFAAFPSFAGLRPTFDTRLM | *Rv2031c* | 2278813 | 2278872 | hspx | + | + | + | + | + | + | + | + | + | + | + | + | + |
| 52626 | 1 | QTSPANVVGTRQTLQGASVTVTGQG | *Rv2875* | 3187435 | 3187509 | mpt70 | + | + | + | + | + | + | + | + | + | + | + | + | + |
| 41001 | 1 | MAAHKGLMNIALAISAQQVN | *Rv0934* | 1042454 | 1042513 | pstS1 | + | + | + | + | + | + | + | + | + | + | + | + | + |
| 64580 | 1 | TKGLTSVHVAVRTTGKVDSLLGITS | *Rv2945c* | 3291071 | 3291145 | lppx | + | + | + | + | + | + | + | + | + | + | + | + | + |
| 32213 | 1 | KLVANNTRL | *Rv1886c* | 2135127 | 2135153 | fbpB | + | + | + | + | + | + | + | + | + | + | + | + | + |
| 44020 | 1 | NGKVLAAMYQGTIKTWDDPQ | *Rv0934* | 1042550 | 1042609 | pstS1 | + | + | + | + | + | + | + | + | + | + | + | + | + |
| 50870 | 1 | QGGLAPVMMQQTFST | *Rv3207c* | 3583894 | 3583938 | NA | + | + | + | + | + | + | + | + | + | + | + | + | + |
| 73378 | 1 | YAIVNNRQKDAATAQTLQAF | *Rv0934* | 1043060 | 1043119 | pstS1 | + | + | + | + | + | + | + | + | + | + | + | + | + |
| 53356 | 1 | RDGQLTIKAERTEQKDFDGRS | *Rv2031c* | 2278660 | 2278722 | hspx | + | + | + | + | + | + | + | + | + | + | + | + | + |
| 42897 | 1 | MTYAAPLFV | *Rv0667* | 760167 | 760193 | rpoB | + | + | + | + | + | + | + | + | + | + | + | + | + |
| 67657 | 1 | VAKVNIKPLEDKILVQ | *Rv3418c* | 3837241 | 3837288 | groES | + | + | + | + | + | + | + | + | + | + | + | + | + |
| 79000 | 1 | KASVPGGGDMGGMDF | *Rv0440* | 530183 | 530227 | groEL | + | + | + | + | + | + | + | + | + | + | + | + | + |
| 72613 | 1 | WIFGWNRLPRLVHLACI | *Rv1623c* | 1825501 | 1825551 | cydA | + | + | + | + | + | + | + | + | + | + | + | + | + |
| 5251 | 1 | AVAGAAILV | *Rv3763* | 4209068 | 4209094 | lpqH | + | + | + | + | + | + | + | + | + | + | + | + | + |
| 37140 | 1 | LLDAHIPQL | *Rv0287* | 351531 | 351557 | esxG | + | + | + | + | + | + | + | + | + | + | + | + | + |
| 2007 | 1 | AILTGGQVISEEVGL | *Rv0440* | 529478 | 529522 | groEL | + | + | + | + | + | + | + | + | + | + | + | + | + |
| 73049 | 1 | WRRRPLSSALLSFGLLLGGLPL | *Rv2223c* | 2495322 | 2495387 | NA | + | + | + | + | + | + | + | + | + | + | + | + | + |
| 8411 | 1 | DGISTTKITGTIPASSVKMLDPGAK | *Rv2945c* | 3290756 | 3290830 | lppx | + | + | + | + | + | + | + | + | + | + | + | + | + |
| 7470 | 1 | DAATAQTLQAFLHWAITD | *Rv0934* | 1043087 | 1043140 | pstS1 | + | + | + | + | + | + | + | + | + | + | + | + | + |
| 40529 | 1 | LVPVNHLPLTLPL | *Rv1184c* | 1324739 | 1324777 | NA | + | + | + | + | + | + | + | + | + | + | + | + | + |
| 54355 | 1 | RKHRIEDAVRNAKAAVEEGIVAG | *Rv0440* | 529775 | 529843 | groEL | + | + | + | + | + | + | + | + | + | + | + | + | + |
| 40622 | 1 | LVVADLSFI | *Rv1694* | 1918387 | 1918413 | tlyA | + | + | + | + | + | + | + | + | + | + | + | + | + |
| 70903 | 1 | VSDLKSSTAVIPGYPVAGQV | *Rv1926c* | 2179212 | 2179271 | mpt63 | + | + | + | + | + | + | + | + | + | + | + | + | + |
| 18781 | 1 | GASVTVTGQGNSLKVGNADVVCGGV | *Rv2875* | 3187480 | 3187554 | mpt70 | + | + | + | + | + | + | + | + | + | + | + | + | + |
| 12366 | 1 | EHRVKRGLTVAVAGA | *Rv3763* | 4209047 | 4209082 | lpqH | + | + | + | + | + | + | + | + | + | + | + | + | + |
| 33951 | 1 | KVALEAPLKQIAFNS | *Rv0440* | 529928 | 529972 | groEL | + | + | + | + | + | + | + | + | + | + | + | + | + |
| 35785 | 1 | LFAAFPSFA | *Rv2031c* | 2278846 | 2278872 | hspx | + | + | + | + | + | + | + | + | + | + | + | + | + |
| 2879 | 1 | ALSGQLNPQVNLVDTLNSGQYTVFA | *Rv2875* | 3187255 | 3187329 | mpt70 | + | + | + | + | + | + | + | + | + | + | + | + | + |
| 49254 | 1 | PRSLFPEFSELFAAFPSFAG | *Rv2031c* | 2278843 | 2278902 | hspx | + | + | + | + | + | + | + | + | + | + | + | + | + |
| 57047 | 1 | SAWQGDTGITYQAWQAQW | *Rv0288* | 351968 | 352021 | esxH | + | + | + | + | + | + | + | + | + | + | + | + | + |
| 21987 | 1 | GRAELSSIVVLLTNNTA | *Rv1623c* | 1825351 | 1825401 | cydA | + | + | + | + | + | + | + | + | + | + | + | + | + |
| 17759 | 1 | FSKLPASTIDELKTNSSLLTSILTY | *Rv2875* | 3187345 | 3187419 | mpt70 | + | + | + | + | + | + | + | + | + | + | + | + | + |
| 62187 | 1 | SVKMLDPGAKSARPATVWIAQDGSH | *Rv2945c* | 3290711 | 3290785 | lppx | + | + | + | + | + | + | + | + | + | + | + | + | + |
| 21670 | 1 | GPKEDPAWQRNDPLLNVGKL | *Rv3804c* | 4265930 | 4265989 | fbpA | + | + | + | + | + | + | + | + | + | + | + | + | + |
| 50966 | 1 | QGTGSGAGIAQAAAGTVNIG | *Rv0934* | 1042364 | 1042423 | pstS1 | + | + | + | + | + | + | + | + | + | + | + | + | + |
| 6901 | 1 | CQTYKWETF | *Rv1886c* | 2135448 | 2135474 | fbpB | + | + | + | + | + | + | + | + | + | + | + | + | + |
| 17836 | 1 | FSRPGLPVEYLQVPSPSM | *Rv1886c* | 2135694 | 2135747 | fbpB | + | + | + | + | + | + | + | + | + | + | + | + | + |
| 16879 | 1 | FLRIATSARVLAAPLPT | *Rv1242* | 1384673 | 1384723 | NA | + | + | + | + | + | + | + | + | + | + | + | + | + |
| 905 | 1 | AEFLENFVRSSNLKFQDA | *Rv1886c* | 2135019 | 2135072 | fbpB | + | + | + | + | + | + | + | + | + | + | + | + | + |
| 3094 | 1 | AMGDAGGYK | *Rv1886c* | 2135223 | 2135249 | fbpB | + | + | + | + | + | + | + | + | + | + | + | + | + |
| 8900 | 1 | DKILVQANEAETTTA | *Rv3418c* | 3837214 | 3837258 | groES | + | + | + | + | + | + | + | + | + | + | + | + | + |
| 18897 | 1 | GCQTYKWETFLTSELPQW | *Rv1886c* | 2135424 | 2135477 | fbpB | + | + | + | + | + | + | + | + | + | + | + | + | + |
| 1473 | 1 | AGDQSIGDLIAEAMD | *Rv0440* | 529058 | 529102 | groEL | + | + | + | + | + | + | + | + | + | + | + | + | + |
| 3248 | 1 | ANEAETTTASGLVIPD | *Rv3418c* | 3837193 | 3837240 | groES | + | + | + | + | + | + | + | + | + | + | + | + | + |
| 39281 | 1 | LSANRAVKPTGSAAIGLS | *Rv1886c* | 2135370 | 2135423 | fbpB | + | + | + | + | + | + | + | + | + | + | + | + | + |
| 20912 | 1 | GLIDIAPHQISSV | *Rv0341* | 409458 | 409496 | iniB | + | + | + | + | + | + | + | + | + | + | + | + | + |
| 7799 | 1 | DDIKATYDKGILTVSVAVSE | *Rv2031c* | 2278543 | 2278602 | hspx | + | + | + | + | + | + | + | + | + | + | + | + | + |
| 4426 | 1 | ASGLVIPDTAKEKPQE | *Rv3418c* | 3837169 | 3837216 | groES | + | + | + | + | + | + | + | + | + | + | + | + | + |
| 12311 | 1 | EGVVLLLVGALVL | *Rv0589* | 686869 | 686907 | mce2A | + | + | + | + | + | + | + | + | + | + | + | + | + |
| 20910 | 1 | GLIDIAPHQI | *Rv0341* | 409458 | 409487 | iniB | + | + | + | + | + | + | + | + | + | + | + | + | + |
| 69308 | 1 | VKRGLTVAVAGAAILVAGLS | *Rv3763* | 4209047 | 4209106 | lpqH | + | + | + | + | + | + | + | + | + | + | + | + | + |
| 7472 | 1 | DAATAQTLQAFLHWAITDGN | *Rv0934* | 1043087 | 1043146 | pstS1 | + | + | + | + | + | + | + | + | + | + | + | + | + |
| 62989 | 1 | TASGLVIPDTAKEKPQE | *Rv3418c* | 3837169 | 3837219 | groES | + | + | + | + | + | + | + | + | + | + | + | + | + |
| 68189 | 1 | VEGAGDTDAIAGRVA | *Rv0440* | 529598 | 529642 | groEL | + | + | + | + | + | + | + | + | + | + | + | + | + |
| 13839 | 1 | EQIAATAAISAGDQS | *Rv0440* | 529028 | 529072 | groEL | + | + | + | + | + | + | + | + | + | + | + | + | + |
| 54349 | 1 | RKGRLAALAIA | *Rv1860* | 2107766 | 2107798 | apa | + | + | + | + | + | + | + | + | + | + | + | + | + |
| 22884 | 1 | GTVVAVGPGRWDEDGE | *Rv3418c* | 3837121 | 3837168 | groES | + | + | + | + | + | + | + | + | + | + | + | + | + |
| 20744 | 1 | GLAALAVAVSPPAAAGDLVGPGCAE | *Rv2875* | 3187075 | 3187149 | mpt70 | + | + | + | + | + | + | + | + | + | + | + | + | + |
| 32860 | 1 | KPRDDAAAL | *Rv0670* | 769903 | 769929 | end | + | + | + | + | + | + | + | + | + | + | + | + | + |
| 1197 | 1 | AETPGCVAYIGISFLDQASQ | *Rv0934* | 1042829 | 1042888 | pstS1 | + | + | + | + | + | + | + | + | + | + | + | + | + |
| 20911 | 1 | GLIDIAPHQISS | *Rv0341* | 409458 | 409493 | iniB | + | + | + | + | + | + | + | + | + | + | + | + | + |
| 19646 | 1 | GFVRTSNIKFQDAYNAGGGH | *Rv3804c* | 4265780 | 4265839 | fbpA | + | + | + | + | + | + | + | + | + | + | + | + | + |
| 69798 | 1 | VLTDGNPPEV | *Rv3763* | 4209308 | 4209337 | lpqH | + | + | + | + | + | + | + | + | + | + | + | + | + |
| 18959 | 1 | GDEATGANIVKVALE | *Rv0440* | 529898 | 529942 | groEL | + | + | + | + | + | + | + | + | + | + | + | + | + |
| 42342 | 1 | MPVGGQSSF | *Rv1886c* | 2135514 | 2135540 | fbpB | + | + | + | + | + | + | + | + | + | + | + | + | + |
| 38049 | 1 | LMIGTAAAV | *Rv1886c* | 2135799 | 2135825 | fbpB | + | + | + | + | + | + | + | + | + | + | + | + | + |
| 31443 | 1 | KITGTATGVDMANPMSPVNK | *Rv3763* | 4209437 | 4209496 | lpqH | + | + | + | + | + | + | + | + | + | + | + | + | + |
| 20180 | 1 | GHRRMVFRFLTSPIEI | *Rv3106* | 3474793 | 3474840 | fprA | + | + | + | + | + | + | + | + | + | + | + | + | + |
| 62899 | 1 | TAKEKPQEGTVVAVGP | *Rv3418c* | 3837145 | 3837192 | groES | + | + | + | + | + | + | + | + | + | + | + | + | + |
| 55530 | 1 | RRARWVVRMLTSLLMFPGRD | *Rv1255c* | 1402877 | 1402936 | NA | + | + | + | + | + | + | + | + | + | + | + | + | + |
| 58022 | 1 | SGETTTAAGTTASPGAASGP | *Rv3763* | 4209137 | 4209196 | lpqH | + | + | + | + | + | + | + | + | + | + | + | + | + |
| 73578 | 1 | YDQSGLSVVMPVGGQSSFYS | *Rv3804c* | 4266290 | 4266349 | fbpA | + | + | + | + | + | + | + | + | + | + | + | + | + |
| 58144 | 1 | SGNFLLPDAQSIQAAAAGFA | *Rv0934* | 1042922 | 1042981 | pstS1 | + | + | + | + | + | + | + | + | + | + | + | + | + |
| 73690 | 1 | YEKIGAELVKEVAKKTDDVAG | *Rv0440* | 528800 | 528862 | groEL | + | + | + | + | + | + | + | + | + | + | + | + | + |
| 35391 | 1 | LEDKILVQANEAETTT | *Rv3418c* | 3837217 | 3837264 | groES | + | + | + | + | + | + | + | + | + | + | + | + | + |
| 20369 | 1 | GILTVSVAV | *Rv2031c* | 2278549 | 2278575 | hspx | + | + | + | + | + | + | + | + | + | + | + | + | + |
| 75153 | 1 | YNGEEYLILSARDVLAVVSK | *Rv3418c* | 3836989 | 3837048 | groES | + | + | + | + | + | + | + | + | + | + | + | + | + |
| 72965 | 1 | WPTLIGLAM | *Rv0129c* | 156965 | 156991 | fbpC | + | + | + | + | + | + | + | + | + | + | + | + | + |
| 21078 | 1 | GLPVEYLQV | *Rv3804c* | 4266491 | 4266517 | fbpA | + | + | + | + | + | + | + | + | + | + | + | + | + |
| 78960 | 1 | EKIGAELVKEVAKK | *Rv0440* | 528803 | 528844 | groEL | + | + | + | + | + | + | + | + | + | + | + | + | + |
| 64081 | 1 | THSWEYWGAQLNAMKPDLQR | *Rv3804c* | 4265690 | 4265749 | fbpA | + | + | + | + | + | + | + | + | + | + | + | + | + |
| 47209 | 1 | PDTAKEKPQEGTVVA | *Rv3418c* | 3837154 | 3837198 | groES | + | + | + | + | + | + | + | + | + | + | + | + | + |
| 29558 | 1 | IYAGSLSAL | *Rv1886c* | 2135292 | 2135318 | fbpB | + | + | + | + | + | + | + | + | + | + | + | + | + |
| 13215 | 1 | ELPQWLSANR | *Rv1886c* | 2135409 | 2135438 | fbpB | + | + | + | + | + | + | + | + | + | + | + | + | + |
| 52431 | 1 | QSSFYSDWY | *Rv1886c* | 2135499 | 2135525 | fbpB | + | + | + | + | + | + | + | + | + | + | + | + | + |
| 43881 | 1 | NFLLPDAQSIQAAAAGFASK | *Rv0934* | 1042928 | 1042987 | pstS1 | + | + | + | + | + | + | + | + | + | + | + | + | + |
| 8464 | 1 | DGNPPEVKSVGLGNVNGVTL | *Rv3763* | 4209317 | 4209376 | lpqH | + | + | + | + | + | + | + | + | + | + | + | + | + |
| 3505 | 1 | APDGYPIINYEYAIVNNRQK | *Rv0934* | 1043027 | 1043086 | pstS1 | + | + | + | + | + | + | + | + | + | + | + | + | + |
| 9649 | 1 | DPIELNATLSAVA | *Rv0589* | 687307 | 687345 | mce2A | + | + | + | + | + | + | + | + | + | + | + | + | + |
| 4334 | 1 | ASATKDGSHYKITGTATGVD | *Rv3763* | 4209407 | 4209466 | lpqH | + | + | + | + | + | + | + | + | + | + | + | + | + |
| 25363 | 1 | IANNTRVWVYCGNGKPSDLG | *Rv3804c* | 4265870 | 4265929 | fbpA | + | + | + | + | + | + | + | + | + | + | + | + | + |
| 12402 | 1 | EIAVEQAALQSAWQGDTG | *Rv0288* | 351938 | 351991 | esxH | + | + | + | + | + | + | + | + | + | + | + | + | + |
| 40162 | 1 | LTSELPGWLQANRHVKPTGS | *Rv3804c* | 4266170 | 4266229 | fbpA | + | + | + | + | + | + | + | + | + | + | + | + | + |
| 43504 | 1 | NDPLLNVGKLIANNTRVWVY | *Rv3804c* | 4265900 | 4265959 | fbpA | + | + | + | + | + | + | + | + | + | + | + | + | + |
| 55324 | 1 | RPRLDSITY | *Rv1280c* | 1432565 | 1432591 | oppA | + | + | + | + | + | + | + | + | + | + | + | + | + |
| 36046 | 1 | LGACLALWLSGCSSPKPDAEEQGVP | *Rv2945c* | 3291206 | 3291280 | lppx | + | + | + | + | + | + | + | + | + | + | + | + | + |
| 75786 | 1 | YSKYGGTEIKYNGEE | *Rv3418c* | 3837034 | 3837078 | groES | + | + | + | + | + | + | + | + | + | + | + | + | + |
| 1539 | 1 | AGGVAVIKAGAATEV | *Rv0440* | 529718 | 529762 | groEL | + | + | + | + | + | + | + | + | + | + | + | + | + |
| 68529 | 1 | VFNFPPNGTHSWEYWGAQ | *Rv1886c* | 2134938 | 2134991 | fbpB | + | + | + | + | + | + | + | + | + | + | + | + | + |
| 20820 | 1 | GLEPGVVAEK | *Rv0440* | 529973 | 530002 | groEL | + | + | + | + | + | + | + | + | + | + | + | + | + |
| 63956 | 1 | TGSAAIGLSMAGSSAMIL | *Rv1886c* | 2135343 | 2135396 | fbpB | + | + | + | + | + | + | + | + | + | + | + | + | + |
| 73524 | 1 | YDAYFTDAGGITPGNSV | *Rv3497c* | 3916768 | 3916818 | mce4C | + | + | + | + | + | + | + | + | + | + | + | + | + |
| 56884 | 1 | SALTLAIYHPQQFVYAGAMS | *Rv3804c* | 4266080 | 4266139 | fbpA | + | + | + | + | + | + | + | + | + | + | + | + | + |
| 9924 | 1 | DQVHFQPLPPAVVKLSDALI | *Rv0934* | 1043162 | 1043221 | pstS1 | + | + | + | + | + | + | + | + | + | + | + | + | + |
| 74768 | 1 | YLLDGLRAQ | *Rv1886c* | 2135613 | 2135639 | fbpB | + | + | + | + | + | + | + | + | + | + | + | + | + |
| 78996 | 1 | IEDAVRNAKAAVEEG | *Rv0440* | 529787 | 529831 | groEL | + | + | + | + | + | + | + | + | + | + | + | + | + |
| 60061 | 1 | SPGFGTTVDFPAVPGALGEN | *Rv0934* | 1042742 | 1042801 | pstS1 | + | + | + | + | + | + | + | + | + | + | + | + | + |
| 21275 | 1 | GMGPSLIGL | *Rv1886c* | 2135250 | 2135276 | fbpB | + | + | + | + | + | + | + | + | + | + | + | + | + |
| 55199 | 1 | RPKVEGLEY | *Rv2182c* | 2445246 | 2445272 | NA | + | + | + | + | + | + | + | + | + | + | + | + | + |
| 529 | 1 | AAYHPQQFIYAGSLSALL | *Rv1886c* | 2135289 | 2135342 | fbpB | + | + | + | + | + | + | + | + | + | + | + | + | + |
| 49699 | 1 | PTLIGLAMGDAGGYKASDMW | *Rv3804c* | 4265990 | 4266049 | fbpA | + | + | + | + | + | + | + | + | + | + | + | + | + |
| 1581 | 1 | AGKPLLIIAEDVEGE | *Rv0440* | 529328 | 529372 | groEL | + | + | + | + | + | + | + | + | + | + | + | + | + |
| 70124 | 1 | VNLVDTLNSGQYT | *Rv2875* | 3187282 | 3187320 | mpt70 | + | + | + | + | + | + | + | + | + | + | + | + | + |
| 46625 | 1 | NYIPQQLAL | *Rv3812* | 4278038 | 4278064 | PE_PGRS62 | + | + | + | + | + | + | + | + | + | + | + | + | + |
| 21093 | 1 | GLRAQDDFSGWDINTPAFEW | *Rv3804c* | 4266350 | 4266409 | fbpA | + | + | + | + | + | + | + | + | + | + | + | + | + |
| 726 | 1 | ADLVPTATLLDTY | *Rv0589* | 687652 | 687690 | mce2A | + | + | + | + | + | + | + | + | + | + | + | + | + |
| 49870 | 1 | PVGGQSSFYSDWYQPACGKA | *Rv3804c* | 4266260 | 4266319 | fbpA | + | + | + | + | + | + | + | + | + | + | + | + | + |
| 49862 | 1 | PVEYLQVPSPSMGRD | *Rv3804c* | 4266467 | 4266511 | fbpA | + | + | + | + | + | + | + | + | + | + | + | + | + |
| 73361 | 1 | YAGTLQSLGADIASEQAVLS | *Rv3019c* | 3378882 | 3378941 | esxR | + | + | + | + | + | + | + | + | + | + | + | + | + |
| 41943 | 1 | MLGHAGDMAGYAGTLQSL | *Rv0288* | 351878 | 351931 | esxH | + | + | + | + | + | + | + | + | + | + | + | + | + |
| 55156 | 1 | RPGCDAPAY | *Rv1945* | 2197084 | 2197110 | NA | + | + | + | + | + | + | + | + | + | + | + | + | + |
| 1525 | 1 | AGGGVTLLQAAPTLD | *Rv0440* | 529838 | 529882 | groEL | + | + | + | + | + | + | + | + | + | + | + | + | + |
| 18896 | 1 | GCQTYKWETFLTSELPGWLQ | *Rv3804c* | 4266200 | 4266259 | fbpA | + | + | + | + | + | + | + | + | + | + | + | + | + |
| 17199 | 1 | FNLWGPAFHERYPNVTITA | *Rv0934* | 1042307 | 1042363 | pstS1 | + | + | + | + | + | + | + | + | + | + | + | + | + |
| 60263 | 1 | SPSMGRDIPVAFLA | *Rv3803c* | 4265295 | 4265336 | fbpD | + | + | + | + | + | + | + | + | + | + | + | + | + |
| 18526 | 1 | GAASGPKVVIDGKDQNVTGS | *Rv3763* | 4209179 | 4209238 | lpqH | + | + | + | + | + | + | + | + | + | + | + | + | + |
| 3403 | 1 | ANRHVKPTGSAVVGLSMAAS | *Rv3804c* | 4266140 | 4266199 | fbpA | + | + | + | + | + | + | + | + | + | + | + | + | + |
| 459 | 1 | AATGIAAVLTDGNPPEVKSV | *Rv3763* | 4209287 | 4209346 | lpqH | + | + | + | + | + | + | + | + | + | + | + | + | + |
| 56994 | 1 | SASMGRDIKVQFQG | *Rv0129c* | 157379 | 157420 | fbpC | + | + | + | + | + | + | + | + | + | + | + | + | + |
| 55882 | 1 | RSLFPEFSELFAAFPSFAGL | *Rv2031c* | 2278840 | 2278899 | hspx | + | + | + | + | + | + | + | + | + | + | + | + | + |
| 53172 | 1 | RANPLAAKGVCTYNDEQGVPFRVQG | *Rv2945c* | 3290981 | 3291055 | lppx | + | + | + | + | + | + | + | + | + | + | + | + | + |
| 67211 | 1 | TVWIAQDGSHHLVRASIDLGSGSIQ | *Rv2945c* | 3290666 | 3290740 | lppx | + | + | + | + | + | + | + | + | + | + | + | + | + |
| 57417 | 1 | SEFAYGSFVRTVSLPVGADE | *Rv2031c* | 2278603 | 2278662 | hspx | + | + | + | + | + | + | + | + | + | + | + | + | + |
| 21000 | 1 | GLLHHAPSL | *Rv2220* | 2488536 | 2488562 | glnA1 | + | + | + | + | + | + | + | + | + | + | + | + | + |
| 69646 | 1 | VLMGGVPGVE | *Rv2780* | 3087291 | 3087320 | ald | + | + | + | + | + | + | + | + | + | + | + | + | + |
| 5245 | 1 | AVAASNNPELTTLTAALSGQLNPQV | *Rv2875* | 3187210 | 3187284 | mpt70 | + | + | + | + | + | + | + | + | + | + | + | + | + |
| 65499 | 1 | TPAFEWYYQSGLSIVMPV | *Rv1886c* | 2135532 | 2135585 | fbpB | + | + | + | + | + | + | + | + | + | + | + | + | + |
| 74889 | 1 | YLQVPSPSMGRDIKVQFQ | *Rv1886c* | 2135667 | 2135720 | fbpB | + | + | + | + | + | + | + | + | + | + | + | + | + |
| 71346 | 1 | VTGSVVCTTAAGNVNIAIGG | *Rv3763* | 4209227 | 4209286 | lpqH | + | + | + | + | + | + | + | + | + | + | + | + | + |
| 16924 | 1 | FLTSELPQW | *Rv1886c* | 2135424 | 2135450 | fbpB | + | + | + | + | + | + | + | + | + | + | + | + | + |
| 42195 | 1 | MNDGKRAVTSAVLVVLGACLALWLS | *Rv2945c* | 3291251 | 3291325 | lppx | + | + | + | + | + | + | + | + | + | + | + | + | + |
| 34823 | 1 | LAIYHPQQFVYAGAMSGLLD | *Rv3804c* | 4266068 | 4266127 | fbpA | + | + | + | + | + | + | + | + | + | + | + | + | + |
| 18509 | 1 | GAAILVAGLSGCSSNKSTTG | *Rv3763* | 4209077 | 4209136 | lpqH | + | + | + | + | + | + | + | + | + | + | + | + | + |
| 21439 | 1 | GNGKPSDLGGNNLPAKFLEG | *Rv3804c* | 4265837 | 4265896 | fbpA | + | + | + | + | + | + | + | + | + | + | + | + | + |
| 5196 | 1 | ATVLAQALVREGLRN | *Rv0440* | 528878 | 528922 | groEL | + | + | + | + | + | + | + | + | + | + | + | + | + |
| 33162 | 1 | KRIPLDVAEGDTVIYS | *Rv3418c* | 3837073 | 3837120 | groES | + | + | + | + | + | + | + | + | + | + | + | + | + |
| 32215 | 1 | KLVANNTRLWVYCGNGTP | *Rv1886c* | 2135100 | 2135153 | fbpB | + | + | + | + | + | + | + | + | + | + | + | + | + |
| 70013 | 1 | VMRLYPVRLTTTMTR | *Rv3333c* | 3720465 | 3720509 | NA | + | + | + | + | + | + | + | + | + | + | + | + | + |
| 26269 | 1 | IGLSMAGSSAMILAAY | *Rv1886c* | 2135334 | 2135381 | fbpB | + | + | + | + | + | + | + | + | + | + | + | + | + |
| 38169 | 1 | LNAMKGDLQSSLGAG | *Rv1886c* | 2134893 | 2134937 | fbpB | + | + | + | + | + | + | + | + | + | + | + | + | + |
| 35403 | 1 | LEDPYEKIGAELVKE | *Rv0440* | 528788 | 528832 | groEL | + | + | + | + | + | + | + | + | + | + | + | + | + |
| 73362 | 1 | YAGTLQSLGAEIAVEQAA | *Rv0288* | 351908 | 351961 | esxH | + | + | + | + | + | + | + | + | + | + | + | + | + |
| 59627 | 1 | SMAGSSAMI | *Rv1886c* | 2135346 | 2135372 | fbpB | + | + | + | + | + | + | + | + | + | + | + | + | + |
| 52030 | 1 | QQFVYAGAMSGLLDPSQAMG | *Rv3804c* | 4266050 | 4266109 | fbpA | + | + | + | + | + | + | + | + | + | + | + | + | + |
| 4682 | 1 | ASPVAQSYL | *Rv1174c* | 1305795 | 1305821 | TB8.4 | + | + | + | + | + | + | + | + | + | + | + | + | + |
| 3865 | 1 | APTLDELKLEGDEAT | *Rv0440* | 529868 | 529912 | groEL | + | + | + | + | + | + | + | + | + | + | + | + | + |
| 19050 | 1 | GDLVGPGCAEYAAANPTGPASVQGM | *Rv2875* | 3187120 | 3187194 | mpt70 | + | + | + | + | + | + | + | + | + | + | + | + | + |
| 3081 | 1 | AMEDLVRAYHAMSSTHEA | *Rv0288* | 352028 | 352081 | esxH | + | + | + | + | + | + | + | + | + | + | + | + | + |
| 21909 | 1 | GQLLRRVRSRLARL | *Rv3201c* | 3576752 | 3576793 | NA | + | + | + | + | + | + | + | + | + | + | + | + | + |
| 51633 | 1 | QMPYQPVQSPTQVEA | *Rv2290* | 2562962 | 2563006 | lppO | + | + | + | + | + | + | + | + | + | + | + | + | + |
| 32668 | 1 | KPDAEEQGVPVSPTASDPALLAEIR | *Rv2945c* | 3291161 | 3291235 | lppx | + | + | + | + | + | + | + | + | + | + | + | + | + |
| 38916 | 1 | LQNAASIAGLFLTTE | *Rv0440* | 530108 | 530152 | groEL | + | + | + | + | + | + | + | + | + | + | + | + | + |
| 13787 | 1 | EPYLDPATM | *Rv2903c* | 3213228 | 3213254 | lepB | + | + | + | + | + | + | + | + | + | + | + | + | + |
| 12151 | 1 | EGDTVIYSKYGGTEIK | *Rv3418c* | 3837049 | 3837096 | groES | + | + | + | + | + | + | + | + | + | + | + | + | + |
| 4132 | 1 | ARDVLAVVSK | *Rv3418c* | 3836989 | 3837018 | groES | + | + | + | + | + | + | + | + | + | + | + | + | + |
| 23837 | 1 | HFQPLPPAVV | *Rv0934* | 1043171 | 1043200 | pstS1 | + | + | + | + | + | + | + | + | + | + | + | + | + |
| 58124 | 1 | SGLSIVMPVGGQSSFYSD | *Rv1886c* | 2135505 | 2135558 | fbpB | + | + | + | + | + | + | + | + | + | + | + | + | + |
| 57415 | 1 | SEFAYGSFVRTVSL | *Rv2031c* | 2278621 | 2278662 | hspx | + | + | + | + | + | + | + | + | + | + | + | + | + |
| 37668 | 1 | LLPLLEKVIGAGKPL | *Rv0440* | 529298 | 529342 | groEL | + | + | + | + | + | + | + | + | + | + | + | + | + |
| 41064 | 1 | MAKTIAYDEEARR | *Rv0440* | 528608 | 528646 | groEL | + | + | + | + | + | + | + | + | + | + | + | + | + |
| 23155 | 1 | GVSTANATVYMIDSVLMPP | *Rv2875* | 3187549 | 3187605 | mpt70 | + | + | + | + | + | + | + | + | + | + | + | + | + |
| 21780 | 1 | GPSLIGLAM | *Rv1886c* | 2135244 | 2135270 | fbpB | + | + | + | + | + | + | + | + | + | + | + | + | + |
| 21371 | 1 | GNADVVCGGVSTANATVYMIDSVLM | *Rv2875* | 3187525 | 3187599 | mpt70 | + | + | + | + | + | + | + | + | + | + | + | + | + |
| 20821 | 1 | GLEPGVVAEKV | *Rv0440* | 529973 | 530005 | groEL | + | + | + | + | + | + | + | + | + | + | + | + | + |
| 31838 | 1 | KLFDDWSNLGSISELSTSRVLDPAA | *Rv2945c* | 3290891 | 3290965 | lppx | + | + | + | + | + | + | + | + | + | + | + | + | + |
| 3422 | 1 | ANSPALYLLDGLRAQDDFSG | *Rv3804c* | 4266380 | 4266439 | fbpA | + | + | + | + | + | + | + | + | + | + | + | + | + |
| 15309 | 1 | FAYGSFVRTVSLPVGA | *Rv2031c* | 2278609 | 2278656 | hspx | + | + | + | + | + | + | + | + | + | + | + | + | + |
| 73069 | 1 | WSEYSRFVGDVFGAPLA | *Rv1623c* | 1825600 | 1825650 | cydA | + | + | + | + | + | + | + | + | + | + | + | + | + |
| 66092 | 1 | TRRRLLAVLIAL | *Rv0203* | 241535 | 241570 | NA | + | + | + | + | + | + | + | + | + | + | + | + | + |
| 60262 | 1 | SPSMGRDIKVQFQS | *Rv1886c* | 2135664 | 2135705 | fbpB | + | + | + | + | + | + | + | + | + | + | + | + | + |
| 38515 | 1 | LPGTAVVPL | *Rv0934* | 1042637 | 1042663 | pstS1 | + | + | + | + | + | + | + | + | + | + | + | + | + |
| 15116 | 1 | EYWGAQLNAMKGDLQSSLGA | *Rv1886c* | 2134896 | 2134955 | fbpB | + | + | + | + | + | + | + | + | + | + | + | + | + |
| 21434 | 1 | GNGGMVTGCAETPGCVAYIG | *Rv0934* | 1042802 | 1042861 | pstS1 | + | + | + | + | + | + | + | + | + | + | + | + | + |
| 72008 | 1 | VYEDLLAAGVADPVK | *Rv0440* | 530048 | 530092 | groEL | + | + | + | + | + | + | + | + | + | + | + | + | + |
| 67156 | 1 | TVSLPVGADEDDIKATYDKG | *Rv2031c* | 2278573 | 2278632 | hspx | + | + | + | + | + | + | + | + | + | + | + | + | + |
| 73306 | 1 | WYYQSGLSI | *Rv1886c* | 2135544 | 2135570 | fbpB | + | + | + | + | + | + | + | + | + | + | + | + | + |
| 27786 | 1 | IPAEFLENF | *Rv1886c* | 2135052 | 2135078 | fbpB | + | + | + | + | + | + | + | + | + | + | + | + | + |
| 55334 | 1 | RPRYEIFVY | *Rv2476c* | 2779884 | 2779910 | gdh | + | + | + | + | + | + | + | + | + | + | + | + | + |
| 17838 | 1 | FSRPGLPVEYLQVPSPSMGR | *Rv3804c* | 4266470 | 4266529 | fbpA | + | + | + | + | + | + | + | + | + | + | + | + | + |
| 78980 | 1 | GLKRGIEKAVEKVTETL | *Rv0440* | 528947 | 528997 | groEL | + | + | + | + | + | + | + | + | + | + | + | + | + |
| 38334 | 1 | LNSGQYTVFAPTNAAFSKLPASTID | *Rv2875* | 3187300 | 3187374 | mpt70 | + | + | + | + | + | + | + | + | + | + | + | + | + |
| 27901 | 1 | IPKLVANNT | *Rv1886c* | 2135133 | 2135159 | fbpB | + | + | + | + | + | + | + | + | + | + | + | + | + |
| 55192 | 1 | RPKPDYSAM | *Rv3378c* | 3792463 | 3792489 | NA | + | + | + | + | + | + | + | + | + | + | + | + | + |
| 9613 | 1 | DPDKDVDIMVRDGQLTIKAE | *Rv2031c* | 2278693 | 2278752 | hspx | + | + | + | + | + | + | + | + | + | + | + | + | + |
| 5381 | 1 | AVINTTCNYGQ | *Rv1174c* | 1305873 | 1305905 | TB8.4 | + | + | + | + | + | + | + | + | + | + | + | + | + |
| 41089 | 1 | MAMMARDTAEAAKWGG | *Rv0288* | 352088 | 352135 | esxH | + | + | + | + | + | + | + | + | + | + | + | + | + |
| 67233 | 1 | TVYMIDSVLMPPA | *Rv2875* | 3187570 | 3187608 | mpt70 | + | + | + | + | + | + | + | + | + | + | + | + | + |
| 31902 | 1 | KLIANNTRV | *Rv3804c* | 4265909 | 4265935 | fbpA | + | + | + | + | + | + | + | + | + | + | + | + | + |
| 39170 | 1 | LRPTFDTRLMRLEDEMKEGRYE | *Rv2031c* | 2278777 | 2278842 | hspx | + | + | + | + | + | + | + | + | + | + | + | + | + |
| 67588 | 1 | VAFRAGLVMEAGSKVT | *Rv0589* | 686965 | 687012 | mce2A | + | + | + | + | + | + | + | + | + | + | + | + | + |
| 55191 | 1 | RPKPDTETY | *Rv3689* | 4130381 | 4130407 | NA | + | + | + | + | + | + | + | + | + | + | + | + | + |
| 69514 | 1 | VLGRLDQKL | *Rv1860* | 2108231 | 2108257 | apa | + | + | + | + | + | + | + | + | + | + | + | + | + |
| 3196 | 1 | AMSSTHEANTMAMMARDT | *Rv0288* | 352058 | 352111 | esxH | + | + | + | + | + | + | + | + | + | + | + | + | + |
| 75152 | 1 | YNGEEYLILSARDVL | *Rv3418c* | 3837004 | 3837048 | groES | + | + | + | + | + | + | + | + | + | + | + | + | + |
| 55188 | 1 | RPKIDDHDY | *Rv1641* | 1852543 | 1852569 | infC | + | + | + | + | + | + | + | + | + | + | + | + | + |
| 39011 | 1 | LQVPSPSMGRDIKVQFQSGG | *Rv3804c* | 4266440 | 4266499 | fbpA | + | + | + | + | + | + | + | + | + | + | + | + | + |
| 41872 | 1 | MKPDLQRALGATPNTGPAPQGA | *Rv3804c* | 4265645 | 4265710 | fbpA | + | + | + | + | + | + | + | + | + | + | + | + | + |
| 67656 | 1 | VAKVNIKPLEDKILV | *Rv3418c* | 3837244 | 3837288 | groES | + | + | + | + | + | + | + | + | + | + | + | + | + |
| 4280 | 1 | ARVIMRSAIG | *Rv2190c* | 2453216 | 2453245 | NA | + | + | + | + | + | + | + | + | + | + | + | + | + |
| 42638 | 1 | MSQIMYNYPAMLGHAGDM | *Rv0288* | 351848 | 351901 | esxH | + | + | + | + | + | + | + | + | + | + | + | + | + |
| 79110 | 1 | VKVTRSALQNAASIA | *Rv0440* | 530087 | 530131 | groEL | + | + | + | + | + | + | + | + | + | + | + | + | + |
| 55315 | 1 | RPREATIIY | *Rv2823c* | 3129905 | 3129931 | NA | + | + | + | + | + | + | + | + | + | + | + | + | + |
| 39408 | 1 | LSGVTNLQAQGTEVIDGISTTKITG | *Rv2945c* | 3290801 | 3290875 | lppx | + | + | + | + | + | + | + | + | + | + | + | + | + |
| 20890 | 1 | GLGNVNGVTLGYTSGTGQGN | *Rv3763* | 4209347 | 4209406 | lpqH | + | + | + | + | + | + | + | + | + | + | + | + | + |
| 9923 | 1 | DQVHFQPLPPAVVKLSDAL | *Rv0934* | 1043162 | 1043218 | pstS1 | + | + | + | + | + | + | + | + | + | + | + | + | + |
| 1629 | 1 | AGNVNIAIGGAATGIAAVLTD | *Rv3763* | 4209257 | 4209319 | lpqH | + | + | + | + | + | + | + | + | + | + | + | + | + |
| 28040 | 1 | IPRDEVRVM | *Rv1461* | 1647958 | 1647984 | NA | + | + | + | + | + | + | + | + | + | + | + | + | + |
| 32692 | 1 | KPEKEKASVPGGGDM | *Rv0440* | 530168 | 530212 | groEL | + | + | + | + | + | + | + | + | + | + | + | + | + |
| 9352 | 1 | DMANPMSPVNKSFEIEVTCS | *Rv3763* | 4209464 | 4209523 | lpqH | + | + | + | + | + | + | + | + | + | + | + | + | + |
| 50444 | 1 | QDAYNAGGGHNGVFDFPDSG | *Rv3804c* | 4265750 | 4265809 | fbpA | + | + | + | + | + | + | + | + | + | + | + | + | + |
| 8685 | 1 | DIKVQFQSG | *Rv1886c* | 2135661 | 2135687 | fbpB | + | + | + | + | + | + | + | + | + | + | + | + | + |
| 34232 | 1 | KVVIDGKDQNVTGSVVCTTA | *Rv3763* | 4209197 | 4209256 | lpqH | + | + | + | + | + | + | + | + | + | + | + | + | + |
| 64714 | 1 | TLAGKGISVV | *Rv3803c* | 4265178 | 4265207 | fbpD | + | + | + | + | + | + | + | + | + | + | + | + | + |
| 61178 | 1 | SSLLTSILTYHVVAGQTSPANVVGT | *Rv2875* | 3187390 | 3187464 | mpt70 | + | + | + | + | + | + | + | + | + | + | + | + | + |
| 18059 | 1 | FTRRFAASMVG | *Rv1291c* | 1445799 | 1445831 | NA | + | + | + | + | + | + | + | + | + | + | + | + | + |
| 72312 | 1 | WDINTPAFEWYDQSGLSVVM | *Rv3804c* | 4266320 | 4266379 | fbpA | + | + | + | + | + | + | + | + | + | + | + | + | + |
| 41099 | 1 | MANPMSPVNKSFEIEVTCS | *Rv3763* | 4209467 | 4209523 | lpqH | + | + | + | + | + | + | + | + | + | + | + | + | + |
| 58051 | 1 | SGGNNSPAVYLLDGLRAQ | *Rv1886c* | 2135613 | 2135666 | fbpB | + | + | + | + | + | + | + | + | + | + | + | + | + |
| 41889 | 4 | MKVKNTIAATSFAAAGLAALAVAVS | *Rv2875* | 3187030 | 3187104 | mpt70 | - | + | + | + | + | + | + | + | + | + | + | + | + |
| 41840 | 4 | MKIRLHTLLAVLTAAPLLLA | *Rv0934* | 1042115 | 1042174 | pstS1 | - | + | + | + | + | + | + | + | + | + | + | + | + |
| 42102 | 4 | MMAHAGDMAGYAGTLQSLGA | *Rv3019c* | 3378912 | 3378971 | esxR | + | + | + | + | + | + | - | + | + | + | + | + | + |
| 53312 | 4 | RCRRALRQIGVLERPVGDSS | *Rv3017c* | 3376583 | 3376642 | esxQ | + | + | + | + | - | + | + | + | + | + | + | + | + |
| 75496 | 4 | YQGWQTQWNQALEDLVRAYQ | *Rv3019c* | 3378792 | 3378851 | esxR | + | + | + | + | + | + | - | + | + | + | + | + | + |
| 6348 | 4 | CGSKPPSGSPETGAGAGTVA | *Rv0934* | 1042184 | 1042243 | pstS1 | - | + | + | + | + | + | + | + | + | + | + | + | + |
| 23064 | 4 | GVNAPIPGI | *Rv1158c* | 1284509 | 1284535 | NA | + | + | + | + | + | - | + | + | + | + | + | + | + |
| 965 | 4 | AEHQAIIRDVLTASD | *Rv1198* | 1341081 | 1341125 | esxL | + | + | + | + | + | - | + | + | + | + | + | + | + |
| 51117 | 4 | QIPQYKHSVVMGVNKAKVPG | *Rv0309* | 378396 | 378455 | NA | + | + | - | + | + | + | + | + | + | + | + | + | + |
| 59748 | 4 | SMSGTHESNTMAMLARDGAE | *Rv3019c* | 3378732 | 3378791 | esxR | + | + | + | + | + | + | - | + | + | + | + | + | + |
| 7703 | 4 | DCGTIRVGSFRGRWLDPRHA | *Rv3017c* | 3376523 | 3376582 | esxQ | + | + | + | + | - | + | + | + | + | + | + | + | + |
| 62384 | 4 | SVVMGVNKAK | *Rv0309* | 378417 | 378446 | NA | + | + | - | + | + | + | + | + | + | + | + | + | + |
| 69467 | 4 | VLERPVGDSSDCGTIRVGSF | *Rv3017c* | 3376553 | 3376612 | esxQ | + | + | + | + | - | + | + | + | + | + | + | + | + |
| 42640 | 4 | MSQIMYNYPAMMAHAGDMAG | *Rv3019c* | 3378942 | 3379001 | esxR | + | + | + | + | + | + | - | + | + | + | + | + | + |
| 8590 | 4 | DIASEQAVLSSAWQGDTGIT | *Rv3019c* | 3378852 | 3378911 | esxR | + | + | + | + | + | + | - | + | + | + | + | + | + |
| 42739 | 4 | MTANVGDMAGYTGTTQSLGA | *Rv3017c* | 3376763 | 3376822 | esxQ | + | + | + | + | - | + | + | + | + | + | + | + | + |
| 28327 | 4 | IRLHTLLAVLTAAPLLLAAA | *Rv0934* | 1042121 | 1042180 | pstS1 | - | + | + | + | + | + | + | + | + | + | + | + | + |
| 41088 | 4 | MAMLARDGAEAAKWGG | *Rv3019c* | 3378714 | 3378761 | esxR | + | + | + | + | + | + | - | + | + | + | + | + | + |
| 57048 | 4 | SAWQGDTGITYQGWQTQWNQ | *Rv3019c* | 3378822 | 3378881 | esxR | + | + | + | + | + | + | - | + | + | + | + | + | + |
| 57719 | 4 | SFAAAGLAALAVAVSPPAA | *Rv2875* | 3187060 | 3187116 | mpt70 | - | + | + | + | + | + | + | + | + | + | + | + | + |
| 8591 | 4 | DIASERTAPSRACQGDLGMS | *Rv3017c* | 3376703 | 3376762 | esxQ | + | + | + | + | - | + | + | + | + | + | + | + | + |
| 53974 | 4 | RGRWLDPRHAGPATAADAGD | *Rv3017c* | 3376493 | 3376552 | esxQ | + | + | + | + | - | - | + | + | + | + | + | + | + |
| 37112 | 4 | LLAVLTAAPL | *Rv0934* | 1042136 | 1042165 | pstS1 | - | - | + | + | + | + | + | + | + | + | + | + | + |
| 50756 | 4 | QFGDVDAHGAMIRAQ | *Rv1793* | 2030709 | 2030753 | esxN | - | + | + | + | + | - | + | + | + | + | + | + | + |
| 43166 | 4 | NAHGQKVQAAGNNMA | *Rv1793* | 2030898 | 2030942 | esxN | + | - | + | + | + | - | + | + | + | + | + | + | + |
| 34174 | 4 | KVQAAGNNMAQTDSA | *Rv1793* | 2030913 | 2030957 | esxN | + | - | + | + | + | - | + | + | + | + | + | + | + |
| 54988 | 4 | RNFQVIYEQANAHGQ | *Rv1793* | 2030868 | 2030912 | esxN | + | - | + | + | + | - | + | + | + | + | + | + | + |
| 42642 | 4 | MSQSMYSYPAMTANVGDMAG | *Rv3017c* | 3376793 | 3376852 | esxQ | + | + | + | + | - | + | + | - | + | + | + | + | + |
| 29574 | 4 | IYEQANAHGQKVQAA | *Rv1793* | 2030883 | 2030927 | esxN | + | - | + | + | + | - | + | + | + | + | + | + | + |
| 12062 | 4 | EFQTVSNQL | *Rv3812* | 4276751 | 4276777 | PE_PGRS62 | - | - | + | + | + | - | + | + | + | + | + | + | + |
| 967 | 4 | AEHQAIVRDVLAAGD | *Rv1793* | 2030769 | 2030813 | esxN | - | - | + | + | + | - | + | + | + | + | + | + | + |
| 7530 | 4 | DAHGAMIRAQAASLE | *Rv1793* | 2030724 | 2030768 | esxN | - | - | + | + | + | - | + | + | + | + | + | + | + |
| 7531 | 4 | DAHGAMIRAQAGLLE | *Rv2346c* | 2626098 | 2626142 | esxO | - | - | + | + | - | - | + | + | + | + | + | + | + |
| 41766 | 4 | MIRAQAGLLEAEHQA | *Rv2346c* | 2626083 | 2626127 | esxO | - | + | + | + | - | - | - | + | - | - | + | + | + |
| 27900 | 4 | IPKLRQGSY | *Rv2666* | 2983305 | 2983331 | NA | - | + | + | - | + | - | - | - | - | - | + | + | + |
| 8651 | 4 | DIGLHIIVTCQMSQAYKATMDK | *Rv3871* | 4350333 | 4350398 | NA | + | - | - | + | - | - | - | + | - | - | + | + | + |
| 72323 | 3 | WDQAYRKPITYDTLWQADTD | *Rv1980c* | 2223571 | 2223630 | mpt64 | - | + | - | - | + | - | - | - | + | - | - | - | + |
| 19348 | 3 | GELLPEAAGPTQVLVPRSAI | *Rv1980c* | 2223361 | 2223420 | mpt64 | - | + | - | - | + | - | - | - | + | - | - | - | + |
| 26805 | 3 | IKIFMLVTAVVLLCCSGVAT | *Rv1980c* | 2223964 | 2224023 | mpt64 | - | + | - | - | + | - | - | - | + | - | - | - | + |
| 53370 | 3 | RDKFLSAATSSTPREAPYEL | *Rv1980c* | 2223751 | 2223810 | mpt64 | - | + | - | - | + | - | - | - | + | - | - | - | + |
| 48458 | 3 | PLPVVFPIVQGELSKQTGQQ | *Rv1980c* | 2223511 | 2223570 | mpt64 | - | + | - | - | + | - | - | - | + | - | - | - | + |
| 49925 | 3 | PVNYQNFAVTNDGVIFFFNP | *Rv1980c* | 2223421 | 2223480 | mpt64 | - | + | - | - | + | - | - | - | + | - | - | - | + |
| 28594 | 3 | ISLPSYYPDQKSLENYIAQT | *Rv1980c* | 2223811 | 2223870 | mpt64 | - | + | - | - | + | - | - | - | + | - | - | - | + |
| 25123 | 3 | HVYLDTVVLLGALAN | *Rv1986* | 2230377 | 2230421 | NA | - | + | - | - | + | - | - | - | + | - | - | - | + |
| 70980 | 3 | VSIAPNAGLDPVNYQNFAVT | *Rv1980c* | 2223451 | 2223510 | mpt64 | - | + | - | - | + | - | - | - | + | - | - | - | + |
| 22633 | 3 | GTDTGQACQIQMSDPAYNIN | *Rv1980c* | 2223871 | 2223930 | mpt64 | - | + | - | - | + | - | - | - | + | - | - | - | + |
| 75172 | 3 | YNINISLPSYYPDQKSLENY | *Rv1980c* | 2223823 | 2223882 | mpt64 | - | + | - | - | + | - | - | - | + | - | - | - | + |
| 51643 | 3 | QMSDPAYNINISLPSYYPDQ | *Rv1980c* | 2223841 | 2223900 | mpt64 | - | + | - | - | + | - | - | - | + | - | - | - | + |
| 27981 | 3 | IPPRGTQAVVLKVYQNAGGT | *Rv1980c* | 2223661 | 2223720 | mpt64 | - | + | - | - | + | - | - | - | + | - | - | - | + |
| 37059 | 3 | LKVYQNAGGTHPTTTYKAFD | *Rv1980c* | 2223631 | 2223690 | mpt64 | - | + | - | - | + | - | - | - | + | - | - | - | + |
| 73593 | 3 | YDTLWQADTDPLPVVFPIVQ | *Rv1980c* | 2223541 | 2223600 | mpt64 | - | + | - | - | + | - | - | - | + | - | - | - | + |
| 61834 | 3 | STPREAPYELNITSATYQSA | *Rv1980c* | 2223721 | 2223780 | mpt64 | - | + | - | - | + | - | - | - | + | - | - | - | + |
| 60540 | 3 | SQRIKSLEQQVGQVLVVREK | *Rv1985c* | 2229732 | 2229791 | NA | - | + | - | - | + | - | - | - | + | - | - | - | + |
| 1150 | 3 | AERLHVTPSAVSQRIKSLEQ | *Rv1985c* | 2229765 | 2229824 | NA | - | + | - | - | + | - | - | - | + | - | - | - | + |
| 24522 | 3 | HPTTTYKAFDWDQAYRKPIT | *Rv1980c* | 2223601 | 2223660 | mpt64 | - | + | - | - | + | - | - | - | + | - | - | - | + |
| 38245 | 3 | LNIYVRRWRTALHATVSALIVAI | *Rv1987* | 2231035 | 2231103 | NA | - | + | - | - | + | - | - | - | + | - | - | - | + |
| 33402 | 3 | KSLENYIAQTRDKFLSAATS | *Rv1980c* | 2223781 | 2223840 | mpt64 | - | + | - | - | + | - | - | - | + | - | - | - | + |
| 19359 | 3 | GELSKQTGQQVSIAPNAGLD | *Rv1980c* | 2223481 | 2223540 | mpt64 | - | + | - | - | + | - | - | - | + | - | - | - | + |
| 65965 | 3 | TQVLVPRSAIDSMLA | *Rv1980c* | 2223346 | 2223390 | mpt64 | - | + | - | - | + | - | - | - | + | - | - | - | + |
| 3629 | 3 | APKTYCEELKGTDTGQACQI | *Rv1980c* | 2223901 | 2223960 | mpt64 | - | + | - | - | + | - | - | - | + | - | - | - | + |
| 2048 | 3 | AIPPRGTQAVVLKVYQNAGG | *Rv1980c* | 2223664 | 2223723 | mpt64 | - | + | - | - | + | - | - | - | + | - | - | - | + |
| 14020 | 3 | ERLHVTPSAVSQRIKSLEQQ | *Rv1985c* | 2229762 | 2229821 | NA | - | + | - | - | + | - | - | - | + | - | - | - | + |
| 43447 | 3 | NDGVIFFFNPGELLPEAAGP | *Rv1980c* | 2223391 | 2223450 | mpt64 | - | + | - | - | + | - | - | - | + | - | - | - | + |
| 44381 | 3 | NITSATYQSAIPPRGTQAVV | *Rv1980c* | 2223691 | 2223750 | mpt64 | - | + | - | - | + | - | - | - | + | - | - | - | + |
| 28366 | 2 | IRQAGVQYSRADEEQQQA | *Rv3874* | 4352499 | 4352552 | esxB | - | - | - | - | - | - | - | - | - | - | - | - | - |
| 4942 | 2 | ATELNNALQNLARTISEAGQAMAS | *Rv3875* | 4352792 | 4352863 | esxA | - | - | - | - | - | - | - | - | - | - | - | - | - |
| 13920 | 2 | EQRDRILIITINRPKAKNAV | *Rv0222* | 265555 | 265614 | echA1 | - | - | - | - | - | - | - | - | - | - | - | - | - |
| 1020 | 2 | AEKPATEQAEPVHEVTNDDQ | *Rv3878* | 4357272 | 4357331 | NA | - | - | - | - | - | - | - | - | - | - | - | - | - |
| 41030 | 2 | MAEMKTDAATLAQEAGNFER | *Rv3874* | 4352274 | 4352333 | esxB | - | - | - | - | - | - | - | - | - | - | - | - | - |
| 1681 | 2 | AGSLQGQWRGAAGTA | *Rv3874* | 4352379 | 4352423 | esxB | - | - | - | - | - | - | - | - | - | - | - | - | - |
| 21423 | 2 | GNFERISGDLKTQIDQVESTAGSLQ | *Rv3874* | 4352319 | 4352393 | esxB | - | - | - | - | - | - | - | - | - | - | - | - | - |
| 33831 | 2 | KTQIDQVESTAGSLQ | *Rv3874* | 4352349 | 4352393 | esxB | - | - | - | - | - | - | - | - | - | - | - | - | - |
| 20015 | 2 | GGSGPMPAQLASAEKPATEQ | *Rv3878* | 4357236 | 4357295 | NA | - | - | - | - | - | - | - | - | - | - | - | - | - |
| 65198 | 2 | TMFAEQMKILVPVFTSND | *Rv0222* | 266182 | 266235 | echA1 | - | - | - | - | - | - | - | - | - | - | - | - | - |
| 511 | 2 | AAWGGSGSEAYQGVQQKWDA | *Rv3875* | 4352729 | 4352788 | esxA | - | - | - | - | - | - | - | - | - | - | - | - | - |
| 12206 | 2 | EGKQSLTKLAAAWGGSGSEA | *Rv3875* | 4352699 | 4352758 | esxA | - | - | - | - | - | - | - | - | - | - | - | - | - |
| 63010 | 2 | TATELNNALQNLARTISEAG | *Rv3875* | 4352789 | 4352848 | esxA | - | - | - | - | - | - | - | - | - | - | - | - | - |
| 46499 | 2 | NVTSIHSLL | *Rv3875* | 4352669 | 4352695 | esxA | - | - | - | - | - | - | - | - | - | - | - | - | - |
| 42795 | 2 | MTEQQWNFAGIEAAAS | *Rv3875* | 4352609 | 4352656 | esxA | - | - | - | - | - | - | - | - | - | - | - | - | - |
| 327 | 2 | AANKQKQELDEISTNIRQAG | *Rv3874* | 4352454 | 4352513 | esxB | - | - | - | - | - | - | - | - | - | - | - | - | - |
| 82520 | 2 | GAAGTAAQAAVVRFQEAANKQKQ | *Rv3874* | 4352406 | 4352474 | esxB | - | - | - | - | - | - | - | - | - | - | - | - | - |
| 1080 | 2 | AEMKTDAATLA | *Rv3874* | 4352277 | 4352309 | esxB | - | - | - | - | - | - | - | - | - | - | - | - | - |
| 85735 | 2 | LQNLARTISEAGQAMASTEGNVT | *Rv3875* | 4352813 | 4352881 | esxA | - | - | - | - | - | - | - | - | - | - | - | - | - |
| 12583 | 2 | EISTNIRQA | *Rv3874* | 4352484 | 4352510 | esxB | - | - | - | - | - | - | - | - | - | - | - | - | - |
| 52691 | 2 | QVESTAGSLQGQWRGAAGTAAQAAV | *Rv3874* | 4352364 | 4352438 | esxB | - | - | - | - | - | - | - | - | - | - | - | - | - |
| 13386 | 2 | EMKTDAATL | *Rv3874* | 4352280 | 4352306 | esxB | - | - | - | - | - | - | - | - | - | - | - | - | - |
| 28367 | 2 | IRQAGVQYSRADEEQQQALSSQMGF | *Rv3874* | 4352499 | 4352573 | esxB | - | - | - | - | - | - | - | - | - | - | - | - | - |
| 14209 | 2 | ESLVSDGLPGVKAAL | *Rv3878* | 4356843 | 4356887 | NA | - | - | - | - | - | - | - | - | - | - | - | - | - |
| 42796 | 2 | MTEQQWNFAGIEAAASAI | *Rv3875* | 4352609 | 4352662 | esxA | - | - | - | - | - | - | - | - | - | - | - | - | - |
| 21975 | 2 | GQWRGAAGTAAQAAV | *Rv3874* | 4352394 | 4352438 | esxB | - | - | - | - | - | - | - | - | - | - | - | - | - |
| 3935 | 2 | AQAAVVRFQEAANKQKQELD | *Rv3874* | 4352424 | 4352483 | esxB | - | - | - | - | - | - | - | - | - | - | - | - | - |
| 3064 | 2 | AMASTEGNV | *Rv3875* | 4352852 | 4352878 | esxA | - | - | - | - | - | - | - | - | - | - | - | - | - |
| 326 | 2 | AANKQKQELDEISTN | *Rv3874* | 4352454 | 4352498 | esxB | - | - | - | - | - | - | - | - | - | - | - | - | - |
| 12207 | 2 | EGKQSLTKLAAAWGGSGSEAYQGVQ | *Rv3875* | 4352699 | 4352773 | esxA | - | - | - | - | - | - | - | - | - | - | - | - | - |
| 52690 | 2 | QVESTAGSLQGQWRGAAGTA | *Rv3874* | 4352364 | 4352423 | esxB | - | - | - | - | - | - | - | - | - | - | - | - | - |
| 34949 | 2 | LARTISEAGQAMASTEGNVTGMFA | *Rv3875* | 4352822 | 4352893 | esxA | - | - | - | - | - | - | - | - | - | - | - | - | - |
| 55394 | 2 | RQAGVQYSRADEEQQ | *Rv3874* | 4352502 | 4352546 | esxB | - | - | - | - | - | - | - | - | - | - | - | - | - |
| 10984 | 2 | EAAASAIQGNVTSIHSLLDEGKQS | *Rv3875* | 4352642 | 4352713 | esxA | - | - | - | - | - | - | - | - | - | - | - | - | - |
| 58534 | 2 | SIHSLLDEGKQSLTKLAA | *Rv3875* | 4352678 | 4352731 | esxA | - | - | - | - | - | - | - | - | - | - | - | - | - |
| 85043 | 2 | LDEISTNIRQAGVQYSRAD | *Rv3874* | 4352478 | 4352534 | esxB | - | - | - | - | - | - | - | - | - | - | - | - | - |
| 189 | 2 | AAGTAAQAAVVRFQEAANKQKQELD | *Rv3874* | 4352409 | 4352483 | esxB | - | - | - | - | - | - | - | - | - | - | - | - | - |
| 44327 | 2 | NIRQAGVQY | *Rv3874* | 4352496 | 4352522 | esxB | - | - | - | - | - | - | - | - | - | - | - | - | - |
| 14773 | 2 | EVLVEQRDRILIITINR | *Rv0222* | 265543 | 265593 | echA1 | - | - | - | - | - | - | - | - | - | - | - | - | - |
| 61782 | 2 | STNIRQAGVQYSRAD | *Rv3874* | 4352490 | 4352534 | esxB | - | - | - | - | - | - | - | - | - | - | - | - | - |
| 50318 | 2 | QAMASTEGNVTGMFA | *Rv3875* | 4352849 | 4352893 | esxA | - | - | - | - | - | - | - | - | - | - | - | - | - |
| 20669 | 2 | GKQSLTKLAAAWGGSGSEA | *Rv3875* | 4352702 | 4352758 | esxA | - | - | - | - | - | - | - | - | - | - | - | - | - |
| 63011 | 2 | TATELNNALQNLARTISEAGQAMAS | *Rv3875* | 4352789 | 4352863 | esxA | - | - | - | - | - | - | - | - | - | - | - | - | - |
| 188 | 2 | AAGTAAQAAVVRFQEAA | *Rv3874* | 4352409 | 4352459 | esxB | - | - | - | - | - | - | - | - | - | - | - | - | - |
| 5028 | 2 | ATLAQEAGNFERISGDLK | *Rv3874* | 4352298 | 4352351 | esxB | - | - | - | - | - | - | - | - | - | - | - | - | - |
| 506 | 2 | AAVVRFQEAANKQKQEL | *Rv3874* | 4352430 | 4352480 | esxB | - | - | - | - | - | - | - | - | - | - | - | - | - |
| 61578 | 2 | STEGNVTGMFA | *Rv3875* | 4352861 | 4352893 | esxA | - | - | - | - | - | - | - | - | - | - | - | - | - |
| 70631 | 2 | VQQKWDATATELNNAL | *Rv3875* | 4352768 | 4352815 | esxA | - | - | - | - | - | - | - | - | - | - | - | - | - |
| 11228 | 2 | EAYQGVQQKWDATATEL | *Rv3875* | 4352753 | 4352803 | esxA | - | - | - | - | - | - | - | - | - | - | - | - | - |
| 21306 | 2 | GMLRERQHRLLYLASA | *Rv1977* | 2219892 | 2219939 | NA | - | - | - | - | - | - | - | - | - | - | - | - | - |
| 1078 | 2 | AEMKTDAA | *Rv3874* | 4352277 | 4352300 | esxB | - | - | - | - | - | - | - | - | - | - | - | - | - |
| 34928 | 2 | LAQEAGNFERISGDLKTQID | *Rv3874* | 4352304 | 4352363 | esxB | - | - | - | - | - | - | - | - | - | - | - | - | - |
| 328 | 2 | AANKQKQELDEISTNIRQAGVQYSR | *Rv3874* | 4352454 | 4352528 | esxB | - | - | - | - | - | - | - | - | - | - | - | - | - |
| 37041 | 2 | LKTQIDQVESTAGSL | *Rv3874* | 4352346 | 4352390 | esxB | - | - | - | - | - | - | - | - | - | - | - | - | - |
| 53077 | 2 | RADEEQQQAL | *Rv3874* | 4352526 | 4352555 | esxB | - | - | - | - | - | - | - | - | - | - | - | - | - |
| 41031 | 2 | MAEMKTDAATLAQEAGNFERISGDL | *Rv3874* | 4352274 | 4352348 | esxB | - | - | - | - | - | - | - | - | - | - | - | - | - |
| 63109 | 2 | TDAATLAQEAGNFER | *Rv3874* | 4352289 | 4352333 | esxB | - | - | - | - | - | - | - | - | - | - | - | - | - |
| 70686 | 2 | VQYSRADEEQQQALS | *Rv3874* | 4352514 | 4352558 | esxB | - | - | - | - | - | - | - | - | - | - | - | - | - |
| 3934 | 2 | AQAAVVRFQEAANKQ | *Rv3874* | 4352424 | 4352468 | esxB | - | - | - | - | - | - | - | - | - | - | - | - | - |
| 72888 | 2 | WNFAGIEAAASAIQGNVTSIHSL | *Rv3875* | 4352624 | 4352692 | esxA | - | - | - | - | - | - | - | - | - | - | - | - | - |
| 85635 | 2 | LNNALQNLARTISEAGQAM | *Rv3875* | 4352801 | 4352857 | esxA | - | - | - | - | - | - | - | - | - | - | - | - | - |
| 53078 | 2 | RADEEQQQALSSQMGF | *Rv3874* | 4352526 | 4352573 | esxB | - | - | - | - | - | - | - | - | - | - | - | - | - |
| 23584 | 2 | HAVYRTMMMHLLRLARSFGVLPV | *Rv1977* | 2220177 | 2220245 | NA | - | - | - | - | - | - | - | - | - | - | - | - | - |
| 42797 | 2 | MTEQQWNFAGIEAAASAIQG | *Rv3875* | 4352609 | 4352668 | esxA | - | - | - | - | - | - | - | - | - | - | - | - | - |
| 80451 | 2 | ARTISEAGQAMASTEGNVT | *Rv3875* | 4352825 | 4352881 | esxA | - | - | - | - | - | - | - | - | - | - | - | - | - |
| 21936 | 2 | GQPSQATQLLSTPVSQVTTQ | *Rv3878* | 4357020 | 4357079 | NA | - | - | - | - | - | - | - | - | - | - | - | - | - |
| 43210 | 2 | NALQNLARTISEA | *Rv3875* | 4352807 | 4352845 | esxA | - | - | - | - | - | - | - | - | - | - | - | - | - |
| 58225 | 2 | SGSEAYQGVQQKWDATATELNNALQ | *Rv3875* | 4352744 | 4352818 | esxA | - | - | - | - | - | - | - | - | - | - | - | - | - |
| 60 | 2 | AAASAIQGNVTSIHSL | *Rv3875* | 4352645 | 4352692 | esxA | - | - | - | - | - | - | - | - | - | - | - | - | - |
| 75495 | 2 | YQGVQQKWDATATELNNALQ | *Rv3875* | 4352759 | 4352818 | esxA | - | - | - | - | - | - | - | - | - | - | - | - | - |
| 43211 | 2 | NALQNLARTISEAGQAMA | *Rv3875* | 4352807 | 4352860 | esxA | - | - | - | - | - | - | - | - | - | - | - | - | - |
| 1079 | 2 | AEMKTDAATL | *Rv3874* | 4352277 | 4352306 | esxB | - | - | - | - | - | - | - | - | - | - | - | - | - |
| 35139 | 2 | LDEGKQSLTKLAAAWG | *Rv3875* | 4352693 | 4352740 | esxA | - | - | - | - | - | - | - | - | - | - | - | - | - |
| 32971 | 2 | KQELDEISTNIRQAG | *Rv3874* | 4352469 | 4352513 | esxB | - | - | - | - | - | - | - | - | - | - | - | - | - |
| 35140 | 2 | LDEISTNIRQAGVQY | *Rv3874* | 4352478 | 4352522 | esxB | - | - | - | - | - | - | - | - | - | - | - | - | - |
| 10705 | 2 | DVQPAEVVAAARDEGAGASP | *Rv3878* | 4357344 | 4357403 | NA | - | - | - | - | - | - | - | - | - | - | - | - | - |
| 70720 | 2 | VRFQEAANKQKQELD | *Rv3874* | 4352439 | 4352483 | esxB | - | - | - | - | - | - | - | - | - | - | - | - | - |
| 12585 | 2 | EISTNIRQAGVQYSRADEEQ | *Rv3874* | 4352484 | 4352543 | esxB | - | - | - | - | - | - | - | - | - | - | - | - | - |
| 28470 | 2 | ISEAGQAMASTEGNVTGMFA | *Rv3875* | 4352834 | 4352893 | esxA | - | - | - | - | - | - | - | - | - | - | - | - | - |
| 19863 | 2 | GGGVPAQAMDTGAGARPAAS | *Rv3878* | 4357416 | 4357475 | NA | - | - | - | - | - | - | - | - | - | - | - | - | - |
| 187 | 2 | AAGTAAQAAVVRFQE | *Rv3874* | 4352409 | 4352453 | esxB | - | - | - | - | - | - | - | - | - | - | - | - | - |
| 19028 | 2 | GDLKTQIDQVESTAGSL | *Rv3874* | 4352340 | 4352390 | esxB | - | - | - | - | - | - | - | - | - | - | - | - | - |
| 5661 | 2 | AWGGSGSEAYQGVQQKWDATATEL | *Rv3875* | 4352732 | 4352803 | esxA | - | - | - | - | - | - | - | - | - | - | - | - | - |
| 2060 | 2 | AIQGNVTSIHSLLDEGK | *Rv3875* | 4352657 | 4352707 | esxA | - | - | - | - | - | - | - | - | - | - | - | - | - |
| 21422 | 2 | GNFERISGDLKTQID | *Rv3874* | 4352319 | 4352363 | esxB | - | - | - | - | - | - | - | - | - | - | - | - | - |
| 41029 | 2 | MAEMKTDAATLAQEAGNF | *Rv3874* | 4352274 | 4352327 | esxB | - | - | - | - | - | - | - | - | - | - | - | - | - |
| 15177 | 2 | FAGIEAAASAIQGNV | *Rv3875* | 4352630 | 4352674 | esxA | - | - | - | - | - | - | - | - | - | - | - | - | - |
| 12979 | 2 | ELDEISTNIRQAGVQYSR | *Rv3874* | 4352475 | 4352528 | esxB | - | - | - | - | - | - | - | - | - | - | - | - | - |
| 38922 | 2 | LQNLARTI | *Rv3875* | 4352813 | 4352836 | esxA | - | - | - | - | - | - | - | - | - | - | - | - | - |
| 50981 | 2 | QGVQQKWDATATELNNALQNLART | *Rv3875* | 4352762 | 4352833 | esxA | - | - | - | - | - | - | - | - | - | - | - | - | - |
| 28364 | 2 | IRQAGVQYSR | *Rv3874* | 4352499 | 4352528 | esxB | - | - | - | - | - | - | - | - | - | - | - | - | - |
| 70687 | 2 | VQYSRADEEQQQALSSQMGF | *Rv3874* | 4352514 | 4352573 | esxB | - | - | - | - | - | - | - | - | - | - | - | - | - |
| 9920 | 2 | DQVESTAGSLQGQWRGAA | *Rv3874* | 4352361 | 4352414 | esxB | - | - | - | - | - | - | - | - | - | - | - | - | - |
| 43829 | 2 | NFERISGDLKTQIDQV | *Rv3874* | 4352322 | 4352369 | esxB | - | - | - | - | - | - | - | - | - | - | - | - | - |
| 42798 | 2 | MTEQQWNFAGIEAAASAIQGNVTSI | *Rv3875* | 4352609 | 4352683 | esxA | - | - | - | - | - | - | - | - | - | - | - | - | - |
| 4899 | 2 | ATATELNNALQNLARTI | *Rv3875* | 4352786 | 4352836 | esxA | - | - | - | - | - | - | - | - | - | - | - | - | - |
| 60616 | 2 | SRADEEQQQALSSQMGF | *Rv3874* | 4352523 | 4352573 | esxB | - | - | - | - | - | - | - | - | - | - | - | - | - |
| 46500 | 2 | NVTSIHSLLDEGKQSL | *Rv3875* | 4352669 | 4352716 | esxA | - | - | - | - | - | - | - | - | - | - | - | - | - |
| 82519 | 2 | GAAGTAAQAAVVRFQEAAN | *Rv3874* | 4352406 | 4352462 | esxB | - | - | - | - | - | - | - | - | - | - | - | - | - |
| 64305 | 2 | TIGDTTIPAGRRVLLLYGSA | *Rv1256c* | 1403641 | 1403700 | cyp130 | - | - | - | - | - | - | - | - | - | - | - | - | - |
| 28506 | 2 | ISGDLKTQIDQVESTAGSLQ | *Rv3874* | 4352334 | 4352393 | esxB | - | - | - | - | - | - | - | - | - | - | - | - | - |
| 37146 | 2 | LLDEGKQSL | *Rv3875* | 4352690 | 4352716 | esxA | - | - | - | - | - | - | - | - | - | - | - | - | - |
| 52689 | 2 | QVESTAGSLQGQWRG | *Rv3874* | 4352364 | 4352408 | esxB | - | - | - | - | - | - | - | - | - | - | - | - | - |
| 64457 | 2 | TISEAGQAMASTEGNV | *Rv3875* | 4352831 | 4352878 | esxA | - | - | - | - | - | - | - | - | - | - | - | - | - |
| 510 | 2 | AAWGGSGSEAYQGVQQKW | *Rv3875* | 4352729 | 4352782 | esxA | - | - | - | - | - | - | - | - | - | - | - | - | - |
| 19 | 2 | AAAAKLAGLVFPQPPAPIAV | *Rv3878* | 4356732 | 4356791 | NA | - | - | - | - | - | - | - | - | - | - | - | - | - |
| 46501 | 2 | NVTSIHSLLDEGKQSLTKLA | *Rv3875* | 4352669 | 4352728 | esxA | - | - | - | - | - | - | - | - | - | - | - | - | - |
| 1452 | 2 | AGARPAASPLAAPVDPSTPA | *Rv3878* | 4357452 | 4357511 | NA | - | - | - | - | - | - | - | - | - | - | - | - | - |
| 250 | 2 | AAKLAGLVFPQPPAP | *Rv3878* | 4356738 | 4356782 | NA | - | - | - | - | - | - | - | - | - | - | - | - | - |
| 56836 | 2 | SAIQGNVTSIHSLLDEGKQSLTKLA | *Rv3875* | 4352654 | 4352728 | esxA | - | - | - | - | - | - | - | - | - | - | - | - | - |
| 21976 | 2 | GQWRGAAGTAAQAAVVRFQE | *Rv3874* | 4352394 | 4352453 | esxB | - | - | - | - | - | - | - | - | - | - | - | - | - |
| 655 | 2 | ADEEQQQALSSQMGF | *Rv3874* | 4352529 | 4352573 | esxB | - | - | - | - | - | - | - | - | - | - | - | - | - |
| 44653 | 2 | NLARTISEAGQAMASTEGNV | *Rv3875* | 4352819 | 4352878 | esxA | - | - | - | - | - | - | - | - | - | - | - | - | - |
| 25716 | 2 | IEAAASAIQGNVTSIHSLLD | *Rv3875* | 4352639 | 4352698 | esxA | - | - | - | - | - | - | - | - | - | - | - | - | - |
| 7992 | 2 | DEGAGASPGQQPGGGVPAQA | *Rv3878* | 4357380 | 4357439 | NA | - | - | - | - | - | - | - | - | - | - | - | - | - |
| 2867 | 2 | ALRRLKGFDQILKLMSGMLR | *Rv1977* | 2219844 | 2219903 | NA | - | - | - | - | - | - | - | - | - | - | - | - | - |
| 87675 | 2 | QQWNFAGIEA | *Rv3875* | 4352618 | 4352647 | esxA | - | - | - | - | - | - | - | - | - | - | - | - | - |
| 71492 | 2 | VTSIHSLLDEGKQSLTKLAAAWGG | *Rv3875* | 4352672 | 4352743 | esxA | - | - | - | - | - | - | - | - | - | - | - | - | - |
| 20242 | 2 | GIEAAASAIQGNVTSIHSLLD | *Rv3875* | 4352636 | 4352698 | esxA | - | - | - | - | - | - | - | - | - | - | - | - | - |
| 20670 | 2 | GKQSLTKLAAAWGGSGSEAYQGVQ | *Rv3875* | 4352702 | 4352773 | esxA | - | - | - | - | - | - | - | - | - | - | - | - | - |
| 62797 | 2 | TAAQAAVVRF | *Rv3874* | 4352418 | 4352447 | esxB | - | - | - | - | - | - | - | - | - | - | - | - | - |
| 41028 | 2 | MAEMKTDAATLAQEA | *Rv3874* | 4352274 | 4352318 | esxB | - | - | - | - | - | - | - | - | - | - | - | - | - |
| 23748 | 2 | HEVTNDDQGDQGDVQPAEVV | *Rv3878* | 4357308 | 4357367 | NA | - | - | - | - | - | - | - | - | - | - | - | - | - |
| 10338 | 2 | DTFYDRAQEYSQVLQ | *Rv3879c* | 4359663 | 4359707 | NA | - | - | - | - | - | - | - | - | - | - | - | - | - |
