# Supplementary material for: Genome Sequencing and Analysis of BCG Vaccine Strains
Source: PLoS One. 2013 Aug 19;8(8):e71243. doi: 10.1371/journal.pone.0071243 (PMC3747166; doi:10.1371/journal.pone.0071243)
Supplement: Table S6 — Genes with Group 1 epitopes in H37Rv. “+”: epitope in this gene exists in this BCG strain; “×”: epitopes in this gene does not exist in this BCG strain. “+/×”: there are several epitopes in this gene, and some were lost in this strain. “NA” in column 4 denotes that there are no related references to support that this gene is the antigen. (DOC) [file pone.0071243.s006.doc]

**Table S6. Genes owning epitopes of Group 1 in H37Rv. “+” represents that epitopes in this gene are existing this BCG strain, while “×” not. “+/×” represents that there are several epitopes in this gene and some of epitopes were lost in this strain. “NA” in column 4 is that no related reference to support that this gene is the antigen.**

| Group | Gene | Protein | Reference | Epitopes num | M. bovis | Prague | Sweden | Frappier | Glaxo | Moreau | Phipps | China | Danish | Russia | Mexico | Tice | Pasteur | Tokyo |
| --- | --- | --- | --- | --- | --- | --- | --- | --- | --- | --- | --- | --- | --- | --- | --- | --- | --- | --- |
| 1 | *ald* | Secreted L-alanine dehydrogenase ALD (40 kDa antigen) (TB43) |  | 1 | + | + | + | + | + | + | + | + | + | + | + | + | + | + |
| 1 | *apa* | Unkown (Could Mediate Bacterial Attachment to host cells) |  | 2 | + | + | + | + | + | + | + | + | + | + | + | + | + | + |
| 1 | *cydA* | Cytochrome bd-I oxidase subunit I | NA | 4 | + | + | + | + | + | + | + | + | + | + | + | + | + | + |
| 1 | *end* | Endonuclease IV |  | 1 | + | + | + | + | + | + | + | + | + | + | + | + | + | + |
| 1 | *esxG* | Unknown (ESAT-6 like protein) |  | 2 | + | + | + | + | + | + | + | + | + | + | + | + | + | + |
| 1 | *esxH* | Low molecular weight protein antigen 7 ESXH (10kDa antigen) (CFP-7) (protein TB10.4) |  | 8 | + | + | + | + | + | + | + | + | + | + | + | + | + | + |
| 1 | *esxR* | Secreted ESAT-6 like protein ESXR (TB10.3)(ESAT-6 like protein 9) |  | 1 | + | + | + | + | + | + | + | + | + | + | + | + | + | + |
| 1 | *fbpA* | Secreted antigen 85-A FBPA (Mycolyl transferase85A) (fibronectin-binding protein A) (antigen 85 complexA) |  | 31 | + | + | + | + | + | + | + | + | + | + | + | + | + | + |
| 1 | *fbpB* | Secreted antigen 85-B fbpB (85B) (antigen 85complex B) |  | 42 | + | + | + | + | + | + | + | + | + | + | + | + | + | + |
| 1 | *fbpC* | Secreted antigen 85-C FBPC (85C) (antigen 85complex C) |  | 2 | + | + | + | + | + | + | + | + | + | + | + | + | + | + |
| 1 | *fbpD* | Secreted MPT51 |  | 2 | + | + | + | + | + | + | + | + | + | + | + | + | + | + |
| 1 | *fprA* | NADPH-ferredoxin reductase |  | 1 | + | + | + | + | + | + | + | + | + | + | + | + | + | + |
| 1 | *gdh* | NAD-dependent glutamate dehydrogenase |  | 1 | + | + | + | + | + | + | + | + | + | + | + | + | + | + |
| 1 | *glnA1* | Glutamine synthetase GLNA1 (glutamine synthase)(GS-I) |  | 1 | + | + | + | + | + | + | + | + | + | + | + | + | + | + |
| 1 | *groEL* | Chaperonin GroEL |  | 34 | + | + | + | + | + | + | + | + | + | + | + | + | + | + |
| 1 | *groES* | Co-chaperonin GroES |  | 23 | + | + | + | + | + | + | + | + | + | + | + | + | + | + |
| 1 | *hspX* | Heat shock protein hspX |  | 21 | + | + | + | + | + | + | + | + | + | + | + | + | + | + |
| 1 | *infC* | Translation initiation factor IF-3 |  | 1 | + | + | + | + | + | + | + | + | + | + | + | + | + | + |
| 1 | *iniB* | Isoniazid inductible gene protein INIB |  | 3 | + | + | + | + | + | + | + | + | + | + | + | + | + | + |
| 1 | *lepB* | Signal peptidase I LepB |  | 1 | + | + | + | + | + | + | + | + | + | + | + | + | + | + |
| 1 | *lhr* | ATP-dependent helicase |  | 1 | + | + | + | + | + | + | + | + | + | + | + | + | + | + |
| 1 | *lppO* | Lipoprotein lppO |  | 1 | + | + | + | + | + | + | + | + | + | + | + | + | + | + |
| 1 | *lppX* | Lipoprotein LppX |  | 15 | + | + | + | + | + | + | + | + | + | + | + | + | + | + |
| 1 | *lpqH* | 19 kDa lipoprotein antigen precursor LPQH |  | 21 | + | + | + | + | + | + | + | + | + | + | + | + | + | + |
| 1 | *mce2A* | MCE-family protein MCE2A |  | 5 | + | + | + | + | + | + | + | + | + | + | + | + | + | + |
| 1 | *mce4C* | MCE-family protein MCE4C |  | 2 | + | + | + | + | + | + | + | + | + | + | + | + | + | + |
| 1 | *mpt53* | Soluble Secreted antigen MPT53 precursor |  | 1 | + | + | + | + | + | + | + | + | + | + | + | + | + | + |
| 1 | *mpt63* | Immunogenic protein MPT63 (antigen MPT63) |  | 2 | + | + | + | + | + | + | + | + | + | + | + | + | + | + |
| 1 | *mpt70* | Major Secreted immunogenic protein MPT70 |  | 15 | + | + | + | + | + | + | + | + | + | + | + | + | + | + |
| 1 | *oppA* | Periplasmic oligopeptide-binding lipoproteinOppA |  | 1 | + | + | + | + | + | + | + | + | + | + | + | + | + | + |
| 1 | *PE_PGRS62* | PE-PGRS family protein |  | 2 | + | + | + | + | + | + | + | + | + | + | + | + | + | + |
| 1 | *pepA* | Serine protease PepA |  | 1 | + | + | + | + | + | + | + | + | + | + | + | + | + | + |
| 1 | *pstS1* | Periplasmic phosphate-binding lipoprotein PSTS1(PBP-1) (PSTS1) |  | 19 | + | + | + | + | + | + | + | + | + | + | + | + | + | + |
| 1 | *rpoB* | DNA-directed RNA polymerase subunit beta |  | 1 | + | + | + | + | + | + | + | + | + | + | + | + | + | + |
| 1 | *sodA* | Superoxide dismutase [Fe] SODA | NA | 2 | + | + | + | + | + | + | + | + | + | + | + | + | + | + |
| 1 | *TB8.4* | Low molecular weight T-cell antigen TB8.4 |  | 2 | + | + | + | + | + | + | + | + | + | + | + | + | + | + |
| 1 | *tlyA* | Cytotoxin |  | 2 | + | + | + | + | + | + | + | + | + | + | + | + | + | + |
| 1 | *Rv0200* | transmembrane protein | NA | 1 | + | + | + | + | + | + | + | + | + | + | + | + | + | + |
| 1 | *Rv0203* | Hypothetical protein |  | 1 | + | + | + | + | + | + | + | + | + | + | + | + | + | + |
| 1 | *Rv1184c* | Hypothetical protein | NA | 1 | + | + | + | + | + | + | + | + | + | + | + | + | + | + |
| 1 | *Rv1242* | Hypothetical protein | NA | 1 | + | + | + | + | + | + | + | + | + | + | + | + | + | + |
| 1 | *Rv1255c* | transcriptional regulatory protein | NA | 1 | + | + | + | + | + | + | + | + | + | + | + | + | + | + |
| 1 | *Rv1291c* | Hypothetical protein | NA | 1 | + | + | + | + | + | + | + | + | + | + | + | + | + | + |
| 1 | *Rv1461* | Hypothetical protein | NA | 1 | + | + | + | + | + | + | + | + | + | + | + | + | + | + |
| 1 | *Rv1945* | Hypothetical protein | NA | 1 | + | + | + | + | + | + | + | + | + | + | + | + | + | + |
| 1 | *Rv2182c* | 1. acylglycerol-3-phosphate O-acyltransferase | NA | 1 | + | + | + | + | + | + | + | + | + | + | + | + | + | + |
| 1 | *Rv2190c* | Hypothetical protein | NA | 1 | + | + | + | + | + | + | + | + | + | + | + | + | + | + |
| 1 | *Rv2223c* | exported protease | NA | 1 | + | + | + | + | + | + | + | + | + | + | + | + | + | + |
| 1 | *Rv2715* | Hypothetical protein | NA | 1 | + | + | + | + | + | + | + | + | + | + | + | + | + | + |
| 1 | *Rv2823c* | Hypothetical protein | NA | 1 | + | + | + | + | + | + | + | + | + | + | + | + | + | + |
| 1 | *Rv3201c* | ATP-dependent DNA helicase |  | 1 | + | + | + | + | + | + | + | + | + | + | + | + | + | + |
| 1 | *Rv3207c* | hypothetical protein | NA | 1 | + | + | + | + | + | + | + | + | + | + | + | + | + | + |
| 1 | *Rv3333c* | hypothetical protein | NA | 1 | + | + | + | + | + | + | + | + | + | + | + | + | + | + |
| 1 | *Rv3378c* | hypothetical protein | NA | 1 | + | + | + | + | + | + | + | + | + | + | + | + | + | + |
| 1 | *Rv3467* | hypothetical protein | NA | 1 | + | + | + | + | + | + | + | + | + | + | + | + | + | + |
| 1 | *Rv3689* | hypothetical protein | NA | 1 | + | + | + | + | + | + | + | + | + | + | + | + | + | + |
| 1 | *Rv3714c* | hypothetical protein | NA | 1 | + | + | + | + | + | + | + | + | + | + | + | + | + | + |

Reference

1. Andersen AB, Andersen P, Ljungqvist L (1992) Structure and function of a 40,000-molecular-weight protein antigen of Mycobacterium tuberculosis. Infect Immun 60: 2317-2323.

2. Hutter B, Singh M (1999) Properties of the 40 kDa antigen of Mycobacterium tuberculosis, a functional L-alanine dehydrogenase. Biochem J 343 Pt 3: 669-672.

3. Kumar P, Amara RR, Challu VK, Chadda VK, Satchidanandam V (2003) The Apa protein of Mycobacterium tuberculosis stimulates gamma interferon-secreting CD4+ and CD8+ T cells from purified protein derivative-positive individuals and affords protection in a guinea pig model. Infect Immun 71: 1929-1937.

4. Sable SB, Cheruvu M, Nandakumar S, Sharma S, Bandyopadhyay K, et al. (2011) Cellular immune responses to nine Mycobacterium tuberculosis vaccine candidates following intranasal vaccination. PLoS One 6: e22718.

5. Hammond AS, Klein MR, Corrah T, Fox A, Jaye A, et al. (2005) Mycobacterium tuberculosis genome-wide screen exposes multiple CD8 T cell epitopes. Clin Exp Immunol 140: 109-116.

6. Coler RN, Dillon DC, Skeiky YA, Kahn M, Orme IM, et al. (2009) Identification of Mycobacterium tuberculosis vaccine candidates using human CD4+ T-cells expression cloning. Vaccine 27: 223-233.

7. Ahmed RK, Rohava Z, Balaji KN, Hoffner SE, Gaines H, et al. (2012) Pattern recognition and cellular immune responses to novel Mycobacterium tuberculosis-antigens in individuals from Belarus. BMC Infect Dis 12: 41.

8. Skjot RL, Brock I, Arend SM, Munk ME, Theisen M, et al. (2002) Epitope mapping of the immunodominant antigen TB10.4 and the two homologous proteins TB10.3 and TB12.9, which constitute a subfamily of the esat-6 gene family. Infect Immun 70: 5446-5453.

9. Axelsson-Robertson R, Weichold F, Sizemore D, Wulf M, Skeiky YA, et al. (2010) Extensive major histocompatibility complex class I binding promiscuity for Mycobacterium tuberculosis TB10.4 peptides and immune dominance of human leucocyte antigen (HLA)-B*0702 and HLA-B*0801 alleles in TB10.4 CD8 T-cell responses. Immunology 129: 496-505.

10. Vordermeier HM, Pontarollo R, Karvonen B, Cockle P, Hecker R, et al. (2005) Synthetic peptide vaccination in cattle: induction of strong cellular immune responses against peptides derived from the Mycobacterium bovis antigen Rv3019c. Vaccine 23: 4375-4384.

11. Vordermeier HM, Dean GS, Rosenkrands I, Agger EM, Andersen P, et al. (2009) Adjuvants induce distinct immunological phenotypes in a bovine tuberculosis vaccine model. Clin Vaccine Immunol 16: 1443-1448.

12. Strong M, Goulding CW (2006) Structural proteomics and computational analysis of a deadly pathogen: combating Mycobacterium tuberculosis from multiple fronts. Methods Biochem Anal 49: 245-269.

13. Deenadayalan A, Sundaramurthi JC, Raja A (2010) Immunological and proteomic analysis of preparative isoelectric focusing separated culture filtrate antigens of Mycobacterium tuberculosis. Exp Mol Pathol 88: 156-162.

14. Fu LM (2006) Exploring drug action on Mycobacterium tuberculosis using affymetrix oligonucleotide genechips. Tuberculosis (Edinb) 86: 134-143.

15. Ohara N, Ohara-Wada N, Kitaura H, Nishiyama T, Matsumoto S, et al. (1997) Analysis of the genes encoding the antigen 85 complex and MPT51 from Mycobacterium avium. Infect Immun 65: 3680-3685.

16. Domenech P, Barry CE, 3rd, Cole ST (2001) Mycobacterium tuberculosis in the post-genomic age. Curr Opin Microbiol 4: 28-34.

17. Romero IC, Mehaffy C, Burchmore RJ, Dobos-Elder K, Brennan P, et al. (2010) Identification of promoter-binding proteins of the fbp A and C genes in Mycobacterium tuberculosis. Tuberculosis (Edinb) 90: 25-30.

18. Aoshi T, Nagata T, Suzuki M, Uchijima M, Hashimoto D, et al. (2008) Identification of an HLA-A*0201-restricted T-cell epitope on the MPT51 protein, a major secreted protein derived from Mycobacterium tuberculosis, by MPT51 overlapping peptide screening. Infect Immun 76: 1565-1571.

19. Miki K, Nagata T, Tanaka T, Kim YH, Uchijima M, et al. (2004) Induction of protective cellular immunity against Mycobacterium tuberculosis by recombinant attenuated self-destructing Listeria monocytogenes strains harboring eukaryotic expression plasmids for antigen 85 complex and MPB/MPT51. Infect Immun 72: 2014-2021.

20. Bethunaickan R, Baulard AR, Locht C, Raja A (2007) Antibody response in pulmonary tuberculosis against recombinant 27kDa (MPT51, Rv3803c) protein of Mycobacterium tuberculosis. Scand J Infect Dis 39: 867-874.

21. Basu SK, Kumar D, Ganguly N, Rao KV, Sharma P (2009) Mycobacterium tuberculosis secreted antigen (MTSA-10) inhibits macrophage response to lipopolysaccharide by redox regulation of phosphatases. Indian J Exp Biol 47: 505-519.

22. Wiker HG, Harboe M, Bennedsen J, Closs O (1988) The antigens of Mycobacterium tuberculosis, H37Rv, studied by crossed immunoelectrophoresis. Comparison with a reference system for Mycobacterium bovis, BCG. Scand J Immunol 27: 223-239.

23. de Souza GA, Wiker HG (2011) A proteomic view of mycobacteria. Proteomics 11: 3118-3127.

24. Ireton GC, Greenwald R, Liang H, Esfandiari J, Lyashchenko KP, et al. (2010) Identification of Mycobacterium tuberculosis antigens of high serodiagnostic value. Clin Vaccine Immunol 17: 1539-1547.

25. Jindal S, Dudani AK, Singh B, Harley CB, Gupta RS (1989) Primary structure of a human mitochondrial protein homologous to the bacterial and plant chaperonins and to the 65-kilodalton mycobacterial antigen. Mol Cell Biol 9: 2279-2283.

26. Gupta RS (1990) Sequence and structural homology between a mouse T-complex protein TCP-1 and the 'chaperonin' family of bacterial (GroEL, 60-65 kDa heat shock antigen) and eukaryotic proteins. Biochem Int 20: 833-841.

27. Viale AM, Arakaki AK, Soncini FC, Ferreyra RG (1994) Evolutionary relationships among eubacterial groups as inferred from GroEL (chaperonin) sequence comparisons. Int J Syst Bacteriol 44: 527-533.

28. Baird PN, Hall LM, Coates AR (1988) A major antigen from Mycobacterium tuberculosis which is homologous to the heat shock proteins groES from E. coli and the htpA gene product of Coxiella burneti. Nucleic Acids Res 16: 9047.

29. Cobb AJ, Frothingham R (1999) The GroES antigens of Mycobacterium avium and Mycobacterium paratuberculosis. Vet Microbiol 67: 31-35.

30. Hu Y, Movahedzadeh F, Stoker NG, Coates AR (2006) Deletion of the Mycobacterium tuberculosis alpha-crystallin-like hspX gene causes increased bacterial growth in vivo. Infect Immun 74: 861-868.

31. Mattow J, Schaible UE, Schmidt F, Hagens K, Siejak F, et al. (2003) Comparative proteome analysis of culture supernatant proteins from virulent Mycobacterium tuberculosis H37Rv and attenuated M. bovis BCG Copenhagen. Electrophoresis 24: 3405-3420.

32. Stewart GR, Wernisch L, Stabler R, Mangan JA, Hinds J, et al. (2002) Dissection of the heat-shock response in Mycobacterium tuberculosis using mutants and microarrays. Microbiology 148: 3129-3138.

33. Li Y, Zeng J, Shi J, Wang M, Rao M, et al. (2010) A proteome-scale identification of novel antigenic proteins in Mycobacterium tuberculosis toward diagnostic and vaccine development. J Proteome Res 9: 4812-4822.

34. Chen D, Lei L, Lu C, Flores R, DeLisa MP, et al. (2010) Secretion of the chlamydial virulence factor CPAF requires the Sec-dependent pathway. Microbiology 156: 3031-3040.

35. Mastrangelo IA, Hough PV, Wall JS, Dodson M, Dean FB, et al. (1989) ATP-dependent assembly of double hexamers of SV40 T antigen at the viral origin of DNA replication. Nature 338: 658-662.

36. Chong JP, Hayashi MK, Simon MN, Xu RM, Stillman B (2000) A double-hexamer archaeal minichromosome maintenance protein is an ATP-dependent DNA helicase. Proc Natl Acad Sci U S A 97: 1530-1535.

37. Stahl H, Droge P, Knippers R (1986) DNA helicase activity of SV40 large tumor antigen. EMBO J 5: 1939-1944.

38. Al-Attiyah R, Mustafa AS (2004) Computer-assisted prediction of HLA-DR binding and experimental analysis for human promiscuous Th1-cell peptides in the 24 kDa secreted lipoprotein (LppX) of Mycobacterium tuberculosis. Scand J Immunol 59: 16-24.

39. Lefevre P, Denis O, De Wit L, Tanghe A, Vandenbussche P, et al. (2000) Cloning of the gene encoding a 22-kilodalton cell surface antigen of Mycobacterium bovis BCG and analysis of its potential for DNA vaccination against tuberculosis. Infect Immun 68: 1040-1047.

40. Yang B, Sayers S, Xiang Z, He Y (2011) Protegen: a web-based protective antigen database and analysis system. Nucleic Acids Res 39: D1073-1078.

41. Gehring AJ, Dobos KM, Belisle JT, Harding CV, Boom WH (2004) Mycobacterium tuberculosis LprG (Rv1411c): a novel TLR-2 ligand that inhibits human macrophage class II MHC antigen processing. J Immunol 173: 2660-2668.

42. Rezwan M, Grau T, Tschumi A, Sander P (2007) Lipoprotein synthesis in mycobacteria. Microbiology 153: 652-658.

43. Herrmann JL, Delahay R, Gallagher A, Robertson B, Young D (2000) Analysis of post-translational modification of mycobacterial proteins using a cassette expression system. FEBS Lett 473: 358-362.

44. Zhang F, Xie JP (2011) Mammalian cell entry gene family of Mycobacterium tuberculosis. Mol Cell Biochem 352: 1-10.

45. Chang ST, Linderman JJ, Kirschner DE (2008) Effect of multiple genetic polymorphisms on antigen presentation and susceptibility to Mycobacterium tuberculosis infection. Infect Immun 76: 3221-3232.

46. Malen H, Softeland T, Wiker HG (2008) Antigen analysis of Mycobacterium tuberculosis H37Rv culture filtrate proteins. Scand J Immunol 67: 245-252.

47. Lyashchenko K, Colangeli R, Houde M, Al Jahdali H, Menzies D, et al. (1998) Heterogeneous antibody responses in tuberculosis. Infect Immun 66: 3936-3940.

48. McShane H, Brookes R, Gilbert SC, Hill AV (2001) Enhanced immunogenicity of CD4(+) t-cell responses and protective efficacy of a DNA-modified vaccinia virus Ankara prime-boost vaccination regimen for murine tuberculosis. Infect Immun 69: 681-686.

49. Matsumoto S, Matsuo T, Ohara N, Hotokezaka H, Naito M, et al. (1995) Cloning and sequencing of a unique antigen MPT70 from Mycobacterium tuberculosis H37Rv and expression in BCG using E. coli-mycobacteria shuttle vector. Scand J Immunol 41: 281-287.

50. Betts JC, Lukey PT, Robb LC, McAdam RA, Duncan K (2002) Evaluation of a nutrient starvation model of Mycobacterium tuberculosis persistence by gene and protein expression profiling. Mol Microbiol 43: 717-731.

51. Lyashchenko KP, Pollock JM, Colangeli R, Gennaro ML (1998) Diversity of antigen recognition by serum antibodies in experimental bovine tuberculosis. Infect Immun 66: 5344-5349.

52. Gonzalez-Zamorano M, Mendoza-Hernandez G, Xolalpa W, Parada C, Vallecillo AJ, et al. (2009) Mycobacterium tuberculosis glycoproteomics based on ConA-lectin affinity capture of mannosylated proteins. J Proteome Res 8: 721-733.

53. Banu S, Honore N, Saint-Joanis B, Philpott D, Prevost MC, et al. (2002) Are the PE-PGRS proteins of Mycobacterium tuberculosis variable surface antigens? Mol Microbiol 44: 9-19.

54. Skeiky YA, Lodes MJ, Guderian JA, Mohamath R, Bement T, et al. (1999) Cloning, expression, and immunological evaluation of two putative secreted serine protease antigens of Mycobacterium tuberculosis. Infect Immun 67: 3998-4007.

55. Monod M, Jaton-Ogay K, Reichard U (1999) Aspergillus fumigatus-secreted proteases as antigenic molecules and virulence factors. Contrib Microbiol 2: 182-192.

56. Shin AR, Kim HJ, Cho SN, Collins MT, Manning EJ, et al. (2010) Identification of seroreactive proteins in the culture filtrate antigen of Mycobacterium avium ssp. paratuberculosis human isolates to sera from Crohn's disease patients. FEMS Immunol Med Microbiol 58: 128-137.

57. Diaz M, Esteban A, Fernandez-Abalos JM, Santamaria RI (2005) The high-affinity phosphate-binding protein PstS is accumulated under high fructose concentrations and mutation of the corresponding gene affects differentiation in Streptomyces lividans. Microbiology 151: 2583-2592.

58. Kadonaga JT (2004) Regulation of RNA polymerase II transcription by sequence-specific DNA binding factors. Cell 116: 247-257.

59. Willemsen PT, Westerveen J, Dinkla A, Bakker D, van Zijderveld FG, et al. (2006) Secreted antigens of Mycobacterium avium subspecies paratuberculosis as prominent immune targets. Vet Microbiol 114: 337-344.

60. Tummuru MK, Cover TL, Blaser MJ (1993) Cloning and expression of a high-molecular-mass major antigen of Helicobacter pylori: evidence of linkage to cytotoxin production. Infect Immun 61: 1799-1809.

61. Ticehurst JR, Aird DZ, Dam LM, Borek AP, Hargrove JT, et al. (2006) Effective detection of toxigenic Clostridium difficile by a two-step algorithm including tests for antigen and cytotoxin. J Clin Microbiol 44: 1145-1149.

62. Ben Amor Y, Shashkina E, Johnson S, Bifani PJ, Kurepina N, et al. (2005) Immunological characterization of novel secreted antigens of Mycobacterium tuberculosis. Scand J Immunol 61: 139-146.

63. Ford MJ, Anton IA, Lane DP (1988) Nuclear protein with sequence homology to translation initiation factor eIF-4A. Nature 332: 736-738.
