# Supplementary material for: Genome Sequencing and Analysis of BCG Vaccine Strains
Source: PLoS One. 2013 Aug 19;8(8):e71243. doi: 10.1371/journal.pone.0071243 (PMC3747166; doi:10.1371/journal.pone.0071243)
Supplement: Table S7 — Genes with Group 2–4 epitopes in H37Rv. “+”: epitopes in this gene exist in this BCG strain; “−”: epitopes in this gene do not exist in this BCG strain. “+/−”: there are several epitopes in this gene, and some were lost in this strain. “NA” in column 4 denotes that there are no related references to support that this gene is the antigen. (DOC) [file pone.0071243.s007.doc]

**Table S7. Genes owning group 2~group4 epitopes in H37Rv. “+” represents that epitopes in this gene are existing in this BCG strain, while “-” not. “+/-” represents that there are several epitopes in this gene and some of epitopes were lost in this strain. “NA” in column 4 is that no related reference to support that this gene is the antigen.**

| Group | Gene | Protein | Ref. | Epitopes Num |  |  | BCG | | | | | | | | | | | |
| --- | --- | --- | --- | --- | --- | --- | --- | --- | --- | --- | --- | --- | --- | --- | --- | --- | --- | --- |
|  |  |  |  |  | M. bovis | Prague | Sweden | Frappier | Glaxo | Moreau | Phipps | China | Danish | Russia | Tice | Mexico | Pasteur | Tokyo |
| 2 | *cyp130* | Cytochrome P450 130 CYP130 | NA | 1 | + | - | - | - | - | - | - | - | - | - | - | - | - | - |
| 2 | *echA1* | Enoyl-CoA hydratase |  | 3 | + | - | - | - | - | - | - | - | - | - | - | - | - | - |
| 2 | *esxA* | 6 kDa early Secretory antigenic target ESXA(ESAT-6) |  | 49 | + | - | - | - | - | - | - | - | - | - | - | - | - | - |
| 2 | *esxB* | 10 kDa culture filtrate antigen EsxB (CFP-10) |  | 56 | + | - | - | - | - | - | - | - | - | - | - | - | - | - |
| 2 | *Rv3878* | Unkonwn |  | 11 | + | - | - | - | - | - | - | - | - | - | - | - | - | - |
| 2 | *Rv1977* | Unkonwn | NA | 3 | + | - | - | - | - | - | - | - | - | - | - | - | - | - |
| 2 | *Rv3879c* | Unkonwn | NA | 1 | + | - | - | - | - | - | - | - | - | - | - | - | - | - |
| 3 | *mpt64* | Immunogenic protein MPT64 (antigen MPT64) |  | 23 | + | - | + | - | - | + | - | - | - | + | - | - | - | + |
| 3 | *Rv1985c* | Chromosome replication initiation inhibitorprotein | NA | 3 | + | - | + | - | - | + | - | - | - | + | - | - | - | + |
| 3 | *Rv1986* | Integral membrane protein |  | 1 | + | - | + | - | - | + | - | - | - | + | - | - | - | + |
| 3 | *Rv1987* | Chitinase |  | 1 | + | - | + | - | - | + | - | - | - | + | - | - | - | + |
| 4 | *esxL* | Putative ESAT-6 like protein ESXL |  | 1 | + | + | + | + | + | + | - | + | + | + | + | + | + | + |
| 4 | *esxN* | Putative ESAT-6 like protein ESXN |  | 7 | + | +/- | - | + | + | + | - | + | + | + | + | + | + | + |
| 4 | *esxO* | Putative ESAT-6 like protein ESXO |  | 2 | + | - | +/- | + | + | - | - | +/- | + | +/- | +/- | + | + | + |
| 4 | *esxQ* | Putative ESAT-6 like protein ESXQ |  | 7 | + | + | + | + | + | - | +/- | + | +/- | + | + | + | + | + |
| 4 | *esxR* | Putative ESAT-6 like protein ESXR |  | 7 | + | + | + | + | + | + | + | - | + | + | + | + | + | + |
| 4 | *mpt70* | Major Secreted immunogenic protein MPT70 |  | 2 | + | - | + | + | + | + | + | + | + | + | + | + | + | + |
| 4 | *PE_PGRS62* | PE-PGRS family protein |  | 1 | + | - | - | + | + | + | - | + | + | + | + | + | + | + |
| 4 | *pstS1* | Periplasmic phosphate-binding lipoprotein PSTS1(PBP-1) (PSTS1) |  | 4 | + | - | +/- | + | + | + | + | + | + | + | + | + | + | + |
| 4 | *Rv1158c* | Unkonwn | NA | 1 | + | + | + | + | + | + | - | + | + | + | + | + | + | + |
| 4 | *Rv0309* | Unkonwn | NA | 2 | + | + | + | - | + | + | + | + | + | + | + | + | + | + |
| 4 | *Rv2666* | Truncated IS1081 transposase | NA | 1 | + | - | + | + | - | + | - | - | - | - | - | + | + | + |
| 4 | *Rv3871* | Unkonwn | NA | 1 | + | + | - | - | + | - | - | - | + | - | - | + | + | + |

Reference：

1. Reddy M, Qreshi S, Hollenberg P, Reddy J (1981) Immunochemical identity of peroxisomal enoyl-CoA hydratase with the peroxisome-proliferation -associated 80,000 mol wt polypeptide in rat liver. The Journal of Cell Biology 89: 406-417.

2. Meher AK, Lella RK, Sharma C, Arora A (2007) Analysis of complex formation and immune response of CFP-10 and ESAT-6 mutants. Vaccine 25: 6098-6106.

3. Brodin P, Rosenkrands I, Andersen P, Cole ST, Brosch R (2004) ESAT-6 proteins: protective antigens and virulence factors? Trends in Microbiology 12: 500-508.

4. Agger EM, Brock I, Okkels LM, Arend SM, Aagaard CS, et al. (2003) Human T-cell responses to the RD1-encoded protein TB27.4 (Rv3878) from Mycobacterium tuberculosis. Immunology 110: 507-512.

5. Oettinger T, Holm A, HaslØV K (1997) Characterization of the Delayed Type Hypersensitivity-Inducing Epitope of MPT64 from Mycobacterium tuberculosis. Scandinavian Journal of Immunology 45: 499-503.

6. Yokoyama WM, Jacobs LB, Kanagawa O, Shevach EM, Cohen DI (1989) A murine T lymphocyte antigen belongs to a supergene family of type II integral membrane proteins. The Journal of Immunology 143: 1379-1386.

7. Agre P, Saboori AM, Asimos A, Smith BL (1987) Purification and partial characterization of the Mr 30,000 integral membrane protein associated with the erythrocyte Rh(D) antigen. Journal of Biological Chemistry 262: 17497-17503.

8. Adam R, Kaltmann B, Rudin W, Friedrich T, Marti T, et al. (1996) Identification of Chitinase as the Immunodominant Filarial Antigen Recognized by Sera of Vaccinated Rodents. Journal of Biological Chemistry 271: 1441-1447.

9. Johnson SM, Pappagianis D (1992) The coccidioidal complement fixation and immunodiffusion-complement fixation antigen is a chitinase. Infection and Immunity 60: 2588-2592.

10. Jones GJ, Gordon SV, Hewinson RG, Vordermeier HM (2010) Screening of Predicted Secreted Antigens from Mycobacterium bovis Reveals the Immunodominance of the ESAT-6 Protein Family. Infection and Immunity 78: 1326-1332.

11. Skjøt RLV, Brock I, Arend SM, Munk ME, Theisen M, et al. (2002) Epitope Mapping of the Immunodominant Antigen TB10.4 and the Two Homologous Proteins TB10.3 and TB12.9, Which Constitute a Subfamily of the esat-6 Gene Family. Infection and Immunity 70: 5446-5453.

12. Lyashchenko KP, Singh M, Colangeli R, Gennaro ML (2000) A multi-antigen print immunoassay for the development of serological diagnosis of infectious diseases. Journal of Immunological Methods 242: 91-100.

13. Lyashchenko KP, Pollock JM, Colangeli R, Gennaro ML (1998) Diversity of Antigen Recognition by Serum Antibodies in Experimental Bovine Tuberculosis. Infection and Immunity 66: 5344-5349.

14. Banu S, Honoré N, Saint-Joanis B, Philpott D, Prévost M-C, et al. (2002) Are the PE-PGRS proteins of Mycobacterium tuberculosis variable surface antigens? Molecular Microbiology 44: 9-19.

15. Målen H, Søfteland T, Wiker HG (2008) Antigen Analysis of Mycobacterium tuberculosis H37Rv Culture Filtrate Proteins. Scandinavian Journal of Immunology 67: 245-252.
