# Supplementary material for: Genome Sequencing and Analysis of BCG Vaccine Strains
Source: PLoS One. 2013 Aug 19;8(8):e71243. doi: 10.1371/journal.pone.0071243 (PMC3747166; doi:10.1371/journal.pone.0071243)
Supplement: Table S8 — Presence/absence genes in 16 strains of Mycobacterium tuberculosis (CDC1551, H37Rv, H37Ra, RGTB423, 7199-99, ATCC35801, NITR203, RGTB327, F11, UT205, CCDC5079, KZN1435, KZN4207, KZN605 and CTRI-2). Whole genome sequences of these strains were downloaded from the NCBI database. “+”: this gene exists; “−”: this gene was lost. (DOC) [file pone.0071243.s008.doc]

**Table S8. PA Genes in 16 strains of *M. tb* (CDC1551, H37Rv, H37Ra, RGTB423, 7199-99, ATCC35801, NITR203, RGTB327, F11, UT205, CCDC5079, KZN1435, KZN4207, KZN605 and CTRI-2)*.* Whole genome sequences of these strains were downloaded from NCBI. “+” represents the existence of this gene while “-” means the lost.**

| RD | Gene | AF2122_97 | CDC1551 | H37Rv | H37Ra | RGTB423 | 7199-99 | ATCC35801 | NITR203 | RGTB327 | F11 | UT205 | CCDC5079 | CCDC5180 | KZN1435 | KZN4207 | KZN605 | CTRI-2 |
| --- | --- | --- | --- | --- | --- | --- | --- | --- | --- | --- | --- | --- | --- | --- | --- | --- | --- | --- |
| 1 | Mb0072 | + | + | + | + | + | + | + | + | + | + | + | - | - | + | + | + | + |
|  | Mb0073 | + | + | + | + | + | + | + | + | + | + | + | - | - | + | + | + | + |
|  | Mb0074 | + | + | + | + | + | + | + | + | + | + | + | - | - | + | + | + | + |
| 2 | Mb0097c | + | + | + | + | + | + | + | + | + | + | - | + | + | + | + | + | + |
| 3 | Mb0286c | + | + | + | + | + | + | + | + | + | + | - | + | - | + | + | + | + |
| 4 | Mb0383c | + | + | + | + | + | + | + | + | + | + | + | + | + | - | - | - | - |
|  | Mb0384 | + | + | + | + | + | + | + | + | + | + | + | + | + | - | - | - | - |
|  | Mb0385 | + | + | + | + | + | + | + | + | + | + | + | + | + | - | - | - | - |
| 5 | Mb0545 | + | + | + | + | + | + | + | + | + | + | - | + | + | + | + | + | + |
| 6 | Mb0768 | + | + | + | + | + | + | - | + | + | + | - | + | + | + | + | + | + |
| 7 | Mb0817 | + | - | + | + | + | + | + | + | + | + | + | + | + | + | + | + | + |
|  | Mb0818c | + | - | + | + | + | + | + | + | + | + | + | + | + | + | + | + | + |
| 8 | Mb0837c | + | + | + | + | + | + | + | + | + | + | - | + | + | + | + | + | + |
|  | Mb0838c | + | + | + | + | + | + | + | + | + | + | - | + | + | + | + | + | + |
| 9 | Mb0856 | + | + | + | + | + | + | - | + | + | + | - | + | + | + | + | + | + |
| 10 | Mb1071c | + | + | + | + | + | + | + | + | + | + | - | + | + | + | + | + | + |
| 11 | Mb1076 | + | + | + | + | + | + | + | + | + | + | - | + | + | + | + | + | + |
| 12 | Mb1096c | + | + | + | + | + | + | + | + | + | + | - | + | + | + | + | + | + |
|  | Mb1097c | + | + | + | + | - | + | + | + | + | + | - | + | + | + | + | + | + |
| 13 | Mb1116 | + | + | + | + | + | + | + | + | + | + | - | + | - | + | + | + | + |
| 14 | Mb1121 | + | + | + | + | + | + | + | + | + | + | - | + | + | + | + | + | + |
| 15 | Mb1180 | + | + | + | + | + | + | + | + | + | + | - | + | + | + | + | + | + |
| 16 | Mb1231c | + | + | + | + | + | + | + | + | + | + | - | + | + | + | + | + | + |
| 17 | Mb1345c | + | + | + | + | + | + | + | + | + | + | - | + | + | + | + | + | + |
|  | Mb1346c | + | + | + | + | + | + | + | + | + | + | - | + | + | + | + | + | + |
| 18 | Mb1369 | + | + | + | + | + | + | + | + | + | - | + | + | + | + | + | + | + |
|  | Mb1370 | + | + | + | + | + | + | + | + | + | - | + | + | + | + | + | + | + |
|  | Mb1371 | + | + | + | + | + | + | + | + | + | - | + | + | + | + | + | + | + |
| 19 | Mb1389c | + | + | + | + | + | + | - | + | + | + | + | + | + | + | + | + | + |
|  | Mb1390c | + | + | + | + | + | + | - | + | + | + | + | + | + | + | + | + | + |
|  | Mb1391c | + | + | + | + | + | + | - | + | + | + | + | + | + | + | + | + | + |
| 20 | Mb1485c | + | + | + | + | + | + | + | + | + | + | - | + | + | + | + | + | + |
| 21 | Mb1551 | + | + | + | + | - | + | + | + | + | + | + | + | + | + | + | + | + |
|  | Mb1552 | + | + | + | + | - | + | + | + | + | + | + | + | + | + | + | + | + |
| 22 | Mb1582 | + | - | - | - | - | - | - | - | - | - | - | - | - | - | - | - | - |
|  | Mb1583 | + | - | - | - | - | - | - | - | - | - | - | - | - | - | - | - | - |
| 23 | Mb1599 | + | + | + | + | + | + | + | + | + | - | - | - | - | - | - | - | - |
|  | Mb1600 | + | + | + | + | + | + | + | + | - | - | - | - | - | - | - | - | - |
|  | Mb1601 | + | + | + | + | + | + | + | + | - | - | - | - | - | - | - | - | - |
|  | Mb1602c | + | + | + | + | + | + | + | + | + | - | - | - | - | - | - | - | - |
|  | Mb1603c | + | + | + | + | + | + | + | + | + | + | - | - | + | + | + | + | + |
|  | Mb1604c | + | + | + | + | + | + | + | + | - | - | - | - | - | - | - | - | - |
|  | Mb1605c | + | + | + | + | + | + | + | + | - | - | - | - | - | - | - | - | - |
|  | Mb1606c | + | + | + | + | + | + | + | + | - | - | - | - | - | - | - | - | - |
|  | Mb1607c | + | + | + | + | + | + | + | + | - | - | - | - | - | - | - | - | - |
|  | Mb1608c | + | + | + | + | + | + | + | + | - | - | - | - | - | - | - | - | - |
|  | Mb1609c | + | + | + | + | + | + | + | + | - | - | - | - | - | - | - | - | - |
|  | Mb1610c | + | + | + | + | + | + | + | + | - | - | - | - | - | - | - | - | - |
|  | Mb1611c | + | + | + | + | + | + | + | + | - | - | - | - | - | - | - | - | - |
|  | Mb1612c | + | + | + | + | + | + | + | + | - | - | - | - | - | - | - | - | - |
|  | Mb1613c | + | + | + | + | + | + | + | + | + | + | - | + | + | + | + | + | + |
|  | Mb1614c | + | + | + | + | + | + | + | + | + | + | - | + | + | + | + | + | + |
| 24 | Mb1784c | + | + | + | + | + | + | + | + | + | + | - | - | - | - | - | - | - |
|  | Mb1785c | + | + | - | + | - | + | + | - | - | + | - | - | - | - | - | - | - |
|  | Mb1786 | + | + | - | + | - | + | + | - | - | + | - | - | - | - | - | - | - |
|  | Mb1787 | + | + | - | + | - | + | + | - | - | + | - | - | - | - | - | - | - |
|  | Mb1788 | + | + | + | + | + | + | + | + | + | + | - | - | - | + | + | + | + |
|  | Mb1789c | + | + | + | + | + | + | + | + | + | + | - | - | - | + | + | + | + |
|  | Mb1790c | + | + | + | + | + | + | + | + | + | + | - | - | - | + | + | + | + |
|  | Mb1791 | + | + | + | + | + | + | + | + | + | + | - | - | - | + | + | + | + |
|  | Mb1792c | + | + | + | + | + | + | + | + | + | + | - | - | - | + | + | + | + |
|  | Mb1793c | + | + | + | + | + | + | + | + | + | + | - | - | - | + | + | + | + |
| 25 | Mb1815 | + | + | + | + | + | + | + | + | + | + | - | + | + | + | + | + | + |
| 26 | Mb1818 | + | + | + | + | + | + | + | + | + | + | - | + | + | + | + | + | + |
| 27 | Mb2016c | + | + | + | + | + | + | + | + | + | + | - | + | + | + | + | + | + |
|  | Mb2017c | + | + | + | + | + | + | + | + | + | + | - | + | + | + | + | + | + |
|  | Mb2018 | + | + | + | + | + | + | + | + | - | + | - | + | + | + | + | + | + |
|  | Mb2019 | + | + | + | + | + | + | + | + | - | + | - | + | + | + | + | + | + |
| 28 | Mb2048c | + | + | - | - | - | + | + | - | - | + | + | + | + | + | + | + | + |
|  | Mb2049c | + | + | - | - | - | + | + | - | - | + | + | + | + | + | + | + | + |
| 29 | Mb2074c | + | + | + | + | + | + | + | + | + | + | + | - | + | + | + | + | + |
| 30 | Mb2127 | + | + | + | + | + | - | - | + | + | + | + | + | + | + | + | + | + |
|  | Mb2128 | + | + | + | + | + | - | - | + | + | + | + | + | + | + | + | + | + |
| 31 | Mb2131 | + | + | + | + | + | + | + | + | + | - | + | + | + | + | + | + | + |
| 32 | Mb2147 | + | - | + | + | + | + | + | + | + | + | + | + | + | + | + | + | + |
| 33 | Mb2199c | + | + | + | + | + | + | + | + | + | + | - | + | + | + | + | + | + |
| 34 | Mb2286 | + | + | + | + | + | + | + | + | + | + | + | - | - | + | + | + | + |
| 35 | Mb2294 | + | + | + | + | + | - | - | + | + | + | + | + | + | + | + | + | + |
|  | Mb2295 | + | + | + | + | + | - | - | + | + | + | + | + | + | + | + | + | + |
|  | Mb2296 | + | + | + | + | + | - | - | + | + | + | + | + | + | + | + | + | + |
|  | Mb2297c | + | + | + | + | + | - | - | + | + | + | + | + | + | + | + | + | + |
|  | Mb2298 | + | + | + | + | + | - | - | + | + | + | + | + | + | + | + | + | + |
|  | Mb2299 | + | + | + | + | + | - | - | + | + | + | + | + | + | + | + | + | + |
|  | Mb2300c | + | + | + | + | + | - | - | + | + | + | + | + | + | + | + | + | + |
| 36 | Mb2377c | + | + | + | + | + | + | + | + | + | + | + | - | + | + | + | + | - |
| 37 | Mb2447c | + | + | + | + | + | + | + | + | + | + | - | + | + | + | + | + | + |
| 38 | Mb2540c | + | + | + | + | + | + | + | + | + | + | - | + | + | + | + | + | + |
|  | Mb2541 | + | + | + | + | + | + | + | + | + | + | - | + | + | + | + | + | + |
| 39 | Mb2685 | + | + | + | + | + | + | + | + | + | + | - | + | + | + | + | + | + |
| 40 | Mb2838c | + | + | + | + | + | + | + | + | + | + | - | + | + | + | + | + | + |
|  | Mb2839c | + | + | + | + | + | + | + | + | + | + | - | + | + | + | + | + | + |
|  | Mb2840c | + | + | + | + | + | + | + | + | + | + | + | - | - | + | + | + | + |
|  | Mb2841c | + | + | + | + | + | + | + | + | + | + | + | - | - | + | + | + | + |
|  | Mb2842c | + | + | + | + | + | + | - | + | + | + | + | - | - | + | + | + | + |
|  | Mb2843c | + | + | + | + | + | + | + | + | + | + | + | - | - | + | + | + | + |
|  | Mb2844c | + | + | + | + | + | + | + | + | + | + | + | - | - | + | + | + | + |
|  | Mb3045c | + | + | + | + | + | - | - | + | + | + | + | - | + | + | + | + | + |
|  | Mb3046c | + | + | + | + | + | - | - | + | + | + | + | + | + | + | + | + | + |
| 41 | Mb3049c | + | + | + | + | + | + | + | + | + | + | - | + | + | + | + | + | + |
| 42 | Mb3112 | + | + | + | + | + | + | + | + | + | + | + | + | + | + | + | + | - |
| 43 | Mb3140 | + | + | + | + | + | + | + | + | + | + | + | + | + | - | - | - | + |
| 44 | Mb3142 | + | + | + | + | + | + | + | + | + | + | - | + | + | + | + | + | + |
| 45 | Mb3144 | + | + | + | + | + | + | + | + | + | + | - | + | + | + | + | + | + |
| 46 | Mb3159 | + | - | - | - | - | - | - | - | - | - | - | - | - | - | - | - | - |
| 47 | Mb3184c | + | + | - | - | - | + | + | - | - | - | - | + | + | - | - | - | - |
| 48 | Mb3352c | + | + | + | + | + | + | - | + | + | + | + | + | + | + | + | + | + |
|  | Mb3353c | + | + | + | + | + | + | - | + | + | + | + | + | + | + | + | + | + |
|  | Mb3354c | + | + | - | - | - | + | - | - | - | + | + | + | + | + | + | + | + |
|  | Mb3355c | + | + | - | - | - | + | - | - | - | - | - | + | + | - | - | - | + |
|  | Mb3356 | + | + | - | - | - | + | - | - | - | - | - | + | + | - | - | - | + |
|  | Mb3357 | + | + | - | - | - | + | - | - | - | - | - | + | + | - | - | - | + |
|  | Mb3358 | + | + | - | - | - | + | - | - | - | - | - | + | + | - | - | - | + |
|  | Mb3359c | + | + | - | - | - | + | - | - | - | - | - | - | - | - | - | - | + |
| 49 | Mb3361c | + | + | + | + | + | + | + | + | + | + | + | + | + | - | - | - | + |
| 50 | Mb3375c | + | - | + | + | + | + | + | + | + | + | + | + | + | + | + | + | + |
| 51 | Mb3380c | + | + | + | + | + | + | + | + | - | + | - | + | + | + | + | + | + |
| 52 | Mb3382c | + | + | + | + | + | + | + | + | + | + | - | + | + | + | + | + | + |
| 53 | Mb3385c | + | + | + | + | + | + | + | + | - | + | - | + | + | + | + | + | + |
|  | Mb3386c | + | + | + | + | + | + | + | + | - | + | - | + | + | + | + | + | + |
|  | Mb3387c | + | + | + | + | + | + | + | + | - | + | - | + | + | + | + | + | + |
|  | Mb3388c | + | + | + | + | + | + | + | + | - | + | - | + | + | + | + | + | + |
|  | Mb3389 | + | + | + | + | + | + | + | + | + | + | - | + | + | + | + | + | + |
| 54 | Mb3420 | + | + | + | + | + | + | + | + | + | + | - | + | + | + | + | + | + |
| 55 | Mb3459 | + | - | + | + | + | + | + | + | + | - | - | + | + | - | - | - | - |
| 56 | Mb3495 | + | + | + | + | + | + | + | + | + | + | - | + | + | + | + | + | + |
|  | Mb3496 | + | + | + | + | + | + | + | + | + | + | - | + | + | + | + | + | + |
| 57 | Mb3537 | + | + | + | + | + | + | + | + | + | + | - | + | + | + | + | + | + |
|  | Mb3538 | + | + | + | + | + | + | + | + | + | + | - | + | + | + | + | + | + |
| 58 | Mb3541 | + | + | + | + | + | + | - | + | + | + | - | + | + | + | + | + | + |
|  | Mb3543 | + | + | + | + | + | + | + | + | + | + | - | + | + | + | + | + | + |
| 59 | Mb3549 | + | - | + | + | + | + | + | + | + | + | + | + | + | + | + | + | + |
| 60 | Mb3641 | + | + | + | + | + | + | - | + | + | + | - | + | + | + | + | + | + |
| 61 | Mb3827 | + | + | + | + | + | + | + | + | + | + | - | + | + | + | + | + | + |
|  | Mb3828 | + | + | + | + | + | + | + | + | + | + | - | + | + | + | + | + | + |
| RD Num. | |  | 7 | 6 | 5 | 7 | 5 | 12 | 6 | 11 | 8 | 40 | 10 | 11 | 10 | 10 | 10 | 10 |
