# Supplementary material for: Genome Sequencing and Analysis of BCG Vaccine Strains
Source: PLoS One. 2013 Aug 19;8(8):e71243. doi: 10.1371/journal.pone.0071243 (PMC3747166; doi:10.1371/journal.pone.0071243)
Supplement: Table S9 — The presense of repetitive sequences in the 13 Bacillus Calmette-Guérin strains. (DOC) [file pone.0071243.s009.doc]

**Table S9.** The presense of repetitive sequences in 13 BCG strains.

| **Gene** | **Annotation** | **Frappier** | **Glaxo** | **Moreau** | **Phipps** | **Pragure** | **Sweden** | **Mexico** | **China** | **Danish** | **Russia** | **Tice** | **Pasteur** | **Tokyo** |
| --- | --- | --- | --- | --- | --- | --- | --- | --- | --- | --- | --- | --- | --- | --- |
| Mb0099 | ID=Mb0099; PPE1 Mb0099, PPE1, len: 463aa. Equivalent to Rv0096, len: 463 aa, fromMycobacterium tuberculosis strain H37Rv, (99.8%identity in 463 aa overlap). Member of the Mycobacterium tuberculosis PPE family, similar to many e.g. Z46257|MLACEA_3 aceA gene for isocitrate L from M. leprae (438 aa), FASTA scores: opt: 1207, E(): 0, (55.3% identity in 380 aa overlap). Also similar to Z97559|MTCY261_19 from Mycobacterium tuberculosis (473 aa), FASTA score: (40.2% identity in 478 aa overlap); YHS6_MYCTU|P42611 hypothetical 50.6 kd protein (517aa), FASTA scores: opt: 365, E(): 4.6e-12, (37.6%identity in 178 aa overlap). Also similar toMTCY274.23c from M. tuberculosis FASTA score:(31.1% identity in 383 overlap). Some similarityalso to MTCY31.06c and MTCY48.17 and other mycobacterial PPE family proteins.; complete gene | + | + | + | + | + | + | + | + | + | + | + | + | + |
| Mb0156c | ID=Mb0156c; PE1 Mb0156c, PE1, len: 588aa. Equivalent to Rv0151c, len: 588 aa, fromMycobacterium tuberculosis strain H37Rv, (99.8%identity in 588 aa overlap). Member of the Mycobacterium tuberculosis PE family, with N-terminal region similar to others e.g. MTV032_2 PE_PGRS family from Mycobacterium tuberculosis (468 aa), FASTA scores: opt: 1125, E(): 0, (46.3% identity in 456 aa overlap); MTCY493_24 from M. tuberculosis FASTA score: (42.5% identity in 558 aa overlap). Also similar to upstreamORF MTCI5.26c FASTA score: (54.7% identity in 464aa overlap). Also shows similarity to C-terminalpart of some PPE family proteins e.g. MTV049_21from Mycobacterium tuberculosis FASTA score:(41.5% identity in 591 aa overlap).; completegene | + | + | + | + | + | + | + | + | + | + | + | + | + |
| Mb0294 | ID=Mb0294; PPE4 Mb0294, PPE4, len: 513aa. Equivalent to Rv0286, len: 513 aa, fromMycobacterium tuberculosis strain H37Rv, (100.0%identity in 513 aa overlap). Member of the Mycobacterium tuberculosis PPE family, similar to others e.g. AL0212|MTV012_32 from Mycobacterium tuberculosis (434 aa), FASTA scores: opt: 958, E(): 0, (43.5% identity in 522 aa overlap).; complete gene | + | + | + | + | + | - | + | - | - | - | - | + | + |
| Mb0312c | ID=Mb0312c; PPE5 Mb0312c, PPE5, len:1147 aa. Equivalent to 3' end of Rv0304c, len:2204 aa, from Mycobacterium tuberculosis strainH37Rv, (99.9% identity in 1147 aa overlap). Member of the Mycobacterium tuberculosis PE family (PPE, MPTR), similar to others e.g. Z95324|MTY13E10_16 from M. tuberculosis (1443 aa), FASTA scores: E(): 0, (50.6% identity in 1403 aa overlap); Y04H_MYCTU|Q10778 from M. tuberculosis (734 aa), FASTA scores: opt: 989, E(): 0, (42.3% identity in 522 aa overlap). REMARK-M.bovis-M.tuberculosis: In Mycobacterium tuberculosis strain H37Rv, PPE5 and PPE6 exist as separate genes. In Mycobacterium bovis, a frameshift due to a single base deletion (g-*) leads to a shorter CDS (Mb0312c) equivalent to the 3' end of Rv0304c/PPE5.; complete gene | + | + | + | + | + | + | + | + | + | + | + | + | + |
| Mb0313c | ID=Mb0313c; PPE6 Mb0313c, PPE6, len:1985 aa. Equivalent to 5' end of Rv0305c, len: 963aa, from Mycobacterium tuberculosis strain H37Rv,(100.0% identity in 809 aa overlap). Member of theMycobacterium tuberculosis PE family (PPE, MPTR), similar to others e.g. Y04H_MYCTU|Q10778 from M. tuberculosis (734 aa), FASTA scores: opt: 1340, E(): 0, (40.9% identity in 815 aa overlap); Z95324|MTY13E10_16 from Mycobacterium tuberculosis (1443 aa), FASTA scores: E(): 0, (50.6% identity in 1403 aa overlap); Y04H_MYCTU|Q10778 from Mycobacterium tuberculosis (734 aa), FASTA scores: opt: 989, E(): 0, (42.3% identity in 522 aa overlap). REMARK-M.bovis-M.tuberculosis: In Mycobacterium tuberculosis strain H37Rv, PPE5 andPPE6 exist as separate genes. In Mycobacteriumbovis, a single base deletion (t-*) resulting inthe absence of a stop codon leads to a longerproduct. The second part of this CDS shareshomology with the 5' end of Rv0304c/PPE5.; complete gene | + | + | - | - | - | - | + | - | + | + | - | + | + |
| Mb0362c | ID=Mb0362c; PPE8 Mb0362c, PPE8, len:3507 aa. Equivalent to Rv0355c and Rv0354c, len:3300 aa and 141 aa, from Mycobacterium tuberculosis strain H37Rv, (99.8% identity in 3296 aa overlap and 100.0% identity in 125 aa overlap). PPE8, member of the Mycobacterium tuberculosis PPE family, similar to others e.g. AL009198|MTV004_5 from M. tuberculosis (3716 aa), FASTA scores: opt: 2906, E(): 0, (40.9% identity in 3833 aa overlap); MTV004_3 FASTA scores: (39.0% identity in 3531 aa overlap); etc. Gene contains large number of clustered Major Polymorphic Tandem Repeats (MPTR). Related to MTCY13E10.16c,E(): 0; MTCY13E10.17c, E(): 0; MTCY48.17, E(): 0;MTCY98.0034c, E(): 0; MTCY03C7.23 E(): 0;MTCY98.0031c, E(): 0; MTCY31.06c, E(): 5.6e-17;MTCY359.33, E(): 2.3e-16. PPE7, member of theMycobacterium tuberculosis PPE family, similar toothers e.g. MTCY63_9 from Mycobacterium tuberculosis (2411 aa), FASTA scores: E(): 3.6e-11, (47.6% identity in 103 aa overlap). Possible continuation of ORF upstream, but no sequence error apparent. REMARK-M.bovis-M.tuberculosis: In Mycobacterium tuberculosis strain H37Rv, PPE7 and PPE8 exist as 2 genes. In Mycobacterium bovis, a 2 bp insertion (*-ta) resulting in the absence of a stop codon between the 2 genes,leads to a single product.; complete gene | + | + | + | + | + | + | + | + | + | + | + | + | + |
| Mb0394c | ID=Mb0394c; PPE9 Mb0394c, PPE9, len: 443aa. Equivalent to Rv0388c and Rv0387c, len: 180 aaand 244 aa, from Mycobacterium tuberculosis strainH37Rv, (95.1% identity in 164 aa overlap and100.0% identity in 244 aa overlap). Rv0388c: Member of the Mycobacterium tuberculosis PPE family, highly similar to others e.g. MTCY10G2_10|Z92539 from Mycobacterium tuberculosis (391 aa), FASTA scores: opt: 667, E(): 0, (58.3% identity in 180 aa overlap) but much shorter. Rv0387c: conserved hypothetical protein,showing some similarity to MTCI237.20c, andM17282|HUMEL20_1 Human elastin gene, exon 1,Elastin (687 aa), FASTA scores: opt: 193, E():0.35, (34.4% identity in 189 aa overlap). REMARK-M.bovis-M.tuberculosis: In Mycobacterium tuberculosis strain H37Rv, Rv0388c and Rv0387c exist as 2 separate genes. In Mycobacterium bovis, 3 different base substitutions, the first of 14 bases, the second of 8 bases (tctacagt-gctacagg), and lastly of 28 bases,leads to a longer single product.; completegene | + | + | + | + | + | - | + | + | - | + | + | + | + |
| Mb0621 | ID=Mb0621; Mb0621, -, len: 202 aa.Equivalent to Rv0605, len: 202 aa, fromMycobacterium tuberculosis strain H37Rv, (100.0%identity in 202 aa overlap). Possible resolvase for IS_Y349 element, similar to several Mycobacterial hypothetical proteins and weakly similar to Q52563 resolvase from Pseudomonas syringae (210 aa), FASTA scores: opt: 99, E(): 3.1, (35.7% identity in 98 aa overlap). Contains PS00397 Site-specific recombinases active site and probable helix-turn helix motif from aa 9-30 (Score 1815, +5.37 SD).; complete gene | + | + | + | + | + | + | + | + | + | + | + | + | + |
| Mb0622 | ID=Mb0622; Mb0622, -, len: 247 aa.Equivalent to Rv0606, len: 247 aa, fromMycobacterium tuberculosis strain H37Rv, (100.0%identity in 247 aa overlap). Possible truncated transposase for IS_1536 element, highly similar to N-terminus of other transposases from Mycobacterium tuberculosis e.g. YX16_MYCTU|Q10809|Rv2885c|MT2953|MTCY274.16c PUTATIVE TRANSPOSASE from Mycobacterium tuberculosis (460 aa), FASTA scores: opt: 1368, E(): 0, (83.5% identity in 237 aa overlap); Rv2978c, Rv0922, Rv3827c, etc. Also similar to N-terminus of MTV002_57|Rv2792 RESOLVASE from M. tuberculosis (193 aa), FASTA score: (87.4% identityin 238 aa overlap).; complete gene | + | + | + | + | + | + | + | + | + | + | + | + | + |
| Mb0777c | ID=Mb0777c; PPE12 Mb0777c, PPE12, len:645 aa. Equivalent to Rv0755c, len: 645 aa, fromMycobacterium tuberculosis strain H37Rv, (99.8%identity in 645 aa overlap). Member of the Mycobacterium tuberculosis PPE family, highly similar to others e.g. Z82098|MTCY3C7_23 from Mycobacterium tuberculosis (582 aa), FASTA scores: (56.1% identity in 636 aa overlap); Z92774|MTCY6G11_5 from Mycobacterium tuberculosis (552 aa), FASTA scores: (55.8% identity in 590 aa overlap); etc.; complete gene | + | + | + | + | + | + | + | + | + | + | + | + | + |
| Mb0869c | ID=Mb0869c; Mb0869c, -, len: 504 aa.Equivalent to Rv0846c, len: 504 aa, fromMycobacterium tuberculosis strain H37Rv, (99.8%identity in 504 aa overlap). Probable oxidase (EC 1.-.-.-), showing similarity with several oxidases, mainly L-ascorbate oxidases and copper resistance proteins A (precursors) e.g. P24792|ASO_CUCMA L-ASCORBATE OXIDASE PRECURSOR (ASCORBASE) (EC 1.10.3.3) from Cucurbitamaxima (Pumpkin) (Winter squash) (579 aa), FASTAscores: opt: 423, E(): 5.8e-18, (28.4% identity in493 aa overlap); AF010496|AF010496_32 potentialmulticopper oxidase from Rhodobacter capsulatus(491 aa), FASTA scores: opt: 490, E(): 2.7e-22,(28.8% identity in 510 aa overlap); 47452|PCOA_ECOLI COPPER RESISTANCE PROTEIN A PRECURSOR (BELONGS TO THE FAMILY OF MULTICOPPER OXIDASES) from Escherichia coli strain K12 (605 aa); etc. Contains PS00080 Multicopper oxidases signature 2 at C-terminus. SEEMS TO BELONG TO THE FAMILY OF MULTICOPPER OXIDASES.; complete gene | + | + | + | + | + | + | + | + | + | + | + | + | + |
| Mb0902c | ID=Mb0902c; PPE13 Mb0902c, PPE13, len:438 aa. Equivalent to Rv0878c, len: 443 aa, fromMycobacterium tuberculosis strain H37Rv, (100.0%identity in 438 aa overlap). Member of the Mycobacterium tuberculosis PPE family, highly similar to many e.g. P4261|YHS6_MYCTU (517 aa), FASTA scores: opt: 1044, E(): 0, (47.4% identity in 397 aa overlap); MTV014_3, MTCI65_2, MTCY98_24, MTCY3C7_23, MTCY48_17, MTV004_5, MTV004_3, etc. REMARK-M.bovis-M.tuberculosis:In Mycobacterium bovis, a single base deletion(a-*) leads to a shorter product compared to itshomolog in Mycobacterium tuberculosis strain H37Rv(438 aa versus 443 aa).; complete gene | + | + | + | + | + | + | + | + | + | + | + | + | + |
| Mb1068c | ID=Mb1068c; PPE15 Mb1068c, PPE15, len:391 aa. Equivalent to Rv1039c, len: 391 aa, fromMycobacterium tuberculosis strain H37Rv, (100%identity in 391 aa overlap). Member of the Mycobacterium tuberculosis PPE family of glycine-rich proteins, most similar to Rv2768c|AL008967|MTV002_33 Mycobacterium tuberculosis H37Rv (394 aa), FASTA scores: opt: 1721, E(): 0, (70.4% identity in 398 aa overlap).; complete gene | + | + | + | + | + | - | + | + | + | + | + | + | + |
| Mb1076 | ID=Mb1076; Mb1076, -, len: 415 aa.Equivalent to Rv1047, len: 415 aa, fromMycobacterium tuberculosis strain H37Rv, (100%identity in 415 aa overlap). IS1081 transposase, most similar to TRA1_MYCBO|P35882 transposase for insertion sequence element (415 aa), FASTA scores: opt: 2675, E(): 0, (99.8% identity in 415 aa overlap). Contains PS01007 Transposases, Mutator family, signature; complete gene | - | - | - | - | - | - | + | - | - | - | - | + | + |
| Mb1166c | ID=Mb1166c; PPE16 Mb1166c, PPE16, len:618 aa. Equivalent to Rv1135c, len: 618 aa, fromMycobacterium tuberculosis strain H37Rv, (100%identity in 618 aa overlap). Member of the M. tuberculosis PPE family of glycine-rich proteins. Similar to Rv2356c (59.6% identity in 627 aa overlap); etc.; complete gene | - | + | - | + | - | - | + | - | - | - | - | + | + |
| Mb1201c | ID=Mb1201c; PPE17a Mb1201c, PPE17a,len: 180 aa. Similar to 5' end of Rv1168c, len:346 aa, from Mycobacterium tuberculosis strainH37Rv, (97.1% identity in 174 aa overlap). Member of the Mycobacterium tuberculosis PPE family of glycine-rich proteins, similar to many e.g. E332789|Z98268|MTCI125.27C (385 aa), FASTA scores: opt: 504, E(): 0, (36.6% identity in 388 aa overlap). REMARK-M.bovis-M.tuberculosis: In Mycobacterium tuberculosis strain H37Rv, PPE17 exists as a single gene. In Mycobacterium bovis, a frameshift due to a single base insertion (*-c) splits PPE17 into 2 parts, PPE17a and PPE17b.; complete gene | + | + | - | - | - | - | + | + | + | + | + | + | + |
| Mb1228 | ID=Mb1228; PPE18 Mb1228, PPE18, len:390 aa. Equivalent to Rv1196, len: 391 aa, fromMycobacterium tuberculosis strain H37Rv, (99.0%identity in 391 aa overlap). PPE18 (alternate gene name: mtb39a). Member of the Mycobacterium tuberculosis PPE family of glycine-rich proteins, highly similar to others e.g. Y07P_MYCTU|Q11031 hypothetical 40.0 kDa protein cy02b10.25c (396 aa), FASTA scores: opt: 2124, E(): 0, (85.1% identity in 397 aa overlap). Note that expression of Rv1196 was demonstrated in lysates by immunodetection (see first citation below). REMARK-M.bovis-M.tuberculosis: In Mycobacterium bovis, a 14 bp to 11 bp substitution leads to a slightlyshorter product compared to its homolog inMycobacterium tuberculosis strain H37Rv (390 aaversus 391 aa).; mtb39a; complete gene | - | - | - | - | + | - | + | - | - | + | - | + | + |
| Mb1231c | ID=Mb1231c; Mb1231c, -, len: 415 aa.Equivalent to Rv1199c, len: 415 aa, fromMycobacterium tuberculosis strain H37Rv, (100%identity in 415 aa overlap). Possible transposase for IS1081, identical to TRA1_MYCBO|P35882 transposase for insertion sequence element (415 aa); region identical to MTCY441.35 (100.0% identity in 261 aa overlap); andalmost identical to MTCY10G2.02c (415 aa) (99.8%identity in 415 aa overlap). Contains PS01007Transposases, Mutator family, signature, PS00435Peroxidases proximal heme-ligand signature.; complete gene | - | - | - | - | - | - | + | - | - | - | - | + | + |
| Mb1345c | ID=Mb1345c; Mb1345c, -, len: 243 aa.Equivalent to 3' end of Rv1313c, len: 444 aa, fromMycobacterium tuberculosis strain H37Rv, (100%identity in 243 aa overlap). Possible IS1557transposase, similar to several transposases e.g. U57649|DBU57649 ORF1 from dibenzofuran-degrading bacterium DPO360 (163 aa), FASTA scores: opt: 767, E(): 0, (67.3% identity in 168 aa overlap); TNPA_BORPA|Q06126transposase for insertion sequence element IS1001from Bordetella parapertussis (406 aa), FASTAscores: opt: 254, E(): 3.3e-10, (24.9% identity in402 aa overlap). Also similar to putativeMycobacterium tuberculosis transposases, Rv3798and Rv0741. REMARK-M.bovis-M.tuberculosis: In Mycobacterium tuberculosis strain H37Rv, Rv1313c exists as a single gene. In Mycobacterium bovis, a frameshift due to a 12 bp to 1 bp substitution (cttgtcgtggcc-t) splits Rv1313c into 2 parts, Mb1345c and Mb1346c.; complete gene | + | + | + | + | + | + | + | - | - | - | - | + | + |
| Mb1346c | ID=Mb1346c; Mb1346c, -, len: 205 aa.Equivalent to 5' end of Rv1313c, len: 444 aa, fromMycobacterium tuberculosis strain H37Rv, (99.5%identity in 195 aa overlap). Possible IS1557transposase, similar to several transposases e.g. U57649|DBU57649 ORF1 from dibenzofuran-degrading bacterium DPO360 (163 aa), FASTA scores: opt: 767, E(): 0, (67.3% identity in 168 aa overlap); TNPA_BORPA|Q06126transposase for insertion sequence element IS1001from Bordetella parapertussis (406 aa), FASTAscores: opt: 254, E(): 3.3e-10, (24.9% identity in402 aa overlap). Also similar to putativeMycobacterium tuberculosis transposases, Rv3798and Rv0741. REMARK-M.bovis-M.tuberculosis: In Mycobacterium tuberculosis strain H37Rv, Rv1313c exists as a single gene. In Mycobacterium bovis, a frameshift due to a 12 bp to 1 bp substitution (cttgtcgtggcc-t) splits Rv1313c into 2 parts, Mb1345c and Mb1346c.; complete gene | + | + | + | + | + | + | + | - | - | - | - | + | + |
| Mb1396c | ID=Mb1396c; PPE19 Mb1396c, PPE19, len:396 aa. Similar to Rv1361c, len: 396 aa, fromMycobacterium tuberculosis strain H37Rv, (92.9%identity in 396 aa overlap). PPE19 (alternate gene name: mtb39b). Member of the Mycobacterium tuberculosis PPE family of glycine-rich proteins, highly similar to many e.g. Rv1196|MTCI364.08|PPE18, FASTA scores: E(): 0, (84.9% identity in 397 aa overlap); MTCY274.23c (42.3% identity in 416 aa overlap); etc. Contains PS00501Signal peptidases I serine active site. Note thatexpression of Rv1361c was demonstrated in lysatesby immunodetection (see first citation below).REMARK-M.bovis-M.tuberculosis: In Mycobacteriumbovis, the PPE19 gene contains nine substitutionscompared to Mycobacterium tuberculosis strainH37Rv.; mtb39b; complete gene | - | - | - | - | + | - | + | - | - | + | - | + | + |
| Mb1575c | ID=Mb1575c; PPE21 Mb1575c, PPE21, len:678 aa. Equivalent to Rv1548c, len: 678 aa, fromMycobacterium tuberculosis strain H37Rv, (99.7%identity in 678 aa overlap). Member of the M. tuberculosis PPE family, similar to several e.g. YHS6_MYCTU|P42611 hypothetical 50.6 kd protein in hsp65 3' region (517 aa), FASTA scores: opt:1142, E(): 0, (40.6% identity in 616 aa overlap); also similar to MTCY31.06c (54.9% identity in 381 aa overlap).; completegene | + | + | + | + | + | + | + | + | + | + | + | + | + |
| Mb1731c | ID=Mb1731c; PPE22 Mb1731c, PPE22, len:385 aa. Equivalent to Rv1705c, len: 385 aa, fromMycobacterium tuberculosis strain H37Rv, (99.7%identity in 385 aa overlap). Member of the M. tuberculosis PPE family of glycine-rich proteins, similar to many e.g. YX23_MYCTU|Q10813 hypothetical 41.1 kd protein cy274.2 3 (404 aa), fasta scores: opt: 819, E(): 0, (46.2% identity in 413 aa overlap).; complete gene | + | + | + | + | + | - | + | + | + | + | + | + | + |
| Mb1732c | ID=Mb1732c; PPE23 Mb1732c, PPE23, len:394 aa. Equivalent to Rv1706c, len: 394 aa, fromMycobacterium tuberculosis strain H37Rv, (100%identity in 394 aa overlap). Member of the M. tuberculosis PPE family of glycine-rich proteins, similar to many e.g. YX23_MYCTU|Q10813 hypothetical 41.1 kd protein cy274.23 (404 aa), fasta scores: opt: 841, E(): 3.9e-31, (46.8% identity in 408 aa overlap).; completegene | + | + | + | + | + | + | + | + | + | + | + | + | + |
| Mb1782c | ID=Mb1782c; PPE24 Mb1782c, PPE24, len:1051 aa. Similar to Rv1753c, len: 1053 aa, fromMycobacterium tuberculosis strain H37Rv, (90.7%identity in 1103 aa overlap). Member of the Mycobacterium tuberculosis PPE family of Gly-, Asn-rich proteins, similar to many e.g. YF48_MYCTU|Q10778 hypothetical protein cy48.17 (678 aa), FASTA scores: opt: 1360, E(): 0, (48.9% identity in 550 aa overlap). Note that the Gly-, Asn-rich sequence is interrupted by six near-perfect 26 aa repeats, a unique region, andanother, more degenerate region of five 25 aarepeats before resuming at the C-terminus. The endof the first Gly-, Asn- rich region and the startof the first set of repeats shows some similarityto Q50577|AT10S from Mycobacterium tuberculosis(170 aa) (40.2% identity in 189 aa overlap). REMARK-M.bovis-M.tuberculosis: In Mycobacterium bovis, a 150 bp insertion and a 156 bp deletion, leads to a shorter product compared to its homolog in Mycobacterium tuberculosis strain H37Rv (1051 aa versus 1053 aa).; complete gene | - | - | - | - | - | - | + | + | + | + | + | + | + |
| Mb1788 | ID=Mb1788; cut1 Mb1788, -, len: 218 aa.Similar to Rv1758, len: 174 aa, from Mycobacteriumtuberculosis strain H37Rv, (98.25% identity in 172aa overlap). Probable cut1, serine esterase,cutinase family (EC 3.1.1.-), similar to Rv2301|CUT2_MYCTU|Q50664 probable cutinase cy339.08c precursor from Mycobacterium tuberculosis (219 aa), FASTA scores: opt: 369, E(): 1. 1e-16, (39.1% identity in 179 aa overlap). Also similar to Mycobacterium tuberculosis hypothetical cutinases Rv3452, Rv1984c, Rv3451 andRv3724. CDS has been interrupted by IS6110insertion element and 5'-end deleted. BELONGS TOTHE CUTINASE FAMILY. REMARK-M.bovis-M.tuberculosis: Belongs to the RvD2 region. In Mycobacterium tuberculosis strain H37Rv, Rv1758 is interrupted by IS6110 insertion element and the 5'-end is deleted.; complete gene | + | + | + | + | + | + | + | + | + | + | + | + | + |
| Mb1815 | ID=Mb1815; PPE25 Mb1815, PPE25, len:364 aa. Equivalent to Rv1787, len: 365 aa, fromMycobacterium tuberculosis strain H37Rv, (99.5%identity in 365 aa overlap). Member of the Mycobacterium tuberculosis PPE family of glycine-rich proteins, similar to Z74024|MTCY274.24 Mycobacterium tuberculosis cosmid (404 aa), FASTA scores: opt: 837, E(): 0, (52.0% identity in 406 aa overlap). REMARK-M.bovis-M.tuberculosis: In Mycobacterium bovis, a 8 bp to 5 bp substitution (agcccggt-ccggg), leads to a slightly shorter product compared to its homolog in Mycobacterium tuberculosis strain H37Rv (364 aa versus 365 aa).; complete gene | - | - | - | - | - | - | + | - | - | - | - | + | + |
| Mb1817 | ID=Mb1817; PPE26 Mb1817, PPE26, len:393 aa. Equivalent to Rv1789, len: 393 aa, fromMycobacterium tuberculosis strain H37Rv, (99.7%identity in 393 aa overlap). Member of the Mycobacterium tuberculosis PPE family of glycine-rich proteins, highly similar to others e.g.Z98268|MTCI125.26 Mycobacterium tuberculosis cosmid (385 aa), FASTA score: opt: 1283, E(): 0, (62.7% identity in 408 aa overlap).; complete gene | + | + | + | + | + | + | + | + | + | + | + | + | + |
| Mb1818 | ID=Mb1818; PPE27 Mb1818, PPE27, len:350 aa. Equivalent to Rv1790, len: 350 aa, fromMycobacterium tuberculosis strain H37Rv, (99.7%identity in 350 aa overlap). Member of the Mycobacterium tuberculosis PPE family of glycine-rich protein, similar to Z74024|MTCY274.24 Mycobacterium tuberculosis cosmid (404 aa), FASTA scores: opt: 849, E(): 0, (50.0% identity in 406 aa overlap).; complete gene | - | + | - | - | - | - | + | - | - | - | - | + | + |
| Mb1828 | ID=Mb1828; PPE28 Mb1828, PPE28, len:655 aa. Equivalent to Rv1800, len: 655 aa, fromMycobacterium tuberculosis strain H37Rv, (99.5%identity in 655 aa overlap). Member of the Mycobacterium tuberculosis PPE family of glycine-rich proteins, C-terminal very similar to parts of PE proteins e.g. Z92770|MTCI5.25|Rv0151c (588 aa), FASTA scores: opt: 1269, E(): 0, (41.5% identity in 591 aa overlap).; complete gene | + | + | + | + | + | + | + | + | + | + | + | + | + |
| Mb1829 | ID=Mb1829; PPE29 Mb1829, PPE29, len:423 aa. Equivalent to Rv1801, len: 423 aa, fromMycobacterium tuberculosis strain H37Rv, (100.0%identity in 423 aa overlap). Member of the Mycobacterium tuberculosis PPE family of glycine-rich proteins, most similar to AL022021|MTV049.29|Rv1808 (409 aa), FASTA scores: opt: 1229, E(): 0, (55.2% identity in 422 aa overlap). TBparse score is 0.927.; completegene | + | + | + | + | + | + | + | + | + | + | + | + | + |
| Mb1830 | ID=Mb1830; PPE30 Mb1830, PPE30, len:463 aa. Equivalent to Rv1802, len: 463 aa, fromMycobacterium tuberculosis strain H37Rv, (100.0%identity in 463 aa overlap). Member of the Mycobacterium tuberculosis PPE family of glycine-rich proteins, most similar to AL022021|MTV049.30|Rv1809 (468 aa), FASTA scores: opt: 1238, E(): 0, (51.0% identity in 471 aa overlap).; complete gene | + | + | - | - | - | - | + | + | + | + | + | + | + |
| Mb1836 | ID=Mb1836; PPE31 Mb1836, PPE31, len:399 aa. Equivalent to Rv1807, len: 399 aa, fromMycobacterium tuberculosis strain H37Rv, (99.5%identity in 399 aa overlap). Member of the Mycobacterium tuberculosis PPE family of glycine-rich proteins, most similar to Rv1789|MTV049.11|AL022021 (393 aa), FASTA scores: opt: 1169, E(): 0, (49.5% identity in 399 aa overlap). Start changed since originalsubmission.; complete gene | + | + | + | + | + | + | + | + | + | + | + | + | + |
| Mb1837 | ID=Mb1837; PPE32 Mb1837, PPE32, len:409 aa. Equivalent to Rv1808, len: 409 aa, fromMycobacterium tuberculosis strain H37Rv, (100.000%identity in 409 aa overlap). Member of the Mycobacterium tuberculosis PPE family of glycine-rich proteins, most similar to Rv1800|MTV049.22|AL022021 (655 aa), FASTA scores: opt: 1225, E(): 0, (55.1% identity in 423 aa overlap). Contains PS00343 Gram-positive cocci surface proteins 'anchoring' hexapeptide. TBparsescore is 0.919.; complete gene | + | + | - | + | + | + | + | + | + | + | + | + | + |
| Mb1838 | ID=Mb1838; PPE33a Mb1838, PPE33a, len:187 aa. Equivalent to 5' end of Rv1809, len: 468aa, from Mycobacterium tuberculosis strain H37Rv,(100.0% identity in 187 aa overlap). Member of theMycobacterium tuberculosis PPE family of glycine-rich proteins, most similar to RV1802AL022021|MTV049.23 (463 aa), FASTA scores: opt: 1238, E(): 0, (51.2% identity in 471 aa overlap). REMARK-M.bovis-M.tuberculosis: In Mycobacterium tuberculosis strain H37Rv, PPE33 exists as a single gene. In Mycobacterium bovis, a frameshift due to a single base transition (c-t) splits PPE33 into 2 parts, PPE33a and PPE33b, with PPE33a being truncated.; completegene | + | + | + | + | - | - | + | + | + | + | + | + | + |
| Mb1953c | ID=Mb1953c; PPE35a Mb1953c, PPE35a,len: 625 aa. Equivalent to 5'end of Rv1918c, len:987 aa, from Mycobacterium tuberculosis strainH37Rv, (100.0% identity in 599 aa overlap). Member of the Mycobacterium tuberculosis PPE family of glycine-rich proteins. Similar to MTCY28.16|Z95890 M. tuberculosis cosmid (1053 aa), FASTA scores: opt: 3404, E(): 0, (65.6% identity in 1058 aa overlap). Also similar to MTV004.05, MTY13E10.17, MTV014.03, MTCY3C7.23, MTCY6G11.05, MTCY48.17, MTV004.03, MTCY31.07,MTCY4C12.36, MTCY180.01, etc.REMARK-M.bovis-M.tuberculosis: In Mycobacteriumtuberculosis strain H37Rv, PPE35 exists as a single gene. In Mycobacterium bovis, a frameshift due to a single base insertion (*-t) splits PPE35 into 2 parts, PPE35a and PPE35b.; complete gene | + | + | + | - | - | - | + | + | + | + | + | + | + |
| Mb2036 | ID=Mb2036; Mb2036, -, len: 206 aa.Similar to Rv2013, len: 159 aa, from Mycobacteriumtuberculosis strain H37Rv. Possible transposase:shows similarity to N-terminal part of transposaseand insertion element hypothetical proteins eg sp|Q53198|Y4UE_RHISN PUTATIVE TRANSPOSASE Y4UE (359 aa) opt: 383, E(): 1.3e-18; 35.1% identity in 225 aa overlap; sp|P 14707|YM3_STRCO MINI-CIRCLE HYPOTHETICAL 45.7 KD P (414 aa) opt: 302, E(): 4.2e-13; 33.3% identity in207 aa overlap; and YI90_MYCPA P14322 insertionelement is900 hypothetical protein (399 aa), FASTAscores, opt: 146, E(): 0.0021, (26.9% identity in145 aa overlap). Length changed since firstsubmission (no clear start apparent). REMARK-M.bovis-M.tuberculosis: In Mycobacterium bovis, a single base transition (c-t) at the 5' start of Mb2036, leads to a longer product with a different NH2 part compared toits homolog in Mycobacterium tuberculosis strain H37Rv (206 aa versus 159 aa).; complete gene | + | + | + | + | + | - | + | + | + | + | + | + | + |
| Mb2132 | ID=Mb2132; PPE36 Mb2132, PPE36, len:243 aa. Equivalent to Rv2108, len: 243 aa, fromMycobacterium tuberculosis strain H37Rv, (100.0%identity in 243 aa overlap). N-terminus is similar to N-terminal region of Mycobacterium tuberculosis PPE family proteins eg. YX23_MYCTU Q10813 hypothetical 41.1 kd protein cy274.23 (404 aa), FASTA scores; opt: 431, E(): 3.9e-32, 44.0% identity in 166 aa overlap; complete gene | + | + | + | + | + | + | + | + | + | + | + | + | + |
| Mb2147 | ID=Mb2147; PPE37 Mb2147, PPE37, len:464 aa. Equivalent to Rv2123, len: 473 aa, fromMycobacterium tuberculosis strain H37Rv, (97.9%identity in 473 aa overlap). PPE37 (alternate gene name: irg2), member of the Mycobacterium tuberculosis PPE family of proteins but the C-terminus is not repetitive. REMARK-M.bovis-M.tuberculosis: In Mycobacterium bovis, a 27 bp in-frame deletion leads to a slightly shorter product compared to its homolog in Mycobacterium tuberculosis strain H37Rv (464 aa versus 473 aa).;irg2; complete gene | + | + | + | + | + | + | + | + | + | + | + | + | + |
| Mb2199c | ID=Mb2199c; Mb2199c, -, len: 221 aa.Equivalent to Rv2177c, len: 221 aa, fromMycobacterium tuberculosis strain H37Rv, (99.5%identity in 221 aa overlap). Possible IS1558 transposase (see citation below), similar to several IS element proteins and transposases but nearly identical to last 221 residues of MTCY428_23 (333 aa). FASTA scores: Z81451|MTCY428_23 Mycobacterium tuberculosis cosmid(333 aa) opt: 1491, E() : 0; 98.6% identity in 221aa overlap.; complete gene | + | + | + | + | + | + | + | + | + | + | + | + | + |
| Mb2369c | ID=Mb2369c; PE_PGRS39 Mb2369c,PE_PGRS39, len: 413 aa. Equivalent to Rv2340c,len: 413 aa, from Mycobacterium tuberculosis strain H37Rv, (100.0% identity in 413 aa overlap). Member of the Mycobacterium tuberculosis PE_family, PGRS subfamily of gly-rich proteins, similar to others eg YI18_MYCTU|Q50615|Rv1818c|MTCY1A11.25 PE-PGRS FAMILY PROTEIN from Mycobacterium tuberculosis (498 aa), FASTA scores: opt: 710, E(): 1.4e-22, (41.0% identity in 368aa overlap); O53884|Rv0872v|MTV043.65c PGRS-FAMILYPROTEIN from Mycobacterium tuberculosis (606 aa),FASTA scores: opt: 708, E(): 1.9e-22, (42.4%identity in 389 aa overlap); etc.; completegene | + | + | - | + | + | + | + | + | + | + | + | + | + |
| Mb2376c | ID=Mb2376c; PPE71 Mb2376c, PPE71, len:457 aa. Equivalent to 5' end of MT2423, len: 621aa, from Mycobacterium tuberculosis strainCDC1551, (98.475% identity in 459 aa overlap). PPE FAMILY PROTEIN. REMARK-M.bovis-M.tuberculosis: In Mycobacterium bovis, there is a large 8963 bp deletion (RD5) in between Mb2375c and Mb2377c compared to Mycobacterium tuberculosis strain H37Rv. In this region of Mycobacterium bovis two substitutions are present that encode a CDS, Mb2376c, equivalent to that found in Mycobacterium tuberculosis strain CDC1551. The first substitution is of 1218 bp to 350 bp and the second isof 123 bp to 431 bp.; complete gene | - | + | - | - | - | - | - | + | - | - | - | - | - |
| Mb2447c | ID=Mb2447c; Mb2447c, -, len: 230 aa.Equivalent to 3' end of Rv2424c, len: 333 aa, fromMycobacterium tuberculosis strain H37Rv, (98.7%identity in 230 aa overlap). Probable transposasefor IS1558, similar to IS element proteins e.g.AL021957|Rv2177c|MTV021_10 from Mycobacterium tuberculosis (221 aa), FASTA scores: opt: 1491, E(): 6.2e-87, (98.6% identity in 221 aa overlap); P19780|YIS1_STRCO HYPOTHETICAL INSERTION ELEMENT IS110 from Streptomyces coelicolor (45 aa), FASTA scores: opt: 203, E(): 1.7e-05; (27.3% identity in 238 aa overlap); etc. Contains PS01159 WW/rsp5/WWP domain signature. REMARK-M.bovis-M.tuberculosis: In Mycobacterium tuberculosis strain H37Rv, Rv2424c exists as a single gene. In Mycobacterium bovis, a frameshift due to a 2bp deletion (gt-*) splits Rv2424c into 2 parts,Mb2447c and Mb2448c.; complete gene | + | + | + | + | + | + | + | + | + | + | + | + | + |
| Mb2456c | ID=Mb2456c; PPE41 Mb2456c, PPE41, len:194 aa. Equivalent to Rv2430c, len: 194 aa, fromMycobacterium tuberculosis strain H37Rv, (100.0%identity in 194 aa overlap). Member of the Mycobacterium tuberculosis PPE family similar to others e.g. AAK46014|Rv1745|MT1745 from Mycobacterium tuberculosis (385 aa) FASTA scores: opt: 389, E(): 1.2e-17, (35.95% identity in 192 aa overlap); etc.; complete gene | + | + | + | + | + | + | + | + | + | + | + | + | + |
| Mb2540c | ID=Mb2540c; Mb2540c, -, len: 415 aa.Equivalent to Rv2512c, len: 415 aa, fromMycobacterium tuberculosis strain H37Rv, (100.0%identity in 415 aa overlap). Transposase for IS1081, identical to P35882|TRA1_MYCBO transposase for insertion sequence element IS1081 from Mycobacterium bovis (415 aa), FASTA scores: opt: 2680, E(): 1.9e-162, (100.0% identity in 415 aa overlap). Also highly similar toothers from Mycobacterium tuberculosis e.g. P96354|Rv1047|MTCY10G2.02c|Rv3115|MTCY164.25|Rv3023c|MTV01 2 .38c (415 aa), FASTA scores: opt: 2675, E(): 3.9e-162, (99.75% identity in 415 aa overlap). Contains PS00435 Peroxidases proximal heme-ligand signature, PS01007 Transposases, Mutator family, signature. BELONGS TOTHE MUTATOR FAMILY OF TRANSPOSASE.; completegene | - | - | - | - | - | - | + | - | - | - | - | + | + |
| Mb2640 | ID=Mb2640; PPE42 Mb2640, PPE42, len:580 aa. Equivalent to Rv2608, len: 580 aa, fromMycobacterium tuberculosis strain H37Rv, (99.8%identity in 580 aa overlap). Member of the Mycobacterium tuberculosis PPE family, highly similar to many e.g. O06828|Rv1430|MTCY493.24c from Mycobacterium tuberculosis (528 aa), FASTA scores: opt: 1004, E(): 5.9e-48, (56.05% identity in 307 aa overlap).; completegene | + | + | + | + | + | + | + | + | + | + | + | + | + |
| Mb2790c | ID=Mb2790c; PPE43 Mb2790c, PPE43, len:394 aa. Equivalent to Rv2768c, len: 394 aa, fromMycobacterium tuberculosis strain H37Rv, (99.7%identity in 394 aa overlap). Member of the Mycobacterium tuberculosis PPE family, highly similar to many e.g. upstream ORF O33312|Rv2770c|MTV002.35c (402 aa), FASTA scores: opt: 1135, E(): 6.1e-51, (62.15% identityin 391 aa overlap); and P96362|Rv1039c|MTCY10G2.10from M. tuberculosis (391 aa), FASTA scores: opt:1721, E(): 6.8e-81, (70.35% identity in 398 aaoverlap). Equivalent to AAK47157 fromMycobacterium tuberculosis strain CDC1551 (462 aa)but shorter 68 aa.; complete gene | + | + | + | + | + | + | + | + | + | + | + | + | + |
| Mb2792c | ID=Mb2792c; PPE44 Mb2792c, PPE44, len:382 aa. Equivalent to Rv2770c, len: 382 aa, fromMycobacterium tuberculosis strain H37Rv, (99.7%identity in 382 aa overlap). Member of the Mycobacterium tuberculosis PPE family, highly similar to many e.g. downstream ORF O33310|Rv2768c|MTV002.33c from M. tuberculosis (394 aa), FASTA scores: opt: 1135, E(): 2.2e-53, (62.15% identity in 391 aa overlap); and P96362|Rv1039c|MTCY10G2.10 from Mycobacteriumtuberculosis (391 aa), FASTA scores: opt: 1010,E(): 1e-46, (55.95% identity in 395 aa overlap).Equivalent to AAK47159 from M. tuberculosis strainCDC1551 (402 aa) but shorter 20 aa. Start changedsince first submission (-20 aa).; complete gene | + | + | + | + | + | + | + | + | + | + | + | + | + |
| Mb2814c | ID=Mb2814c; Mb2814c, -, len: 459 aa.Equivalent to Rv2791c, len: 459 aa, fromMycobacterium tuberculosis strain H37Rv, (100.0%identity in 459 aa overlap). Probable IS1602 transposase for IS1602 element, similar to many e.g. P95117|Rv2978c|MTCY349.09 from Mycobacterium tuberculosis (459 aa), FASTA scores: opt: 2718, E(): 6.3e-165, (86.05% identity in 459 aa overlap).; complete gene | + | + | + | + | + | + | + | + | + | + | + | + | + |
| Mb2916c | ID=Mb2916c; PPE45 Mb2916c, PPE45, len:408 aa. Equivalent to Rv2892c, len: 408 aa, fromMycobacterium tuberculosis strain H37Rv, (100.0%identity in 408 aa overlap). Member of the Mycobacterium tuberculosis PPE family, highly similar to many e.g. O06386|Rv3621c|MTCY15C10.31|MTCY07H7B.01 from M. tuberculosis (413 aa), FASTA scores: opt: 957, E(): 6.2e-46, (44.7% identity in 423 aa overlap).; complete gene | + | + | + | + | + | + | + | + | + | + | + | + | + |
| Mb3043c | ID=Mb3043c; PPE46 Mb3043c, PPE46, len:437 aa. Equivalent to Rv3018c, len: 434 aa, fromMycobacterium tuberculosis strain H37Rv, (99.5%identity in 431 aa overlap). Member of PPE family but lacks Gly, Ala rich repeats at C-terminal domain, closest to MTCY261.19. Also very similar to following ORF MTV012.35c. Nearly identical in parts to Mycobacterium tuberculosis protein erroneously described as DIHYDROFOLATE REDUCTASE (X59271|MTFOLA_1) P31500|DYR_MYCTU (214 aa), FASTA scores: opt: 972, E(): 4.4e-42,(80.0% identity in 195 aa overlap); andZ97559|MTCY261_19 from M. tuberculosis cosmid (473aa), FASTA scores: opt: 806, E(): 0; (38.8%identity in 479 aa overlap); and O53268|MTV012.35cfrom Mycobacterium tuberculosis (358 aa), FASTAscores: opt: 1714, E(): 3.3e-79, (78.3% identityin 355 aa overlap). REMARK-M.bovis-M.tuberculosis:In Mycobacterium bovis, a 56 bp deletion leads toa product slightly different at the Nh2-terminuspart compared to its homolog in Mycobacteriumtuberculosis strain H37Rv (434 aa versus 437 aa).; complete gene | + | - | - | - | + | - | + | - | - | - | - | + | + |
| Mb3047c | ID=Mb3047c; PPE47 Mb3047c, PPE47, len:435 aa. Equivalent to Rv3022c (PPE48) and Rv3021c(PPE47), len: 81 aa and 358 aa, from Mycobacteriumtuberculosis strain H37Rv, (98.8% identity in 81aa overlap and 98.6% identity in 354 aa overlap). Member of Mycobacterium tuberculosis PPE family. Should be continuation of upstream ORF MTV012.36c but is frameshifted due to missing base at 36448 in v012. Sequence has been checked but no error apparent. Very similar to neighbouring ORF O53265|MTV012.32c|Rv3018c from Mycobacterium tuberculosis (434 aa), FASTA scores: opt: 1714, E(): 6.6e-770, (78.3% identity in 355 aaoverlap) and AAK47430|MT3101 (strongly in theN-terminal part) (310 aa), FASTA scores: opt: 897,E(): 4.5e-37, (66.95% identity in 227 aa overlap). REMARK-M.bovis-M.tuberculosis: In Mycobacterium tuberculosis H37Rv, PPE47 and PPE48 exist as 2separate genes. In Mycobacterium bovis, a singlebase insertion (*-g) leads to a single product.; complete gene | + | - | - | - | + | - | + | - | - | - | - | + | + |
| Mb3069c | ID=Mb3069c; ctaD Mb3069c, ctaD, len:573 aa. Equivalent to Rv3043c, len: 573 aa, fromMycobacterium tuberculosis strain H37Rv, (100.0%identity in 573 aa overlap). Probable ctaD, integral membrane cytochrome C oxidase polypeptide I (EC 1.9.3.1), equivalent to Q9CBQ5|ML1728 from Mycobacterium leprae (574 aa), FASTA scores: opt: 3738, E(): 3.8e-216, (95.4% identity in 566 aa overlap). Also similar toother CYTOCHROME C OXIDASES POLYPEPTIDE I e.g.Q9AEL9|CTAD from Corynebacterium glutamicum(Brevibacterium flavum) (584 aa), FASTA scores:opt: 3065, E(): 6.8e-176, (72.65% identity in 567aa overlap); Q9X813|SC6G10.28c from Streptomycescoelicolor (578 aa), FASTA scores: opt: 2888, E():2.6e-165, (71.7% identity in 544 aa overlap); Q9K451|CTAD from Streptomyces coelicolor (573 aa), FASTA scores: opt: 2757, E(): 1.8e-157, (70.2% identity in 537 aa overlap). Contains PS00077 Cytochrome c oxidase subunit I, copper B binding region signature. BELONGS TOTHE HEME-COPPER RESPIRATORY OXIDASE FAMILY.; complete gene | + | + | + | + | + | + | + | + | + | + | + | + | + |
| Mb3124c | ID=Mb3124c; PE_PGRS63 Mb3124c,PE_PGRS63, len: 437 aa. Equivalent to Rv3097c,len: 437 aa, from Mycobacterium tuberculosis strain H37Rv, (99.8% identity in 437 aa overlap). Probable Triacylglycerol lipase (EC 3.1.1.3), and member of the M. tuberculosis PE-family PGRS subfamily of gly-rich proteins; N-terminal part similar to N-terminus of M. tuberculosis PE-PGRS family members e.g.Q10637|Y03A_MYCTU hypothetical glycine-rich 49.6kd protein (603 aa). Other relatives includeMTCY1A11.25c; MTCY21B4.13c; MTCY270.06; MTCY359.33; MTC1A11.04.; complete gene | + | + | + | + | + | + | + | + | + | + | + | + | + |
| Mb3148c | ID=Mb3148c; PPE49 Mb3148c, PPE49, len:391 aa. Equivalent to Rv3125c, len: 391 aa, fromMycobacterium tuberculosis strain H37Rv, (99.7%identity in 391 aa overlap). Member of the M. tuberculosis PPE family, similar to other e.g. P95247|Rv2352c|MTCY98.21c (391 aa), FASTA scores: opt: 1576, E(): 3.8e-72, (62.55% identity in 398 aa overlap), MTCY98.0029c, MTCY03A2.22c, MTCY10G2.10, MTCY02B10.25c, MTCI364.08, M TCY21C12.09c, MTCY48.17.; completegene | + | + | + | + | + | + | + | + | + | + | + | + | + |
| Mb3159 | ID=Mb3159; PPE50 Mb3159, PPE50, len:381 aa. Similar to 5' end of Rv3135, len: 132 aa,from Mycobacterium tuberculosis strain H37Rv,(88.5% identity in 131 aa overlap). Member of theMycobacterium tuberculosis Ala-, Gly-rich PPE family, similar to P95190|Rv3136|MTCY03A2.22c (380 aa), FASTA scores: opt: 494, E(): 6.7e-25, (57.25% identity in 131 aa overlap) (next ORF downstream), MTY21C12_9, MTCY3C7_24, MTCI125_27, MTV049_12, MTV049_9, MTV049_11 , MTCY274_24 etc. REMARK-M.bovis-M.tuberculosis: In Mycobacterium bovis, a large 1337 bp insertion leadsto a longer product with a different COOH partcompared to its homolog in Mycobacteriumtuberculosis strain H37Rv (381 aa versus 132 aa).; complete gene | + | + | + | + | + | + | + | + | + | + | + | + | + |
| Mb3160 | ID=Mb3160; PPE51 Mb3160, PPE51, len:380 aa. Equivalent to Rv3136, len: 380 aa, fromMycobacterium tuberculosis strain H37Rv, (100.0%identity in 380 aa overlap). Member of the Mycobacterium tuberculosis Ala-, Gly-rich PPE family, similar to Q9AGF0|Ov2770c Rv2770c-LIKE PROTEIN from M. microti (397 aa), FASTA scores: opt: 917, E(): 9e-41, (46.15% identity in 388 aa overlap); O33312|Rv2770c|MTV002.35c, MTV002_36, MTCI125_26, MTCY10G2_10, MTCI364_8, MTV049_28, MTV049_29, etc. TBparse score is 0.923.; complete gene | + | + | + | + | + | + | + | + | + | + | + | + | + |
| Mb3168c | ID=Mb3168c; PPE52 Mb3168c, PPE52, len:409 aa. Equivalent to Rv3144c, len: 409 aa, fromMycobacterium tuberculosis strain H37Rv, (99.5%identity in 409 aa overlap). Member of the M. tuberculosis PPE family, Gly-, Ala-rich, similar to others e.g. P71868|Rv3533c|MTCY03C7.23 (582 aa), FASTA scores: opt: 1007, E(): 5.2e-35, (56.2% identity in 306 aa overlap); and MTV014_3, MTCY6G11_5, MTCY98.0034c, MTCY31.06c, MTCY48.17, MTCY98.0029c, MTCY03C7.17c, etc.; complete gene | + | + | + | + | + | + | + | + | + | + | + | + | + |
| Mb3183c | ID=Mb3183c; PPE53 Mb3183c, PPE53, len:589 aa. Equivalent to Rv3159c, len: 590 aa, fromMycobacterium tuberculosis strain H37Rv, (99.7%identity in 590 aa overlap). Member of the Mycobacterium tuberculosis PPE_family of Gly-, Asn-rich proteins. Highly similar to P71868|Rv3533c|MTCY03C7.23 (582 aa), FASTA scores: opt: 2289, E(): 3.2e-98, (63.5% identity in 600 aa overlap); and also similar to MTCY48_17, MTV041_29, MTCY6G11_5, MTCY98_24, etc. TBparse score is 0.921. REMARK-M.bovis-M.tuberculosis: In Mycobacterium bovis, albeit a 2143 bp insertionoccurs overlapping the NH2-terminal part, thisleads to an equivalent product, compared to itshomolog in Mycobacterium tuberculosis strainH37Rv.; complete gene | + | + | + | + | + | + | + | + | + | - | + | + | + |
| Mb3184c | ID=Mb3184c; PPE70 Mb3184c, PPE70, len:685 aa. Equivalent to MT3248, len: 686 aa, fromMycobacterium tuberculosis strain CDC1551,(99.708% identity in 686 aa overlap). REMARK-M.bovis-M.tuberculosis: In Mycobacterium bovis, an insertion of 2143 bp exists between PPE53 and Rv3160c compared to Mycobacterium tuberculosis strain H37Rv. This leads to a additional gene, PPE70 equivalent toMT3248 from Mycobacterium tuberculosis strainCDC1551.; complete gene | + | + | + | + | + | - | + | - | + | + | + | + | + |
| Mb3249c | ID=Mb3249c; Mb3249c, -, len: 183 aa.Equivalent to Rv3222c, len: 183 aa, fromMycobacterium tuberculosis strain H37Rv, (100.0%identity in 183 aa overlap). Hypothetical protein, with some similarity to Q9SZD2|F19B15.50|AT4G29020 GLYCINE-RICH PROTEIN LIKE from Arabidopsis thaliana (Mouse-ear cress) (158 aa), FASTA scores: opt: 131, E(): 0.77, (33.35% identity in 126 aa overlap); Q9S222|SCI51.18 PUTATIVE TRANSCRIPTIONAL REGULATOR from Streptomyces coelicolor (548 aa), FASTA scores: opt: 133, E():1.6, (36.25% identity in 149 aa overlap); etc.Also some similarity to other hypotheticalMycobacterium tuberculosis proteins e.g.O06292|Rv0341|MTCY13E10.01 (479 aa), FASTA scores:opt: 141, E(): 0.5, (31.2% identity in 170 aaoverlap); AAK45760|MT1497.1 PE_PGRS FAMILY PROTEIN from strain CDC1551 (1408 aa), FASTA scores: opt: 137, E(): 2, (31.75% identity in 148 aa overlap); etc.; completegene | + | + | + | + | + | + | + | + | + | + | + | + | + |
| Mb3375c | ID=Mb3375c; PPE54 Mb3375c, PPE54, len:1338 aa. Similar to 3' end of Rv3343c, len: 2523aa, from Mycobacterium tuberculosis strain H37Rv,(98.6% identity in 1151 aa overlap). Member of theMycobacterium tuberculosis PPE family, MPTR subgroup of Gly-, Asn-rich proteins. Most similar to O50379|Rv3350c|MTV004.07c|MTV004_5 from Mycobacterium tuberculosis strain H37Rv (3716 aa), FASTA scores: opt: 4672, E(): 4e-211, (44.2% identity in 3174 aa overlap); and also similar to MTV004_3, MTCY63_9, MTY13E10_17, MTY13E10_16, MTCY180_1, MTV050_1, MTCY3C7_23,MTV014_3, MTCY63_10; etc.REMARK-M.bovis-M.tuberculosis: In Mycobacteriumbovis, a 3555 bp deletion leads to a shorter product compared to its homolog in Mycobacterium tuberculosis strain H37Rv.; complete gene | - | - | - | - | - | - | + | - | - | - | - | + | + |
| Mb3380c | ID=Mb3380c; PPE55a Mb3380c, PPE55a,len: 2096 aa. Similar to 5' end of Rv3347c, len:3157 aa, from Mycobacterium tuberculosis strainH37Rv, (99.5% identity in 2044 aa overlap). Member of the Mycobacterium tuberculosis PPE family, Gly-, Ala-, Asn-rich protein. Similar to many from Mycobacterium tuberculosis strains H37Rv and CDC1551, e.g. O50379|Rv3350c|MTV004.07c (3716 aa), FASTA scores: opt: 6497, E(): 0, (61.65% identity in 3756 aa overlap); and other upstream ORFs MTV004_5, MTY13E10_15, MTCY28_16, MTCY63_9, MTY13E10_17, MTCY180_1; etc. REMARK-M.bovis-M.tuberculosis: In Mycobacterium tuberculosis strain H37Rv, PPE55 exists as a single gene. In Mycobacterium bovis, a frameshift due to a single base deletion (g-*) splits PPE55 into 2 parts, PPE55a and PPE55b.; complete gene | + | + | - | - | - | - | + | + | - | - | - | + | + |
| Mb3385c | ID=Mb3385c; PPE56a Mb3385c, PPE56a,len: 434 aa. Equivalent to 5' end of Rv3350c, len:3716 aa, from Mycobacterium tuberculosis strainH37Rv, (100.0% identity in 434 aa overlap). Member of the Mycobacterium tuberculosis PPE family of Gly-, Ala-, Asn-rich proteins, similar to many Mycobacterium tuberculosis proteins from strains H37Rv and CDC1551, e.g. O50378|Rv3347c|MTV004.03c (3157 aa), FASTA scores: opt: 6497, E(): 0, (61.65% identity in 3756 aaoverlap); MTCY28_16, MTV050_2, MTY13E10_17,MTCY63_10, MTCY180_1, MTCY63_9, MTV050_1,MTV014_3, MTY13E10_15; etc. REMARK-M.bovis-M.tuberculosis: In Mycobacterium tuberculosis strain H37Rv, PPE56 exists as a single gene. In Mycobacterium bovis, 2 frameshifts due to single base transversion (c-a) and a single base deletion (g-*)splits PPE56 into 3 parts, PPE56a, PPE56b andPPE56d.; complete gene | + | + | - | + | + | + | + | + | - | + | + | + | + |
| Mb3418 | ID=Mb3418; Mb3418, -, len: 234 aa.Equivalent to Rv3386, len: 234 aa, fromMycobacterium tuberculosis strain H37Rv, (100.0%identity in 234 aa overlap). Possible transposase, showing very weak similarity to several IS element transposases. Highly similar (but shorter) to P963659|MTCY10G2_13|Rv1036c from Mycobacterium tuberculosis (112 aa), FASTA scores: opt: 507, E(): 8.3e-25, (83.9% identity in 87 aa overlap).; complete gene | + | + | + | + | + | + | + | + | + | + | + | + | + |
| Mb3419 | ID=Mb3419; Mb3419, -, len: 225 aa.Equivalent to Rv3387, len: 225 aa, fromMycobacterium tuberculosis strain H37Rv, (100.0%identity in 225 aa overlap). Possible transposase, showing very weak similarity to other IS element proteins, and similar to various hypothetical proteins.; completegene | + | + | + | + | + | + | + | + | + | + | + | + | + |
| Mb3459 | ID=Mb3459; PPE57 Mb3459, PPE57, len:178 aa. Equivalent to 5' end of Rv3425, len: 176aa, from Mycobacterium tuberculosis strain H37Rv,(90.9% identity in 176 aa overlap). Member of theMycobacterium tuberculosis PPE family, similar to many e.g. O06246|Rv3429|MTCY77.01 (178 aa), FASTA scores: opt: 781, E(): 7e-44, (69.9% identity in 176 aa overlap); and downstream Q50702|YY26_MYCTU|Rv3426|MTCY78.03c (232 aa), FASTA scores: opt: 517, E(): 1.2e-26, (68.0%identity in 125 aa overlap); MTV049_11,MTCY428_16, MTV049_22, MTV049_30, MTCY261_4; etc.Rv3429: Member of the M. tuberculosis PPE family,similar to many e.g. the upstream Q50703|YY25_MYCTU|Rv3425|MTCY78.04c (176 aa), FASTA scores: opt: 781, E(): 1.9e-44, (69.9% identity in 176 aa overlap); and Q50702|YY26_MYCTU|Rv3426|MTCY78.03c (232 aa), FASTA scores: opt: 555, E(): 1.7e-29, (72.0% identity in 125 aa overlap) (but diverges at 3' end)); etc. REMARK-M.bovis-M.tuberculosis: In Mycobacteriumbovis, a large deletion of 4926 bp (RD6) leads tothe loss of the COOH part of PPE57, and thefollowing CDSs, PPE58, Rv3427c, Rv3428c and PPE59compared to Mycobacterium tuberculosis strainH37Rv.; complete gene | + | + | + | + | + | + | + | + | + | + | + | + | + |
| Mb3505 | ID=Mb3505; PPE60 Mb3505, PPE60, len:393 aa. Equivalent to Rv3478, len: 393 aa, fromMycobacterium tuberculosis strain H37Rv, (100.0%identity in 393 aa overlap). PPE60 (alternate gene name: mtb39c). Member of the M. tuberculosis PPE family, highly similar to others e.g. Q11031|YD61_MYCTU|Rv1361c|MT1406|MTCY02B10.25c (396 aa), FASTA scores: opt: 2165, E(): 1.1e-109, (85.35% identity in 396 aa overlap); MTCI364.08; MTCY10G2.10; MTCY03A2.22c; MTCY274.23c; MTCY164.34c; MTCY98.0029c; etc. Notethat expression of Rv3478 was demonstrated inlysates by immunodetection (see citation below).;mtb39c; complete gene | - | - | - | - | + | - | + | - | - | + | - | + | + |
| Mb3562 | ID=Mb3562; PPE61 Mb3562, PPE61, len:406 aa. Equivalent to Rv3532, len: 406 aa, fromMycobacterium tuberculosis strain H37Rv, (100.0%identity in 406 aa overlap). Member of the Mycobacterium tuberculosis PPE protein family, similar to many, e.g. O53956|Rv1807|MTV049.29 (403 aa), FASTA scores: opt: 954, E(): 1.1e-43, (44.1% identity in 417 aa overlap); complete gene | + | + | + | + | + | + | + | + | + | + | + | + | + |
| Mb3563c | ID=Mb3563c; PPE62 Mb3563c, PPE62, len:582 aa. Equivalent to Rv3533c, len: 582 aa, fromMycobacterium tuberculosis strain H37Rv, (100.0%identity in 582 aa overlap). Member of the Mycobacterium tuberculosis PPE protein family, similar to many, e.g. O53309|Rv3159c|MTV014.03c (590 aa) FASTA scores: opt: 2289, E(): 2.3e-95, (63.5% identity in 600 aa overlap).; complete gene | + | + | + | + | + | + | + | + | + | + | + | + | + |
| Mb3569 | ID=Mb3569; PPE63 Mb3569, PPE63, len:479 aa. Equivalent to Rv3539, len: 479 aa, fromMycobacterium tuberculosis strain H37Rv, (100.0%identity in 479 aa overlap). Member of the Mycobacterium tuberculosis PPE protein family, similar to many e.g. O53949|Rv1800|MTV049.22 (655 aa), FASTA scores: opt: 914, E(): 7.3e-47, (37.55% identity in 490 aa overlap); etc.; complete gene | + | + | + | + | + | + | + | + | + | + | + | + | + |
| Mb3827 | ID=Mb3827; Mb3827, -, len: 205 aa.Equivalent to 5' end of Rv3798, len: 444 aa, fromMycobacterium tuberculosis strain H37Rv, (99.5%identity in 195 aa overlap). Putative transposasefor insertion sequence element IS1557, highly similar to Q60255 SIMILAR TO TRANSPOSASE OF ISAE1 FROM ALCALIGENES EUTROPHUS H1-4 (FRAGMENT) from dibenzofuran-degrading bacterium DPO360 (163 aa) FASTA scores: opt: 767, E(): 3.2e-42, (67.25% identity in 168 aa overlap); and similar to P74920 TRANSPOSASE from Thiobacillus ferrooxidans (404 aa), FASTA scores: opt: 375, E(): 1.1e-16, (27.55% identity in 439 aa overlap); Q48349 TRANSPOSASE from Alcaligenes eutrophus(Ralstonia eutropha) (408 aa), FASTA scores: opt:324, E(): 2e-13, (3.9% identity in 369 aaoverlap); Q9FDC1|TNP TRANSPOSASE from Burkholderiamallei (Pseudomonas mallei) (386 aa) FASTA scores:opt: 282, E(): 9.8e-11, (25.85% identity in 391 aaoverlap); etc. C-terminal end identical to O53804|Rv0741|MTV041.15 TRANSPOSASE from Mycobacterium tuberculosis (104 aa), FASTA scores: opt: 582, E(): 1.8e-30, (85.6% identity in 104 aa overlap). BELONGS TO THE TRANSPOSASE FAMILY 12. REMARK-M.bovis-M.tuberculosis: In Mycobacterium tuberculosis strain H37Rv, Rv3798exists as a single gene. In Mycobacterium bovis, aframeshift due to a 11 bp deletion splits Rv3798into 2 parts, Mb3827 and Mb3828.; complete gene | + | + | + | + | + | + | + | - | - | - | - | + | + |
| Mb3828 | ID=Mb3828; Mb3828, -, len: 243 aa.Equivalent to 3' end of Rv3798, len: 444 aa, fromMycobacterium tuberculosis strain H37Rv, (100.0%identity in 243 aa overlap). Putative transposasefor insertion sequence element IS1557, highlysimilar to Q60255 SIMILAR TO TRANSPOSASE OF ISAE1FROM ALCALIGENES EUTROPHUS H1-4 (FRAGMENT) from dibenzofuran-degrading bacterium DPO360 (163 aa) FASTA scores: opt: 767, E(): 3.2e-42, (67.25% identity in 168 aa overlap); and similar to P74920 TRANSPOSASE from Thiobacillus ferrooxidans (404 aa), FASTA scores: opt: 375, E(): 1.1e-16, (27.55% identity in 439 aa overlap); Q48349 TRANSPOSASE from Alcaligenes eutrophus(Ralstonia eutropha) (408 aa), FASTA scores: opt:324, E(): 2e-13, (3.9% identity in 369 aaoverlap); Q9FDC1|TNP TRANSPOSASE from Burkholderiamallei (Pseudomonas mallei) (386 aa) FASTA scores:opt: 282, E(): 9.8e-11, (25.85% identity in 391 aaoverlap); etc. C-terminal end identical to O53804|Rv0741|MTV041.15 TRANSPOSASE from Mycobacterium tuberculosis (104 aa), FASTA scores: opt: 582, E(): 1.8e-30, (85.6% identity in 104 aa overlap). BELONGS TO THE TRANSPOSASE FAMILY 12. REMARK-M.bovis-M.tuberculosis: In Mycobacterium tuberculosis strain H37Rv, Rv3798exists as a single gene. In Mycobacterium bovis, aframeshift due to a 11 bp deletion splits Rv3798into 2 parts, Mb3827 and Mb3828.; complete gene | + | + | + | + | + | + | + | - | - | - | - | + | + |
| Mb3842 | ID=Mb3842; PE_PGRS62 Mb3842, PE_PGRS62,len: 504 aa. Equivalent to Rv3812, len: 504 aa,from Mycobacterium tuberculosis strain H37Rv,(100.0% identity in 504 aa overlap). Member of theMycobacterium tuberculosis PE family, PGRS subfamily of gly-rich proteins, similar to many e.g. P96828|Rv0151c|MTCI5.25c (588 aa), FASTA scores: opt: 389, E(): 6.2e-14, (29.2% identity in 473 aa overlap); MTCY7H7B_27; MTCY493_24; MTCY441_4; MTCY39_36; MTCY1A11_4; MTCY359_33; MTCY130_10; MTCY98_9; etc.; completegene | + | + | + | + | + | + | + | + | + | + | + | + | + |
| Mb3852 | ID=Mb3852; Mb3852, -, len: 404 aa.Equivalent to Rv3822, len: 404 aa, fromMycobacterium tuberculosis strain H37Rv, (99.3%identity in 404 aa overlap). Conserved hypothetical protein, similar in part to hypothetical proteins from Mycobacterium leprae: Q9CC62|ML1232 (358 aa) FASTA scores: opt: 601, E(): 1.1e-25, (36.7% identity in 335 aa overlap); and Q49633|B1170_F3_112 (391 aa) FASTA scores: opt: 601, E(): 1.2e-25, (36.25% identity in 347 aa overlap). Also similar to P71862|Rv3539|MTCY03C7.17cPPE FAMILY PROTEIN from Mycobacterium tuberculosis(479 aa), FASTA scores: opt: 547, E(): 1.3e-22,(38.1% identity in 281 aa overlap);O50440|Rv1184c|MTV005.20c (359 aa); O06828|Rv1430|MTCY493.24c (528 aa); O53642|Rv0159c|MTV032.02c (468 aa); etc.; complete gene | + | + | + | + | + | + | + | + | + | + | + | + | + |
| Mb3857c | ID=Mb3857c; Mb3857c, -, len: 408 aa.Equivalent to Rv3827c, len: 408 aa, fromMycobacterium tuberculosis strain H37Rv, (99.8%identity in 408 aa overlap). Possible transposase within IS1537 element, similar to several transposases e.g. O83029|TNPC|DR2324|DR0666|DR0978|DR1381|DR1651|DR1933 TRANSPOSASE from Deinococcus radiodurans(408 aa) FASTA scores: opt: 302, E(): 3.9e-12, (30.75% identity in358 aa overlap); Q9RXX7|DR0178 PUTATIVETRANSPOSASE from Deinococcus radiodurans (409 aa),FASTA scores: opt: 297, E(): 8.2e-12, (31.1%identity in 360 aa overlap); P73816|SLR2062TRANSPOSASE from Synechocystis sp. strain PCC 6803(400 aa), FASTA scores: opt: 296, E(): 9.3e-12, (30.05% identity in 353 aa overlap); etc. Highly similar to proteins from Mycobacterium tuberculosis e.g. O33333|Rv2791c|MTV002.56c TRANSPOSASE (459 aa) FASTA scores: opt: 2211, E(): 9.4e-136, (87.75% identity in 367 aa overlap); P95117|Rv2978c|MTCY349.09 HYPOTHETICAL 51.4 KDA PROTEIN (459 aa), FASTA scores: opt: 2165, E(): 9e-133, (85.85% identity in 367 aa overlap); Q10809|YS85_MYCTU|Rv2885c|MT2953|MTCY274.16cHYPOTHETICAL 51.3 KDA PROTEIN (460 aa), FASTAscores: opt: 2127, E(): 2.6e-130, (83.95% identityin 368 aa overlap); O0777|Rv0606|MTCY19H5.16cPROBABLE TRANSPOSASE (FRAGMENT) (247 aa), FASTAscores: opt: 1405, E(): 9.3e-84, (85.3% identityin 238 aa overlap); etc.; complete gene | + | + | + | + | + | + | + | + | + | + | + | + | + |
| Mb3858c | ID=Mb3858c; Mb3858c, -, len: 203 aa.Equivalent to Rv3828c, len 203 aa, fromMycobacterium tuberculosis strain H37Rv, (100.0%identity in 203 aa overlap). Possible resolvase within IS1537 element, similar to others e.g. Q97X40|SSO1915 FIRST ORF IN TRANSPOSON ISC1913 from Sulfolobus solfataricus (213 aa), FASTA scores: opt: 275, E(): 1.6e-11, (30.6% identity in 196 aa overlap); Q9V1M0|PAB2076 RESOLVASE RELATED PROTEIN from Pyrococcus abyssi (212 aa), FASTA scores: opt: 254, E(): 4.2e-10, (29.95% identity in 197 aa overlap); Q9RMU7|ORFAPUTATIVE TRANSPOSASE (BELONGS TO THE MERR FAMILYOF TRANSCRIPTIONAL REGULATORS) from elicobacterpylori (Campylobacter pylori) (217 aa), FASTAscores: opt: 243, E(): 2.3e-09, (31.8% identity in154 aa overlap); etc. Also highly similar to proteins from Mycobacterium tuberculosis e.g. O33334|Rv2792c|MTV002.57c RESOLVASE (193 aa), FASTA scores: opt: 970, E(): 1.5e-58, (79.25% identity in 193 aa overlap); O07773|Rv0605|MTCY19H5.17c PUTATIVE RESOLVASE (202 aa), FASTA scores: opt: 964, E(): 4e-58, (76.25% identity in 202 aa overlap);P95116|Rv2979c|MTCY349.08 HYPOTHETICAL 21.4 KDAPROTEIN (194 aa), FASTA scores: opt: 895, E():1.8e-53, (74.75% identity in 194 aa overlap); Q10831|YS86_MYCTU|Rv2886c|MT2954|MTCY274.17c HYPOTHETICAL 31.9 KDA PROTEIN (295 aa), FASTA scores: opt: 826, E(): 1.1e-48, (66.2% identity in 204 aa overlap) (similarity only at C-terminus); etc. Contains PS00397Site-specific recombinases active site. Possiblehelix-turn-helix motif from aa 11-32, Score 1305(+3.63 SD).; complete gene | + | + | + | + | + | + | + | + | + | + | + | + | + |
